# Bimetallic Nanozymes/Polypyrrole/Methylene Blue Platform for Photothermal and Catalytic Biofilm Disruption and Angiogenesis Enhancement in Diabetic Wound Healing

**DOI:** 10.1002/smsc.202500445

**Published:** 2026-02-03

**Authors:** Prafful P. Kothari, Tonmoy Banerjee, Balaram Ghosh, Swati Biswas

**Affiliations:** ^1^ Nanomedicine Research Laboratory Department of Pharmacy Birla Institute of Technology and Science‐Pilani Hyderabad Campus, Medchal Hyderabad Telangana 500078 India

**Keywords:** antibiofilm activity, ceria‐zinc nanoflowers, diabetic wound healing, methicillin‐resistant Staphylococcus aureus, photothermal

## Abstract

Diabetic wounds pose a significant challenge due to impaired tissue regeneration, prolonged inflammation, poor oxygen supply, and microbial infections. Methicillin‐resistant *Staphylococcus aureus* (MRSA) infections delay healing by prolonging inflammation and increasing antimicrobial resistance. To develop an effective antibiotic alternative, multifunctional nanocomposites, ceria‐zinc nanoflowers (CeZn@PPY@MB NFs) bearing a polypyrrole (PPY) coating loaded with methylene blue (MB) are developed, to address the multifaceted requirements for healing diabetic wounds. Due to the synergistic effects of photothermal, catalysis, and reactive oxygen species (ROS) generation,CeZn@PPY@MB NFs exhibit robust antibacterial activity with high collagen deposition and angiogenesis. The nanoflowers, with a high surface area of water lily‐like morphology, are confirmed through scanning electron microscopy, energy‐dispersive X‐ray analysis, X‐ray photoelectron spectroscopy, and X‐ray diffraction analysis. PPY/MB‐mediated synergistic photothermal effect, ROS generation, and catalytic activities lead to robust MRSA killing and biofilm disruption. Nanoflowers demonstrate rapid wound healing due to reduced inflammation, tissue regeneration, and angiogenesis in the diabetes‐induced wound model by modulating tumor necrosis factor‐α, CD‐44, Ki‐67, collagen deposition, ROS, interleukin‐1β (IL‐1β), IL‐8, and IL‐6 expression. Therefore, the developed multifunctional hybrid‐metallic nanoflowers provide an advanced nanotherapeutic platform to eradicate MRSA effectively, offering a promising alternative to antibiotic therapy for managing resistant bacteria‐infected diabetic foot ulcers.

## Graphical abstract

The bimetallic ceria‐zinc nanoflowers coated with polypyrrole and loaded with methylene blue disrupt methicillin‐resistant *Staphylococcus aureus* biofilms synergistically, promote angiogenesis, thereby achieving efficient diabetic wound healing. Integrated photothermal therapy optimized, photodynamic therapy enhanced, pH‐mediated multifunctional nanozyme catalysis effect for targeted bacterial eradication and tissue regeneration, thus provides a potentially versatile antibiotic‐free therapeutic strategy for managing recalcitrant infections in impaired wounds.

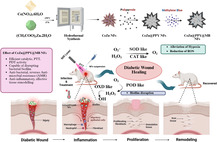

## 1 Introduction

Antibiotics are the predominant pharmaceutical agents utilized to combat bacterial infections and are extensively employed in antimicrobial treatments. Nevertheless, the improper utilization of antibiotics has resulted in the rise of multidrug‐resistant (MDR) strains, like methicillin‐resistant *Staphylococcus aureus* (MRSA), which significantly hampers the effectiveness of medicinal therapies. Hence, it is imperative to develop efficacious antibacterial drugs and techniques that can combat bacterial infection and facilitate wound healing in diabetic patients while minimizing the risk of drug resistance.^[^ ^1^ ^]^ The process of wound healing incorporates four prominent phases: hemostasis, inflammation, proliferation, and remodeling.^[^ ^2^ ^]^ Diabetic wound infections impose a substantial burden on both the healthcare delivery system and on individuals.^[^ ^3^ ^]^ To prevent further complications, the ulcers should be effectively identified and treated. Chronicity and complications can be attributed to the biofilm formation tendency in these wounds.^[^ ^4^ ^]^ Additionally, the presence of biofilm serves as a significant limitation to effective treatment. Biofilm formation is crucial in diabetic wound pathology, as it establishes robust bacterial communities within an extracellular polymeric substance (EPS) matrix, which protects bacteria from host immune responses and standard antimicrobial treatments.^[^ ^5^ ^]^ The biofilm matrix significantly restricts antibiotic penetration and effectiveness, resulting in persistent infections, chronic inflammation, and prolonged wound healing.^[^ ^4^ ^]^ This phenomenon causes the failure of traditional treatments that rely on systemic or topical antibiotics and surgical debridement. Mechanical debridement is necessary but frequently inadequate in fully eliminating biofilms, necessitating repeated interventions.^[^ ^6^ ^]^

Emerging therapeutic technologies provide notable benefits by more effectively targeting biofilm structure and bacterial viability.^[^ ^7^ ^]^ Given the recalcitrance of biofilm‐associated infections, emerging strategies such as photothermal therapy (PTT) aim to disrupt biofilm matrices while minimizing off‐target effects.^[^ ^8^ ^]^ PTT employs light‐responsive materials to produce localized heat, effectively disrupting biofilm matrices and eliminating sessile bacteria.^[^ ^9^ ^]^ This method allows for deeper penetration and improved antimicrobial efficacy. Photothermal and photodynamic therapies, when combined, demonstrate synergistic effects in the eradication of biofilms formed by resistant pathogens, including *Pseudomonas aeruginosa* and *Staphylococcus aureus* (SA).^[^ ^10^ ^]^ Advanced strategies encompass enzymatic degradation of EPS, disruption of quorum sensing, and innovative delivery systems such as microneedle bandages that enhance the bioavailability of therapeutic agents directly within biofilms and wound tissue.^[^ ^11^ ^]^ These multifaceted approaches eradicate biofilms and modulate the wound microenvironment by reducing oxidative stress and inflammation, and promoting tissue regeneration and angiogenesis essential for healing.

Photodynamic therapy (PDT) and PTT have attracted attention as antibiotic‐free treatments due to their qualities of noninvasiveness, minimal toxicity, and the ability for remote control. Near‐infrared (NIR) light can penetrate tissue to greater depths and shows less phototoxicity than ultraviolet (UV) light.^[^ ^12^ ^]^ Photosensitizers facilitate the transformation of endogenous oxygen into reactive oxygen species (ROS) to destroy cancerous cells and microorganisms in PDT. PDT & therapeutic impact are limited by ROS with a brief duration of efficacy and a limited spectrum of activity. When NIR lasers are used during PTT, local hyperthermia is produced by photothermal agents through the conversion of light energy to heat energy. However, a combination of photothermal strategies is required to achieve optimum antibacterial effects at moderate temperatures.^[^ ^13^ ^]^ Temperatures reaching 50 °C have the potential to inflict damage on DNA and result in the denaturation of intracellular proteins/enzymes, producing irreversible tumor ablation or bacteria‐killing.^[^ ^14^ ^]^ A novel antibacterial treatment method has gained recognition in recent years: the combination of nanotechnology with metal antibacterial activity. Metal nanoparticles have distinct properties that enable them to efficiently hinder the proliferation of drug‐resistant bacterial strains. They can serve as potent antimicrobial agents by employing diverse mechanisms that are not present in traditional therapeutic methods.^[^ ^15^ ^]^ Zinc oxide (ZnO) is a biocompatible semiconductor metal oxide that possesses antibacterial, anti‐infective, immunomodulatory, and tissue regenerative capabilities. In addition, Zn is crucial for the functioning of 300 catalytic enzymes that influence cellular differentiation and proliferation, along with the modulation of wound healing processes.^[^ ^16^ ^]^ The utilization of ceria oxide nanoparticles (CNPs) in the management of diabetes presents notable benefits.^[^ ^17^ ^]^ Biomedical research makes extensive use of CNPs owing to their remarkable antioxidant characteristics and elevated stability. By reversible cycling between the ionic states Ce^3+^ and Ce^4+^, CNPs are capable of sustaining the desired oxidation state and acquiring prolonged antioxidant properties.^[^ ^18^ ^]^ The skin, being a vital organ, serves the purpose of safeguarding the body and maintaining surface homeostasis. The destruction of the skin will result in the need for challenging and complicated healing methods for wounds. Wound healing involves inhibiting the growth of microorganisms while promoting the movement of cells and the rebuilding of tissue.^[^ ^19^ ^]^ During diabetic wound healing, multiple unique cell types are regulated at different stages of the biological process.^[^ ^20^ ^]^ Impaired blood flow and diabetic neuropathy contribute to delayed wound healing, which can result in severe consequences, including infections and potential amputation. Essential components of treatment include proper wound care, blood sugar level control, off‐loading, infection control, debridement, and wound closure. Timely identification and intervention of diabetic wounds are essential to avert additional problems.^[^ ^21^ ^]^ Research indicates that in diabetic patients, immune cells exhibit diminished functionality in responding to infections as a result of prolonged elevated glucose levels.^[^ ^22^, ^23^ ^]^ Decompensated hyperglycemia alters the morphology and mean activity of critical effector immune cells, such as neutrophils and macrophages, subsequently inhibiting their chemotaxis, phagocytic capabilities, and microbial killing efficiency.^[^ ^24^ ^]^ The reduction in host resistance among diabetics renders diabetic wounds more vulnerable to bacterial colonization and chronic infection.^[^ ^22^ ^]^ Moreover, a hyperglycemic microenvironment caused by diabetes leads to sustained and chronic inflammation in diabetic wounds.^[^ ^25^ ^]^ Tumor necrosis factor‐α (TNF‐α), interleukin‐1β (IL‐1β), and IL‐6 (IL‐6), along with various other pro‐inflammatory cytokines, are more prevalent in the diabetic state, which is characterized by constant low‐level inflammation and an imbalance between M1 and M2 cells. This persistent inflammation impairs the body's recovery capacity after an injury.^[^ ^26^ ^]^ The accumulation of M1 macrophages and the impairment of host immunity contribute to biofilm development, as well as exacerbating pro‐inflammatory responses in the wound, leading to further deterioration of the wound microenvironment.^[^ ^25^ ^]^ A major obstacle in the treatment of diabetic wounds is that these wounds have distinct vascular features, such as impaired angiogenesis and endothelial dysfunction.^[^ ^5^ ^]^ Angiogenesis does not satisfy the hypoxia and nutrient demand of the wound site.^[^ ^22^ ^]^ Because oxygen is a critical component to cellular repair and immune cell processes, the chronic hypoxic environment impairs wound healing.^[^ ^22^ ^]^ Poor perfusion and incredible vascular insufficiency at the wound site restrict oxygen delivery, thereby delaying host cellular processes and increasing the time that immune cells access the infected site, thus increasing the potential for infection. Decreased blood flow causes metabolic waste products to accumulate and creates an acidic or alkaline environment, which can inhibit healing and induce bacterial growth and biofilm formation.^[^ ^26^ ^]^ Through a vicious cycle between hyperglycemia‐induced immune dysfunction and vascular insufficiency, the diabetic wound is established as detrimental. The inability of the immune system to respond facilitates bacterial expansion, which often establishes microcolonies that mature into biofilms, structures known for exhibiting increased resistance to antibiotics and host defenses.^[^ ^24^ ^]^ Chronic infections cause ongoing inflammation and destruction of tissues, and underlying vascular disease prevents the delivery of oxygen and nutrients required for tissue repair. This chronic state of persistent infection, inflammation, and impaired tissue perfusion greatly disrupts all stages of wound healing and produces the prolonged nonhealing wounds that are the hallmark of diabetes complications.^[^ ^19^ ^]^

In PTT, polypyrrole nanoparticles, a novel class of inexpensive, exceptionally reliable, and compatible photothermal agents, have garnered considerable interest. These materials have exhibited broad coverage capabilities, functioning effectively across the complete UV–visible spectrum, including the NIR region. The limited wavelength coverage, namely in the UV‐light area, may be attributed to the π–π* transition occurring at the backbone of the conjugated polypyrrole. Conversely, the bipolaronic polypyrrole exhibits a broad spectrum spanning from the visible to the NIR. In addition, polypyrrole nanoparticles can withstand exposure to extreme circumstances and remain stable in alkaline or acidic environments. Polypyrrole nanoparticles are very suitable for photothermal sterilizing across a significant section of the solar spectrum due to their aforementioned features.^[^ ^27^ ^]^ Methylene blue (MB), a phenothiazinium photosensitizer, exhibits significant potential in PDT due to its high quantum yield of singlet oxygen (^1^O_2_) generation when excited within the therapeutic window of 600–900 nm, coupled with its minimal toxicity.^[^ ^28^ ^]^ We performed the synergistic application of PTT and catalytic activity based on bimetallic hybrid ceria‐zinc nanoflowers to treat MRSA infection in diabetes‐related wounds. For the first time, bimetallic hybrid nanozymes, ceria, and zinc nanozymes incorporated in the form of nanoflowers, coated with polypyrrole, and loaded with MB have been used to treat MRSA infection in diabetic wounds. While building upon well‐established components like ceria‐zinc nanoflowers, polypyrrole, and MB, it presents a distinctive and significant advancement in diabetic wound healing through the innovative synthesis and multifunctional integration of these materials into a bimetallic hybrid nanoflower nanozyme coated with polypyrrole and loaded with MB. Unlike prior works that focus on individual or dual‐function systems, our study uniquely combines nanozyme catalytic activity, PTT, and PDT into a single, synergistic platform that precisely modulates ROS generation and scavenging in response to the diabetic wound microenvironment and NIR irradiation. This conjunction resulted in superior bacterial biofilm disruption, enhanced antibacterial action against MDR bacteria, and promoted angiogenesis and tissue regeneration, validated extensively through in vitro and in vivo models. Additionally, mechanistic insights into pH‐responsive nanozyme activity, laser‐activated synergistic ROS production, and dynamic interplay between photothermal and photocatalytic functions provide new conceptual understanding beyond incremental material combination. The combined strategy's goal is to surpass the shortcomings of traditional treatments, which are frequently ineffective when it comes to MRSA and affect impaired healing conditions surrounding diabetic wounds. We posit that the NIR on‐demand multifunctional bimetallic hybrid nanoflower nanozymes will facilitate enhanced bactericidal efficacy and accelerate the wound healing process in diabetic models.

## 2 Results and Discussion

### 2.1 Preparation and Characterization of Ceria‐Zinc Nanoflower

**Figure 1:**
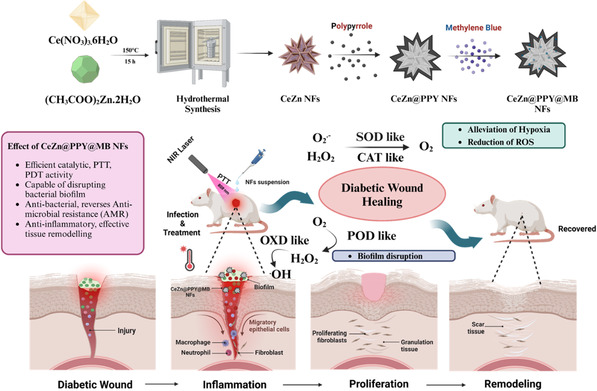
Schematic illustration of the development of CeZn@PPY@MB NFs for enhanced phototunable therapy against MRSA‐infected diabetic wounds. The novel formulation employs the synergistic photothermal and catalytic properties of polypyrrole (PPY)‐coated, MB‐loaded ceria‐zinc nanoflowers to effectively target and eliminate MRSA infections, which are prevalent complications in diabetic wounds. Nanoflower‐mediated photothermal therapy demonstrates potential in the treatment of MRSA‐infected diabetic wounds.

A new nanocomposite consisting of the dual metal‐containing nanoflower (ceria and zinc), which is coated by the photothermal agent PPY, loaded with the photosensitizer MB, has been designed. This design is expected to simultaneously improve photothermally accelerated, catalytically regulated antibacterial, anti‐inflammatory, and repair abilities to effectively heal MRSA‐infected diabetic wounds (**Figure** **1**). **Table** **1** represents the particle size, zeta potential, and % MB loading for equimolar ceria‐zinc nanoflowers. According to the table, the nanoflower CeZn@1.8PPY NFs exhibited suitable particle size, zeta potential, and PDI values of 234.2 ± 1.69 nm, 32.2 ± 0.31 mV, and 0.312, respectively (Figure S1F,G, Supporting Information). Upon dissociation from the CeZn@1.8PPY@MB NFs, they exhibited a maximum % MB loading of 7.86 ± 0.11, based on the MB‐absorbance peak in methanol at 654 nm, without and with NIR (808 nm) irradiation (Figure S1D,E, Supporting Information). So, CeZn@1.8PPY@MB NFs were selected for further studies for investigation.

**Table 1:** Physicochemical characterization of uncoated CeZn NFs, CeZn@0.6PPY NFs, CeZn@1.2PPY NFs, and CeZn@1.8PPY NFs.

Sr. No.	Ratio by wt. (Ce: Zn)	% PPY coating	% MB loading	Average particle diameter [d. nm]	PDI	Zeta potential [mV]
1.	50:50	Uncoated	1.44 ± 0.52	182.3 ± 2.47	0.153	15.4 ± 0.65
2.	50:50	0.6	4.33 ± 0.37	202.1 ± 4.13	0.201	23.7 ± 0.48
3.	50:50	1.2	4.92 ± 0.25	220.6 ± 3.21	0.274	28.1 ± 1.04
4.	50:50	1.8	7.86 ± 0.11	234.2 ± 1.69	0.312	32.2 ± 0.31

The UV–vis absorption spectra of the MB doped to the CeZn@PPY@MB NFs clearly show a redshift and broadening of the MB absorption peak upon doping with polypyrrole (PPY), extending MB's effective excitation into the NIR region around 808 nm.

**Figure 2:**
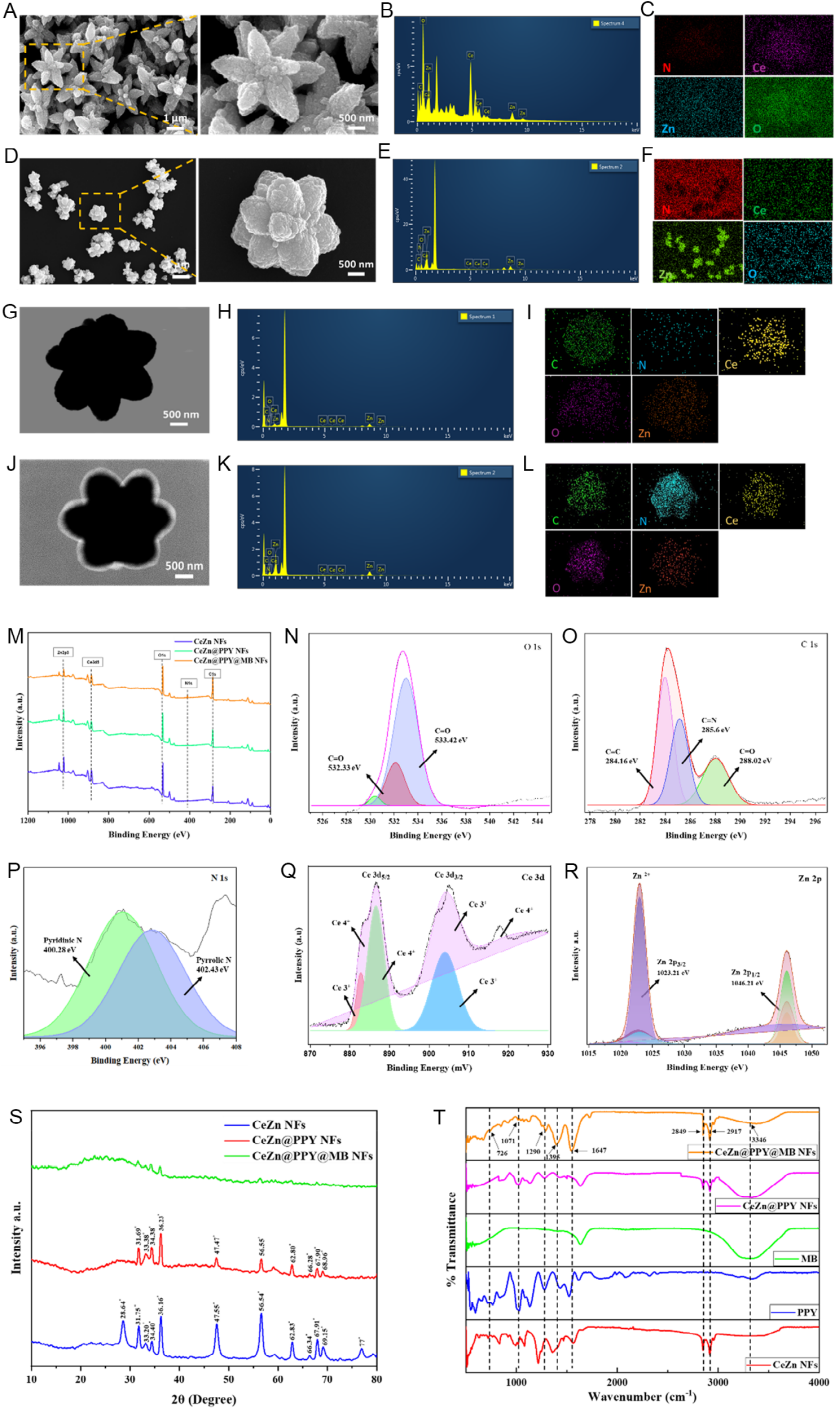
Morphology and physicochemical characterization of ceria‐zinc nanoflowers. A–C) SEM images, EDX, and elemental mappings of uncoated CeZn NFs. D–F) SEM images, EDX, and elemental mappings of CeZn@PPY@MB NFs. G–I) STEM images, EDX, and elemental mappings of uncoated CeZn NFs. J–L) STEM images, EDX, and elemental mappings of CeZn@PPY@MB NFs. M) XPS survey of CeZn NFs, CeZn@PPY NFs, and CeZn@PPY@MB NFs. High‐resolution XPS spectra of N) O 1s, O) C 1s, P) N 1s, Q) Ce 3 d, and R) Zn 2p. S) XRD patterns of CeZn NFs, CeZn@PPY NFs, and CeZn@PPY@MB NFs. T) FTIR spectra of free MB, PPY, CeZn NFs, CeZn@PPY NFs, and CeZn@PPY@MB NFs.

The scanning electron microscopy (SEM) analysis was performed to examine the morphology of uncoated CeZn NFs and MB‐loaded, PPY‐coated CeZn@PPY@MB NFs. From **Figure** **2**A,B, a morphology similar to that of a water lily is revealed, showcasing petal‐like elements of ceria‐doped zinc oxide petals radiating radially from a central core. This structural feature is expected to provide effective PTT along with greater therapeutic agent loading. This behavior matches similar findings by Chopan and Chishti's work with polypyrrole‐based nanocomposites.^[^ ^29^ ^]^ The conducted energy dispersion X‐ray spectroscopy, along with the elemental mapping, clarifies the basic understanding of the elemental composition and distribution (Figure 2B,C,E,F). Figure 2G,J represent the scanning transmission electron microscopy (STEM) images of uncoated CeZn NFs and coated CeZn@PPY@MB NFs. Heavy metal components, Ce and Zn, containing high electron density, cause the formation of a dark appearance, while being coated with PPY, with lower electron density, allows a greater proportion of the electron beam to pass through, revealing a white lining around the dark CeZn NFs.^[^ ^30^ ^]^ Figure 2H,I,K,L show the typical peaks corresponding to those of cerium (Ce), zinc (Zn), oxygen (O), carbon (C), and nitrogen (N), signifying their presence on the surfaces of the nanocomposites. An extensive coating and loading technique can be indicated from the revealed homogenous elemental distribution. A similar study by Shen et al. also demonstrates that a uniform element distribution is necessary for the desired properties of the nanocomposites.^[^ ^31^ ^]^ The PPY coating can be visualized in the form of a homogenous coating on the surface of the nanoflowers (Figure 2J). The presence of the homogenous PPY coating on the nanocomposites signifies the electrical conduction phenomenon due to the π–π* transitions along the PPY‐structural backbone, thus providing an efficient photothermal activity under NIR 808 nm irradiation. Together with the elemental mapping covered above, the SEM images of the water lily form confirm the successful manufacture of the nanocomposites with a homogeneous distribution of matching components.

The overall X‐ray photoelectron spectroscopy (XPS) spectra of CeZn NFs, CeZn@PPY NFs, and CeZn@PPY@MB NFs are represented in Figure 2M. The O 1s peaks that were resolved into their individual components (Figure 2N) confirm that different types of surface oxygen exist on the bare CeZn NFs, at binding energies of 533.33 and 536.44 eV, respectively. The presence of PPY coating over CeZn@PPY NFs and CeZn@PPY@MB NFs is confirmed through the spectra of C 1s (Figure 2O) and N 1s (Figure 2P). The binding energy of the corresponding integrated peaks of Ce^+4^ is shown in the deconvoluted Ce 3d peaks in Figure 2Q are 884.09, 889.53, 898.21, 900.62, 904.37, and 917.26 eV, respectively.^[^ ^32^ ^]^ The Zn 2p peak of pure ZnO, as well as ZnO doped with Ce, was deconvoluted and fitted with two Gaussian peaks that described Zn 2p1/2 (1046.21 eV) and Zn 2p3/2 (1023.21 eV), as presented in Figure 2R. These energy levels are directly associated with the Zn^+2^ ions and oxygen molecules that have chemically bonded to the surface (hydroxyl groups). The extra peak at 529.32 nm is due to the formation of Ce^+3^ ions. This results in oxygen ionic bond formation in the ZnO lattice through Ce insertion.

The crystallographic structure and phase composition of the CeZn@PPY@MB NFs were elucidated through an X‐ray diffraction (XRD) pattern analysis. In the following analysis, CeZn NFs showed peaks at 2*θ* of 28.64, 31.75, 33.20, 34.40, 36.16, 47.55, 56.54, 62.83, 66.34, 67.91, 69.15, and 77 in CeZn@PPY NFs, peaks were evident at 2*θ* of 31.69, 33.38, 34.38, 36.23, 47.47, 56.55, 62.80, 66.28, 67.90, and 68.96 and the characteristic peaks of CeZn disappeared in CeZn@PPY@MB NFs, revealing the drug in the amorphous form (Figure 2S). XRD peaks were indexed following the standard powder diffraction file for CeO_2_ (ICDD PDF #34‐0394) and ZnO (ICDD PDF #36‐1451), respectively.^[^ ^33^, ^34^ ^]^ The acquired peaks can be compared with the standard wurtzite structure of ZnO, displaying the presence of ZnO, which is found at the 2*θ* values associated with the crystalline structure.^[^ ^16^ ^]^ The intensities of the peaks of ZnO can provide information about the preferential orientation of the ZnO crystals in the nanoflowers and confirm the presence of a hexagonal wurtzite structure.^[^ ^35^ ^]^ Because PPY is in amorphous form, PPY itself would not show any sharp diffraction peaks to indicate its presence. A wide and diffuse background in the corresponding XRD pattern confirms the existence of the polymer coating. The diffuse XRD patterns confirm the composite nature and exhibit peaks from different components, as discussed by Das et al.^[^ ^36^ ^]^ In addition, correlating XRD patterns with different analytical experimentations, like SEM, which gives morphological information, helps in deciphering the crystalline structure and general characteristics of CeZn@PPY@MB NFs.

The Fourier transform infrared spectroscopy (FTIR) spectra of CeZn NFs showed the absorption peak at 726 cm^−1^, indicating the occurrence of oxide stretching. The spectral bands identified at 3360 cm^−1^ are indicative of the vibrational modes associated with O—H bonds. The FTIR spectra of CeZn@PPY NFs showed the absorption peaks at 1071 and 3346 cm^−1^, which are attributed to the vibrational mode of both C—H and O—H functional groups. The absorption bands found at 1534 and 1407 cm^−1^ in CeZn@PPY NFs are related to the basic stretching vibration corresponding to the pyrrole rings.^[^ ^29^ ^]^ Similarly, the band at 1280 cm^−1^ is attributed to the C—N stretching vibration. The additional peaks seen at 1128, 1071, 929, and 847 cm^−1^ corresponded to the in‐plane, out‐of‐plane, C—H, and N—H bending vibrations, respectively, as shown in Figure 2T. The FTIR spectra of CeZn@PPY@MB NFs exhibited an absorption peak at 1647 cm^−1,^ which can be ascribed to the C=O stretching vibration. The absorption band at 3346 cm^−1^ is attributed to the O—H stretching vibration. All of these peaks indicate the effective synthesis of CeZn@PPY@MB NFs.

Thus, the developed CeZn NFs, CeZn@PPY NFs, and CeZn@PPY@MB NFs hold great potential for diabetic wound healing. They possess the potential for extended photothermally boosted catalytic activity for efficient antibacterial, antibiofilm, and tissue regeneration‐promoting activity.

### 2.2 Photothermal Performance of Ceria‐Zinc Nanoflowers

**Figure 3:**
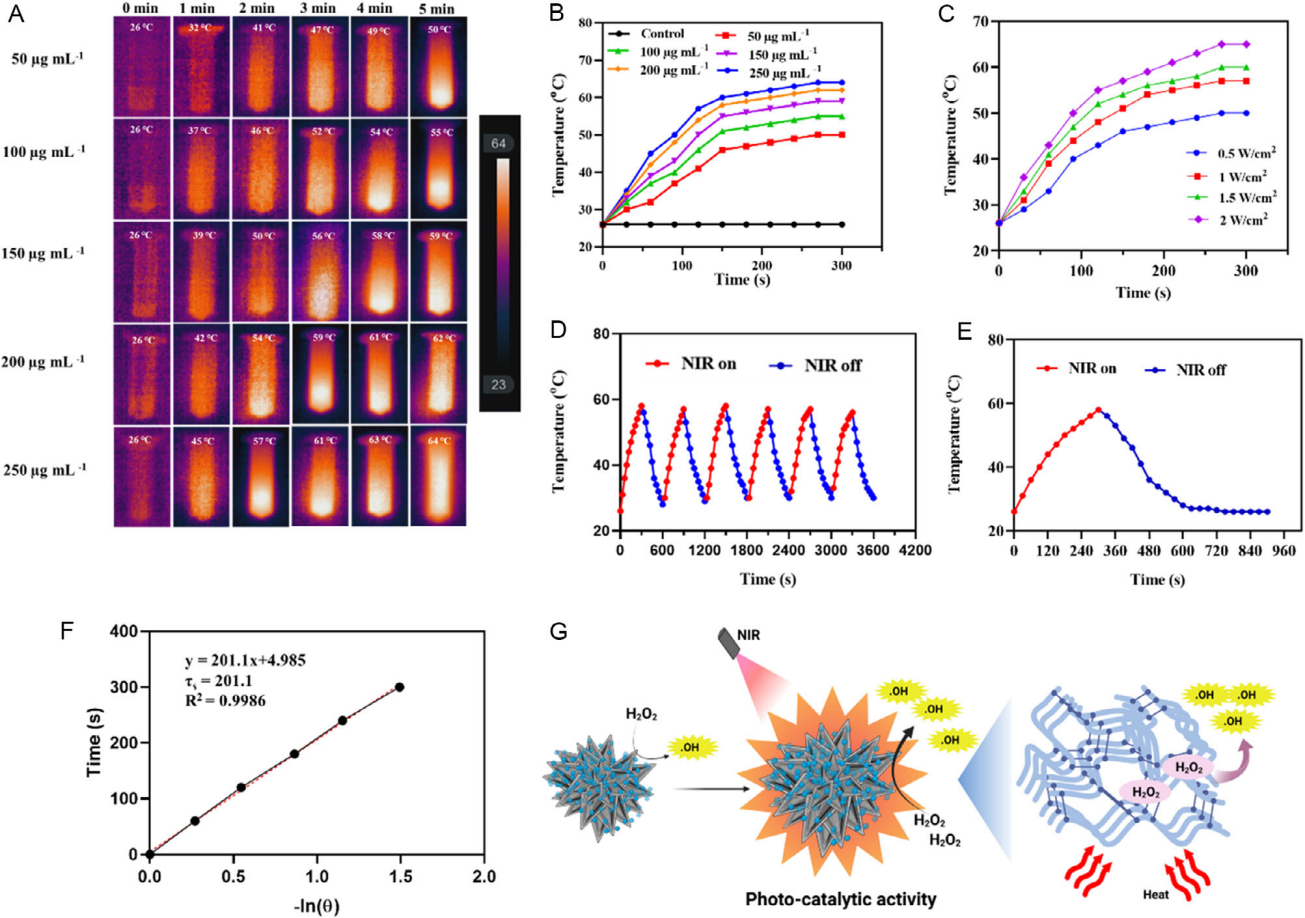
Photothermal performance of CeZn@PPY@MB NFs under NIR (808 nm) irradiation. A,B) Thermal images and temperature increase curves of CeZn@PPY@MB NFs of varying concentrations with NIR (808 nm) irradiation (1 W cm^−2^, 5 min). C) Photothermal heating curves of CeZn@PPY@MB NFs dispersion with varying intensities of irradiation (100 μg mL^−1^, PBS 7.4, 5 min). D) Temperature change curves of CeZn@PPY@MB NFs dispersion during NIR (808 nm) on/off cycles (1 W cm^−2^). E) Photothermal stability curve of CeZn@PPY@MB NFs dispersion within single on/off laser irradiation (PBS 7.4, 1 W cm^−2^). F) Graph showing the variation of linear time against ‐ln *θ* obtained during the cooling period after laser termination. G) A schematic representation of laser‐enhanced PDT due to CeZn@PPY@MB NFs.

A straightforward hydrothermal technique using the unique properties of cerium oxide and zinc oxide developed a reactive and biocompatible platform, thereby synthesizing bimetallic hybrid ceria‐zinc nanoflowers. Following its coating with a thin layer of polypyrrole (PPY), a conductive polymer that was identified for its exceptional photothermal conversion efficiency under NIR irradiation.^[^ ^37^ ^]^ Originally identified for its PDT against bacterial populations, MB was then combined with a nanoflower structure to provide a synergistic improvement in its photothermal effectiveness at inactivating the bacterial population through the production of mild hyperthermia.^[^ ^38^, ^39^ ^]^ A UV–vis spectrum of the developed CeZn@PPY@MB nanoflowers was obtained (100 μg mL^−1^, PBS 7.4), suggesting good absorbance of light energy in the NIR region (808 nm). Using infrared thermal imaging, the photothermal conversion of the CeZn@PPY@MB NFs was evaluated (**Figure** **3**A). Concentration‐dependent photothermal properties were demonstrated, and it was observed that a 100 μg mL^−1^ concentrated solution reached a maximum temperature of 55 °C with 5 min of laser irradiation (1 W cm^−2^), as shown in Figure 3B. Under various intensities of NIR (808 nm), successively ranging from 0.5 to 2 W cm^−2^, an irradiation‐intensity‐dependent photothermal performance of the nanocomposite was evaluated for 100 μg mL^−1^ (Figure 3C), and a 1 W cm^−2^ laser intensity was confirmed to possess a notable capacity to generate localized heat and effectively inactivate MRSA or SA cells, following light exposure. Warming and cooling curves were obtained that helped to establish the quantitative photothermal efficiency (*η*). The photothermal effect of the CeZn@PPY@MB NFs (100 μg mL^−1^) was investigated via on‐off pulsing of an NIR laser (1 W cm^−2^). As observed, the temperature synchronously increased and decreased with the on/off switching of NIR (808 nm, 1 W cm^−2^) and was maintained for six cycles, which could demonstrate the proper photothermal stability of the CeZn@PPY@MB NFs (Figure 3D). Furthermore, a remarkable photothermal efficiency was maintained within a complete cycle of heating and cooling, confirming its photothermal stability (Figure 3E). Following the confirmation of photothermal stability, the half‐field time (*τ*_s_) was calculated to be 201.1 s, and the photothermal conversion efficiency (*η*) was calculated to be 61.492% (Figure 3F).

Figure 3G explains the laser‐enhanced PDT due to CeZn@PPY@MB NFs. The overall photothermal phenomenon of the CeZn@PPY@MB NFs can be attributed to the synergistic effects between its two main components: PPY and MB. PPY's photothermal effect is attributed to its conjugated polymer backbone. On application of light, particularly in the NIR spectrum, electrons are stimulated on the PPY structure. The excited electron then relaxes back into the ground state through nonradiative pathways, and the absorbed energy is emitted as heat. PPY typically absorbs light over a wide range, but its NIR absorption is of significance in biomedical applications since NIR penetrates deeper into biological tissues.^[^ ^10^ ^]^ There are no inherent photothermal characteristics of MB compared to materials such as PPY. MB in phototherapy works primarily as a photodynamic agent, as opposed to a photothermal agent. MB has potential synergistic therapeutic effects when combined with photothermal agents.^[^ ^40^ ^]^ Photothermal nanoparticles have been used in conjunction with MB, and we have researched this type of combination. In this system, the photothermal agent, polypyrrole or gold nanoparticles, receives light and produces heat, and the MB generates ROS through photodynamic action. Such a combined approach might enhance therapeutic outcomes. Yu et al. discussed gold nanoparticles conjugated to MB for targeting and PDT, confirming that this system was proven to work effectively.^[^ ^41^ ^]^ The above findings justify the significant photothermal activity of the developed CeZn@PPY@MB NFs, emphasizing their potential antibacterial effectiveness against resistant strains, including MRSA.

### 2.3 Synergistic Multienzyme Activities of Ceria‐Zinc Nanoflower

**Figure 4:**
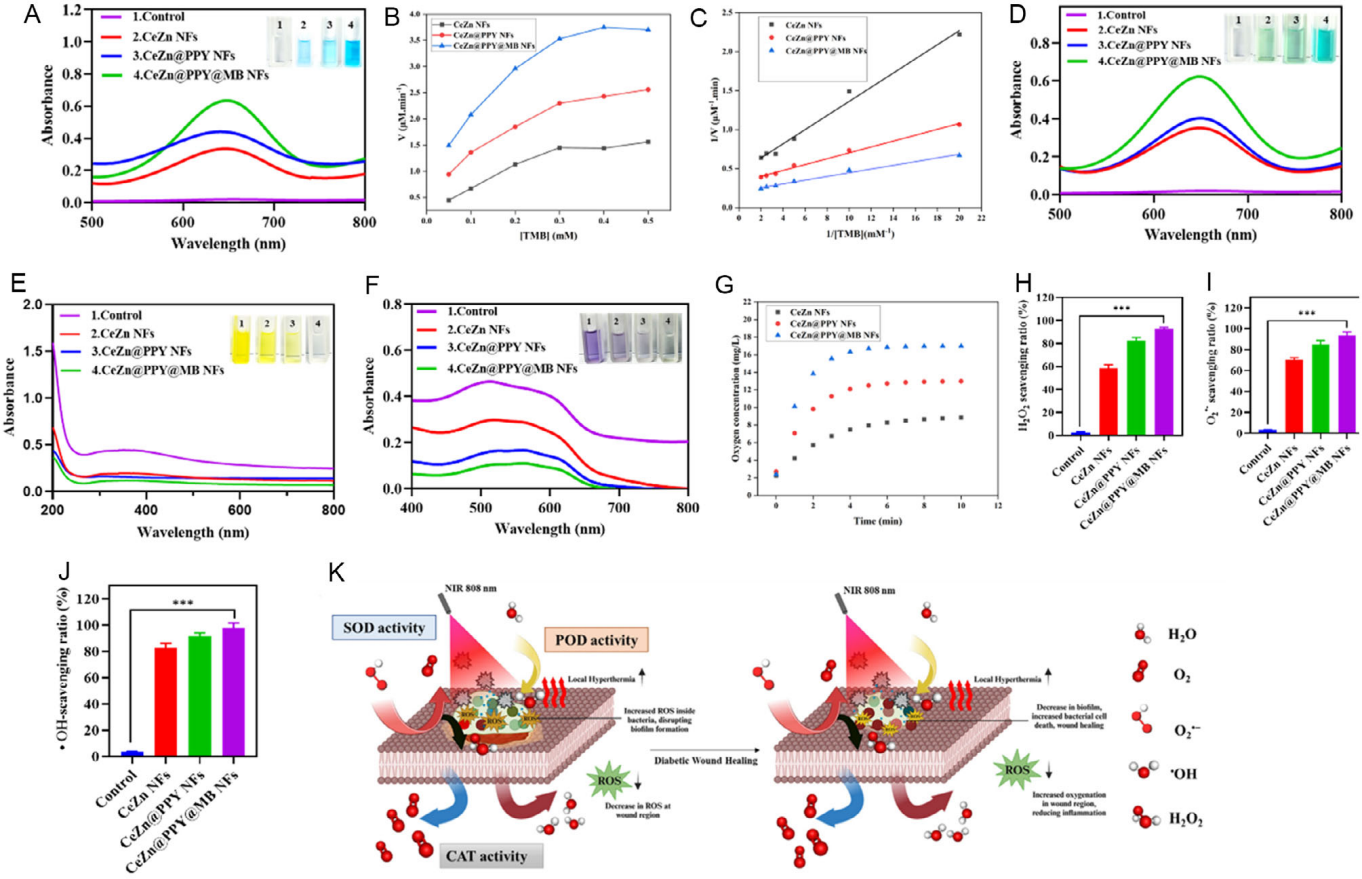
Synergistic multienzyme activities of ceria‐zinc nanoflower after NIR (808 nm) irradiation (1 W cm^−2^, 5 min). A) UV–vis spectra showing the POD‐like activity due to CeZn NFs, CeZN@PPY NFs, and CeZn@PPY@MB NFs in the presence of acidic pH 4.5. B,C) Steady‐state POD‐like enzyme kinetics of the nanoflowers. D) UV–vis spectra showing OXD‐like activity of the nanoflowers. E,F) CAT‐ and SOD‐like activities of CeZn NFs, CeZn@PPY NFs, and CeZn@PPY@MB NFs in the presence of PBS 7.4 (*n* = 5). G) Oxygen generation from the supplied H_2_O_2_ due to CAT‐like activity by the nanoflowers. H) H_2_O_2_ scavenging ratios, I) singlet oxygen scavenging ratios and J) hydroxyl radical scavenging ratios of CeZn NFs, CeZn@PPY NFs, and CeZn@PPY@MB NFs, versus PBS 7.4 as a control (*n* = 5). K) A schematic representation of the laser‐enhanced photothermal, photocatalytic, and photodynamic effects of CeZn@PPY@MB NFs. All data represented as mean ± SD (*p < 0.05, **p < 0.01, ***p < 0.001).

The dysfunction of enzymatic activity in diabetic wounds impairs the antioxidant homeostasis required for healing.^[^ ^42^ ^]^ These PPY‐coated, MB‐loaded bimetallic hybrid ceria‐zinc nanoflowers exhibited multiple enzyme‐like functions in response to the variation of pH and in the presence of NIR irradiation. The synthesized nanoflowers imitate peroxidase‐like (POD) and oxidase‐like (OXD) enzymatic activities at a lower pH (**Figure** **4**A,D). CeZn@PPY@MB NFs demonstrated strong POD‐like activity, which mediated the oxidation of chromogenic substrate 3,3′,5,5′‐tetramethylbenzidine (TMB) with H_2_O_2_.^[^ ^24^ ^]^ The activity was confirmed by a distinct color transformation to the TMB and H_2_O_2_ solution in a weakly acidic environment. The absorbance change at 652 nm was used to quantify the POD‐like activity. This generated free hydroxyl (·OH) radical extensively through POD‐like activity, to induce damage to the bacterial cells, increase intracellular ROS, and exert their bactericidal effect. An extensive evaluation of the POD‐like activity due to the nanoflowers was performed through the assessment of maximum enzymatic reaction rates (*V*_max_) and the kinetic constants (*K*_m_). According to Figure 4B,C, and **Table** **2**, the CeZn@PPY@MB NFs showed the maximum *V*_max_ value of 4.731 μM min^−1^ and minimum *K*_m_ value of 0.112 mM compared to the CeZn NFs. The enhancement was visualized on the application of NIR (808 nm) laser irradiation (1 W cm^−2^, 5 min). The photoenhanced catalase (CAT) (Figure 4E) and superoxide dismutase (SOD) (Figure 4F) like activities of the nanoflowers were also visualized on receiving NIR (808 nm) laser irradiation (1 W cm^−2^, 5 min). The capability to decompose H_2_O_2_ into water and oxygen due to the CAT‐like activity of the nanoflowers was determined, where CeZn@PPY@MB NFs showed the maximum concentration of oxygen production over a span of 10 min, where the production rate achieved a steady state after 5 min (Figure 4G). The CeZn@PPY@MB NFs dispersion had the lowest intensity absorbance peak compared to all other nanoflower dispersions and the PBS control when combined with laser irradiation for 5 min. Furthermore, it exhibited the strongest ability to scavenge the externally supplied H_2_O_2_ (Figure 4H), among the other two nanoflowers, during the quantification. CeZn@PPY@MB NFs did exhibit a potent SOD‐like activity, thus having an inherent capacity to quench $O_{2^{\cdot -}}$radicals (Figure 4I). The activity was assayed with a standard assay of inhibition of $O_{2^{\cdot -}}$ induced reduction of chromogenic agent NBT, with a riboflavin and methionine combination being a positive control. Combined with the significantly high SOD‐like activity of CeZn@PPY@MB NFs, which is in accordance with the results described by Ju et al.,^[^ ^43^ ^]^ scavenges the generated $O_{2^{\cdot -}}$ radical from the present O_2_, contributing to their enhanced capacity to regulate ROS levels and protect against oxidative damage in the wound environment.

**Table 2:** Steady‐state POD‐like enzyme kinetics of the nanoflowers.

Parameters	CeZn NFs	CeZn@PPY NFs	CeZn@PPY@MB NFs
*V*_max_ [μM min^−1^]	2.190	3.055	4.731
*K*_m_ [mM]	0.199	0.115	0.112

Moreover, the CeZn@PPY@MB NFs showed extensive OH‐radical depleting activity compared to the other nanoflower formulations (Figure 4J). The promising multienzyme activities suggest that the critically tailored CeZn@PPY@MB NFs possess the potential for therapeutic applications, such as diabetic wound healing. The pH‐dependent catalytic activities of CeZn@PPY@MB NFs were confirmed through the visualization of enhancement of dissolved oxygen production and $O_{2^{\cdot -}}$ radical scavenging activities with the increase in pH from 5 to 7.4 (Figure S9, Supporting Information).

The catalytic performance of nanozymes is influenced by pH levels.^[^ ^44^ ^]^ The POD‐like activity in CeZn@PPY@MB NFs is optimized in acidic to mildly acidic conditions (pH 4–6), which is advantageous in the context of early wound infection and inflammation. Robust POD activity facilitates the conversion of H_2_O_2_ into hydroxyl radicals (·OH), thereby enhancing bacterial clearance and disrupting biofilms. CAT and SOD activities exhibit optimal performance in mildly acidic to alkaline pH ranges (6–7.4), which corresponds with the later stages of the healing process. Catalase decomposes excess H_2_O_2_, mitigating oxidative damage and facilitating tissue oxygenation for angiogenesis and repair. SOD scavenges superoxide radicals, decreasing oxidative stress and promoting tissue regeneration.^[^ ^45^ ^]^ Thus, CeZn@PPY@MB NFs hold the potential to provide phase‐specific enzyme‐mimicking activity, adjusting the oxidative environment and facilitating wound healing in accordance with the wound stage and pH. In addition, these multienzyme activities provide synergistic functions toward ROS modulation, antibacterial activity, and wound healing representations, as represented in Figure 4K. The catalytic abilities described in the work of Yang et al., share the commonality of integrating cerium and zinc oxide nanozymes for multi‐enzyme mimetic activity but differ significantly in catalytic scope, pH responsiveness, and therapeutic integration. The ceria/zinc nanocomposite described in their work exhibits strong POD‐like activity at acidic pH, and CAT‐ and SOD‐like activities at neutral pH, enabling ROS modulation via both ROS generation (for antibacterial action) and ROS scavenging (to alleviate oxidative stress), with the release of Ce^3+^/Ce^4+^ and Zn^2+^ ions contributing to anti‐inflammatory and pro‐angiogenic effects; however, its catalytic action is not coupled with external physical triggers. In contrast, the CeZn@PPY@MB NFs not only display pronounced multi‐enzyme mimetic activity (POD, CAT, SOD, and additional OXD‐like activity) but also exploit NIR‐triggered photothermal (PPY) and photodynamic (MB) synergy to greatly enhance catalytic output, particularly POD‐like ·OH generation in acidic, infected wound microenvironments, while maintaining high CAT and SOD activity in neutral/healing phases. This photothermally and photosensitizer‐augmented catalysis, combined with stage‐specific pH responsiveness, yields stronger antibacterial, antibiofilm, and angiogenesis‐promoting effects than the enzymatic activity alone in ZCO‐HA, making the CeZn@PPY@MB NFs a more dynamically controllable and multi‐modal catalytic platform for diabetic wound healing.^[^ ^46^ ^]^ The photocatalytic aspects of CeZn@PPY@MB NFs are comparable to those of the CCF TACs@NVs system developed by Li et al., meant for tumor cell killing. The photothermal effect is driven by multi‐metal single‐atom synergy, further stabilized by cell‐membrane coatings, enabling deep tissue heating and enhanced catalytic therapy. In contrast, the CeZn@PPY@MB NFs combine PPY's high NIR absorbance and thermal stability with MB's photosensitizer role, generating robust local hyperthermia under 808 nm NIR irradiation that efficiently disrupts biofilms, boosts nanozyme catalytic (POD) activity, and accelerates wound healing.^[^ ^47^ ^]^ Similarly, Liu et al. have explored the pH‐responsive OH‐radical generation ability through their FeMOF‐based nanozyme for efficient antitumor efficacy.^[^ ^48^ ^]^ The photocatalytic activity of the nanozyme is pH‐responsive, operating predominantly under the acidic conditions of infected wounds, while the photothermal effects from PPY are independent but complementary. This nuanced interplay between photothermal conversion and catalytic ROS generation in CeZn@PPY@MB NFs establishes a versatile, multifunctional platform for precise diabetic wound therapy.

### 2.4 Antibacterial and Antibiofilm Efficacy of Ceria‐Zinc Nanoflowers

Diabetic wounds are a significant healthcare challenge, often leading to limb amputation and increased morbidity and mortality. These chronic wounds are characterized by persistent bacterial infections, which can form recalcitrant biofilms that resist traditional antibiotic treatments.^[^ ^49^ ^]^ The bimetallic hybrid ceria‐zinc nanoflowers, namely CeZn NFs, CeZn@PPY NFs, and CeZn@PPY@MB NFs, have already been verified for their photothermal‐boosted catalytic properties and possess intrinsic catalytic properties. As they are combined with PTT‐inducing agent PPY, the nanoflowers were able to absorb light energy and produce mild hyperthermia, thus showing the potency for PTT. Combined with MB, a renowned photosensitizer, it can generate ROS, like singlet oxygen (^1^O_2_) and superoxide radicals ($O_{2^{\cdot -}}$). Being excited by the photothermal conversion capability of the ceria‐zinc nanoflowers, it was decided to explore their bactericidal potency by generating ROS. Xiao et al. mention the use of ceria in nanozymes for enhanced ROS generation and bacterial killing.^[^ ^22^ ^]^ Moreover, previous works have shown that in situ heat production can facilitate the generation of ROS, boosting the photodynamic action of MB; thus, the nanoflowers ought to show a synergistic bactericidal action on both the nonsensitive (MRSA) and sensitive strains of SA.^[^ ^50^ ^]^

The minimum inhibitory concentration (MIC) of free MB, CeZn NFs, CeZn@PPY NFs, and CeZn@PPY@MB NFs in the presence and absence of NIR laser irradiation was evaluated for both SA and MRSA (Figure S3, Supporting Information). It was observed that MIC values significantly reduced for uncoated CeZn NFs when they were modified using PPY and MB to form CeZn@PPY@MB NFs, plus NIR 808 nm (1 W cm^−2^) for 5 min, against both SA and MRSA, as shown in **Table** **3**.

**Table 3:** Minimum inhibitory concentration values of free MB, CeZn NFs, CeZn@PPY NFs, and CeZn@PPY@MB NFs against SA and MRSA, without and with NIR (808 nm, 1 W cm^−2^, 5 min).

	Formulations	SA [μg mL^−1^]	MRSA [μg mL^−1^]
Without NIR	MB	256	512
	CeZn NFs	128	256
	CeZn@PPY NFs	128	256
	CeZn@PPY@MB NFs	64	128
NIR	MB	128	256
	CeZn NFs	128	256
	CeZn@PPY NFs	64	128
	CeZn@PPY@MB NFs	16	32

**Figure 5:**
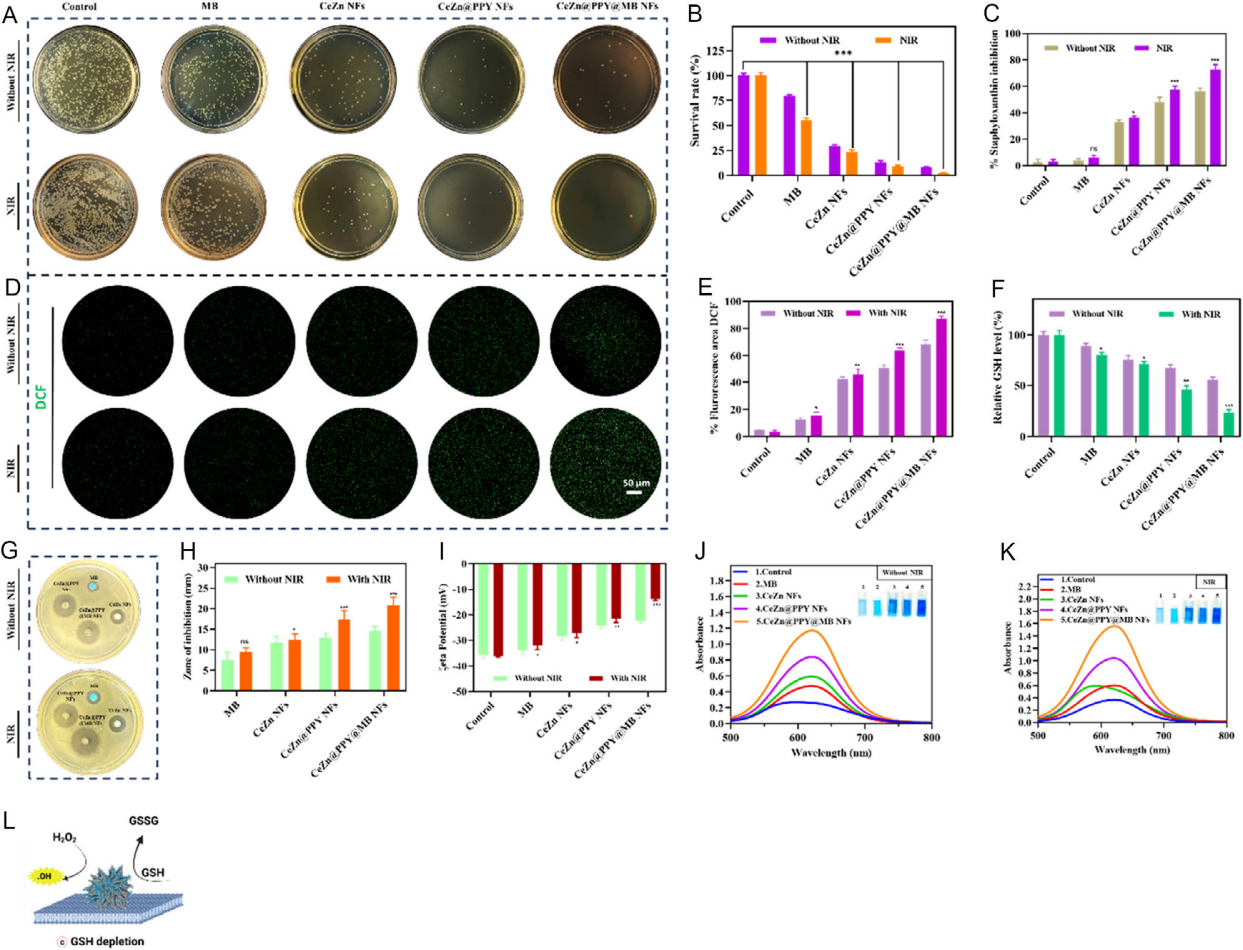
In vitro antibacterial effects of free MB, CeZn NFs, CeZn@PPY NFs, and CeZn@PPY@MB NFs treatments against methicillin‐resistant *Staphylococcus aureus* (MRSA) without and with NIR (808 nm, 1 W cm^−2^, 5 min) irradiation, compared to PBS (pH 7.4) treated control. A) Photographs of bacterial survival. B) Quantitative analysis of bacterial survival. C) Percentage inhibition of staphyloxanthin biosynthesis due to various nanoflower treatments. D) Images showing DCF fluorescent bacterial cell populations, visualized through the confocal microscope. Scale = 50 μm. E) Quantification of percentage DCF fluorescent area after treatment of bacterial populations with various formulations, with and without NIR irradiation. F) Analysis of intracellular GSH level after treatment of bacterial populations with various formulations, with and without NIR irradiation (*n* = 3). G) Photographs representing the diameter of the zone of inhibition around 6 mm discs infused with various nanoflower treatments, without and with NIR application. H) Quantitative analysis of the diameter of the zone of inhibition with various nanoflower treatments. I) Zeta potential of bacterial cultures treated with various nanoflower treatments. J,K) Protein leakage assay of bacterial cultures treated with various nanoflower treatments using Bradford reagent, without and with NIR irradiation, respectively (*n* = 3). L) A schematic illustration of enhanced biofilm penetration, bacterial membrane damage, and GSH depletion induced by CeZn@PPY@MB NFs by photothermal effect against MRSA. All data represented as mean ± SD (*p < 0.05, **p < 0.01, ***p < 0.001).

Coating of PPY exerts photothermal properties that enable the absorbance of light energy, cause moderate hyperthermia, and help MB to undergo photodynamic action, therefore increasing ROS formation. The development of ROS at this phase can significantly damage the bacterial cells, which increases the zone of inhibition. Zhao et al., using different materials, discussed the application of NIR lasers in the context of irradiation in antibacterial research.^[^ ^51^ ^]^ Zinc‐doped ceria acts as a catalyst that promotes the production of ROS. The increased production of ROS is a prominent part of the demonstrated bactericidal activity. The PPY coating could improve the delivery and retention of MB at the infection site and thereby allow for a more concentrated and efficient ROS generation.^[^ ^52^ ^]^ The synergistic PTT‐PDT effect of CeZn@PPY@MB NFs enhances the permeability of bacterial membranes so that bacteria become more susceptible to MB and ROS generated by MB, facilitating more penetration into the bacteria, thus achieving good antibacterial results, causing an increase in the inhibition zone. Yang et al. showed that increased levels of ROS can disrupt bacterial membranes, which is likely linked to a corresponding increase in the zone of inhibition.^[^ ^53^ ^]^ The following phenomenon was further quantified by colony counting assay using both SA and MRSA. The spread plate method was employed to culture 12 h nanoflower‐bacteria dispersions that were either treated with or without NIR 808 irradiation. The number of colonies, respectively, for each kind of bacteria, for different treatment groups, was quantified, and the percentage survival rate of the colony‐forming units was calculated. It was verified that the CeZn@PPY@MB NFs showed a significant decrease (*p* < 0.001) in the percentage survival rate value of 2.43 ± 0.17% for MRSA (**Figure** **5**A,B) and 0.19 ± 0.42% for SA (Figure S4A,B, Supporting Information) on receiving NIR 808 irradiation, compared to the control with laser add‐on treatment.

The influence of PTT‐PDT using CeZn@PPY@MB NFs on staphyloxanthin levels in MRSA and SA was assessed. Staphyloxanthin, a carotenoid pigment produced by the bacterium *Staphylococcus aureus* (SA) or its methicillin‐resistant variant (MRSA), protects the bacterium against ROS generated during the immune response of the host, thus increasing its virulence. The inhibition of its biosynthesis by SA would render it more sensitive to oxidative stress. Sun et al. inform about naftifine, an existing inhibitor of staphyloxanthin biosynthesis, that aids in promoting photoinactivation of SA using PDT.^[^ ^54^ ^]^ Staphyloxanthin levels were determined by a spectrophotometric assay after the application of nanoflowers and NIR irradiation at a wavelength of 808 nm on bacterial cultures. A spectrophotometric analysis of the ethyl acetate extract was conducted to determine the concentrations of staphyloxanthin (462 nm). Staphyloxanthin levels of MRSA cultures (Figure 5C) treated with CeZn@PPY@MB NFs under NIR (808 nm) irradiation substantially decreased in comparison to the control group (*p* < 0.001). This drop suggests a blockade in staphyloxanthin biosynthesis. Treatment of MRSA cultures solely with the CeZn NFs, without NIR irradiation, decreased staphyloxanthin levels, though not to a significantly lower extent than the combined treatment (*p* < 0.05) compared to the control group. Therefore, the bimetallic hybrid nanoflowers demonstrate an innate ability to inhibit staphyloxanthin production, presumably through Zn^+2^ ion release and its interaction with the bacterial cell wall. These data indicate that the CeZn@PPY@MB NFs‐mediated PTT‐PDT effectively inhibits staphyloxanthin production in MRSA strains. The inhibition arose from the synergetic effects of the components of CeZn@PPY@MB NFs.^[^ ^55^ ^]^ PPY has a photothermal effect that can be activated by NIR irradiation, and MB, in combination with PPY, shows better PDT results compared to PDT alone, with the help of the production of ROS. ROS can inhibit enzymes involved in the biosynthetic route or generate oxidative stress that interferes with bacterial metabolism. Cecatto et al. clarify the role of MB in exerting PDT, inducing ROS, and causing a crisis in bacterial cell viability.^[^ ^56^ ^]^ Enzymes in the biosynthetic pathway may be destabilized by localized heating. Zn^+2^ ions in the nanoflowers may play roles in staphyloxanthin biosynthesis inhibition. Zn^+2^ ions have antibacterial effects and are likely to inhibit bacterial metabolism, potentially affecting the staphyloxanthin pathway. Liu et al. evaluate ZnO nanoparticles as a wound dressing, with a focus on the antibacterial characteristics of zinc.^[^ ^57^ ^]^ The extent of staphyloxanthin biosynthesis inhibition is particularly meaningful based on diabetic wound healing. Nanoflower‐mediated depletion of staphyloxanthin renders MRSA more susceptible to oxidative stress and immune attacks from the host. Improved microbial clearance promotes faster wound healing.

The intracellular level of ROS is notably increased in bacteria treated by CeZn@PPY@MB NFs combined with irradiation. This indicates that CeZn@PPY@MB NFs is used as a PTT‐PDT‐catalytic mediated ROS generator by a photothermally enhanced POD activity. Figure 5D,E show that CeZn@PPY@MB NFs plus NIR irradiation significantly increased the level of bacterial cell 2′7′‐dichlorofluorescein (DCF) fluorescent intensity compared to the control group (*p* < 0.001). To solidify the notion, the intracellular GSH levels with various treatments were explored further; as an endogenous antioxidant defense system, the impact of glutathione can protect bacteria from oxidative stress, minimizing the therapeutic effect of any attempted treatment.^[^ ^58^ ^]^ Figure 5F shows that CeZn@PPY@MB NFs with laser irradiation significantly reduced the intracellular GSH level of MRSA in comparison to the control groups (*p* < 0.001). The findings of this study indicated that nanoflower treatment can increase GSH depletion via a photothermal effect. This is consistent with reports from other studies, according to which photothermal effects improve catalytic activity and ROS generation to inactivate bacteria.^[^ ^31^ ^]^ This intracellular GSH depletion strongly suggests a mechanism of ROS‐induced bacterial injury, which has also been observed in other studies of antimicrobial nanomaterials^[^ ^53^ ^]^ (Figure 5L). Bacterial viability in planktonic bacteria and biofilms was evaluated using a live/dead assay. The NIR 808 nm (1 W cm^−2^) irradiated CeZn@PPY@MB NFs were further verified for their proportionate antibacterial activity by measuring the diameters of the inhibition zone around the formulation‐infused sterile paper discs (6 mm diameter) at the MIC concentration. This was achieved through the synergistic PTT and PDT amplified catalytic effect.^[^ ^59^ ^]^ It was observed that compared to the free MB groups, with or without NIR 808 nm irradiation, the irradiated CeZn@PPY@MB NFs group showed a significantly larger diameter of the inhibition zone (Figure 5G,H) (Figure S4C,D, Supporting Information). Figure 5I shows a tendency for MRSA and SA (Figure S4E, Supporting Information) to experience destabilization of the membrane potentials after treatment with NIR irradiated nanoflower dispersions, where the CeZn@PPY@MB NFs caused the highest level of destabilization in both test organism populations. Additionally, the content of the intracellular protein leakage from the treated bacteria population was evaluated and quantified by Bradford reagent which presented the NIR irradiated CeZn@PPY@MB NFs treatment to produce the highest intensity of intracellular protein leakage from MRSA (Figure 5K), compared to that in the absence of NIR irradiation (Figure 5J), as well as SA (Figure S4F,G, Supporting Information) populations, in comparison to the control (*p* < 0.001).

**Figure 6:**
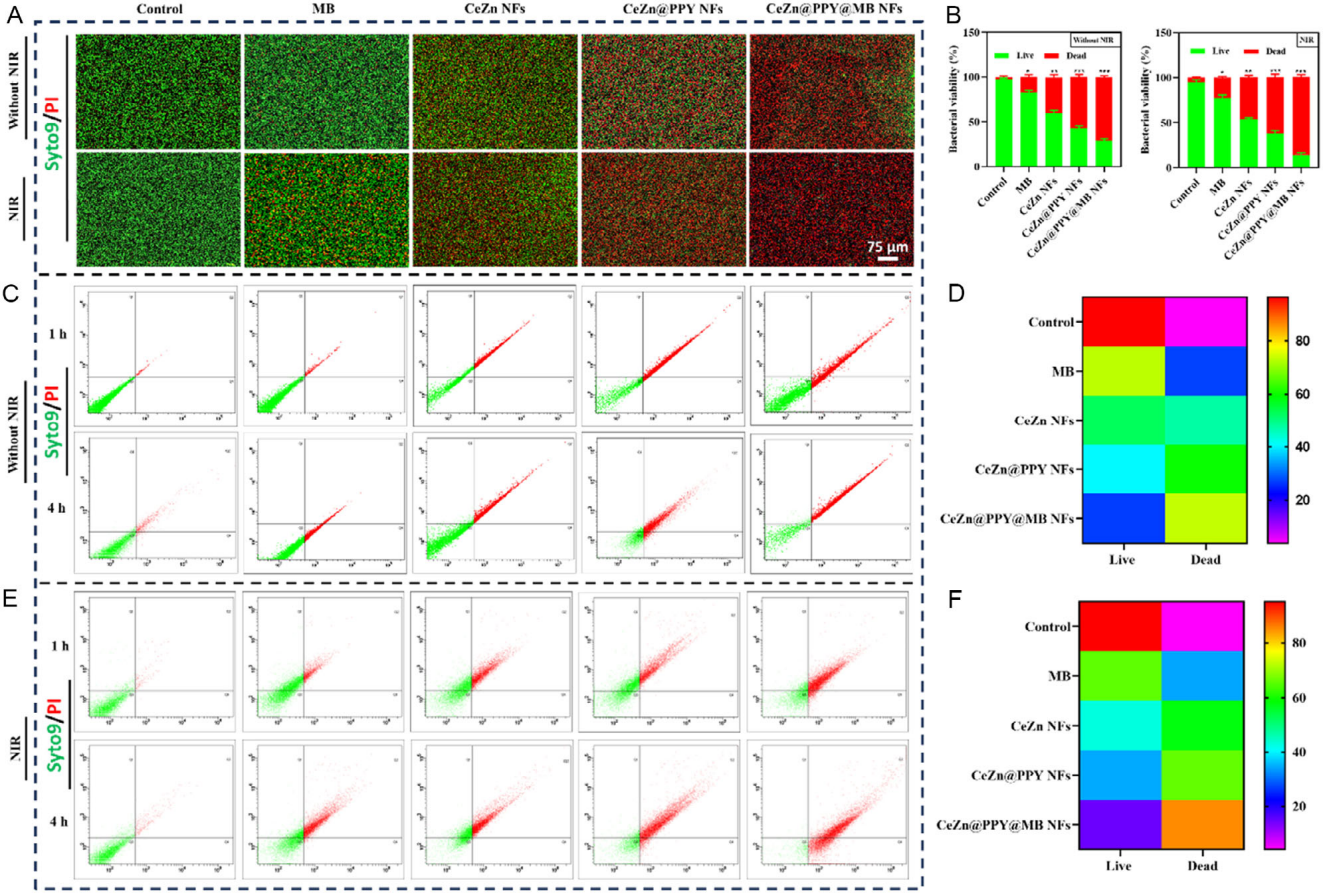
In vitro Syto9 and PI stained live/dead assay (green‐live, red‐dead) of methicillin‐resistant *Staphylococcus aureus* (MRSA) incurring treatments due to free MB, CeZn NFs, CeZn@PPY NFs, and CeZn@PPY@MB NFs without and with NIR (808 nm, 1 W cm^−2^, 5 min) irradiation, compared to PBS (pH 7.4) treated control. A) Merged images showing relative live/dead bacterial populations. Scale = 75 μm. B) Quantitative analysis of relative live/dead bacterial cell populations. C,E) The graphical representation of live and dead cell populations using flow cytometric (FACS) dot plots, without and with NIR irradiation, respectively. D,F) Heat maps representing relative live and dead cell populations in Q1, Q3, Q2, and Q4 quadrants of FACS‐generated dot plots, without and with NIR treatments, respectively, (*n* = 3). All data represented as mean ± SD (*p < 0.05, **p < 0.01, ***p < 0.001).

**Figure 7:**
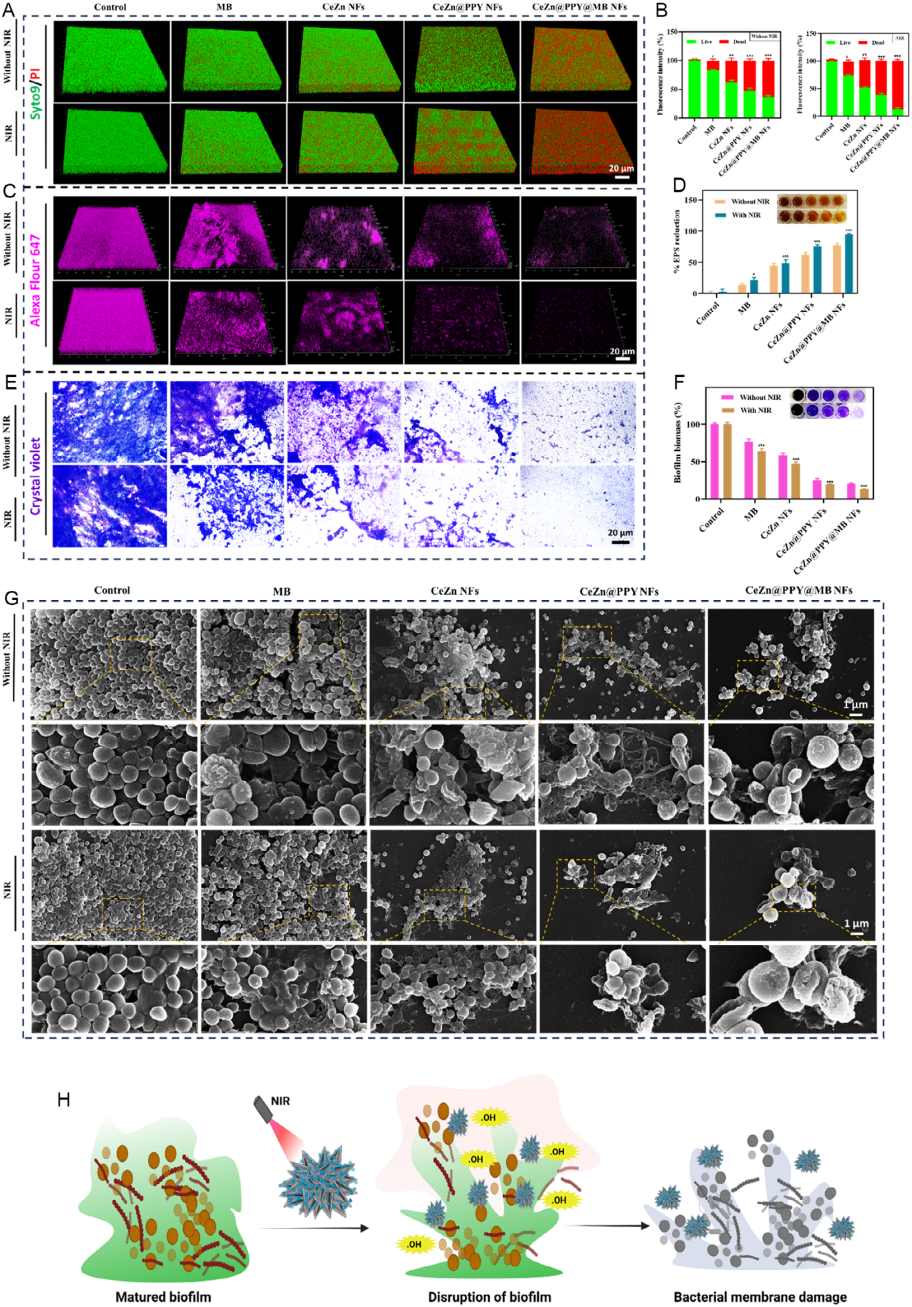
Antibiofilm activity effects of free MB, CeZn NFs, CeZn@PPY NFs, and CeZn@PPY@MB NFs treatments against methicillin‐resistant *Staphylococcus aureus* (MRSA) without and with NIR (808 nm, 1 W cm^−2^, 5 min) irradiation, compared to PBS (pH 7.4) treated control. A) 3D images of Syto 9/PI PI‐treated biofilms, visualized through a confocal microscope. Scale = 20 μm. B) Quantitative analysis of relative live/dead bacterial cell populations. C) 3D images of Alexa 647‐treated EPS expressing biofilms, visualized through a confocal microscope. Scale = 20 μm. D) Percentage of EPS expressing biofilm analysis after various treatments by the phenol sulfuric acid method. E) Bright‐field images of crystal violet‐stained bacterial biofilms. F) Percentage of biofilm biomass analysis due to various treatments. G) Biofilm disruption images due to various nanoflower treatments, free MB, and PBS‐treated control, by SEM (*n *= 3). H) A schematic representation of photothermal boosted antibiofilm activity of CeZn@PPY@MB NFs against MRSA biofilm. All data represented as mean ± SD (*p < 0.05, **p < 0.01, ***p < 0.001).

The bacteria exposed to CeZn@PPY@MB NFs exhibited the highest number of dead cells (red fluorescence by PI staining), after 4 h of treatment, in both MRSA (**Figure** **6**A,B) and SA (Figure S5A,B, Supporting Information) strains, compared to the live cells (green fluorescence by Syto 9 staining), both in the case of planktonic and biofilm populations of MRSA (**Figure** **7**A,B) and SA (Figure S6A,B, Supporting Information) with NIR irradiation, compared to the control group. The live/dead assay findings were quantitatively validated through flow cytometry. After 4 h, the analysis revealed a dead population in the Q2 and Q4 quadrants, with a total mortality rate of 85.20 ± 3.34% for MRSA (Figure 6E,F) and 87.8 ± 2.43% for SA (Figure S5E,F, Supporting Information) with NIR exposure, which is considerably higher than in the absence of NIR irradiation (Figure 6C,D). The results revealed that the CeZn@PPY@MB NFs with NIR laser irradiation had superior bactericidal efficacy compared to the other formulations, with or without NIR laser irradiation against the test organisms.

MRSA produces large amounts of EPS matrix in diabetic wounds, playing a vital role in its drug resistance and persistence. Diabetes causes high glucose levels, which leads to impaired neutrophil and macrophage function. Hirsch et al. explain a diabetic wound model's vulnerability to infections.^[^ ^60^ ^]^ This blunted immune response sets the stage for MRSA colonization and biofilm formation. After the *Staphylococci* have arrived at the site of the wound, their adhesion is often facilitated to the surface through a process known as “fibrinogen and fibronectin‐induced adhesion” by the wound bed. The bacteria then clump together and form microcolonies. As the microcolonies expand, MRSA starts making EPS, a large combination of polysaccharides, proteins, extracellular DNA (eDNA), and other macromolecules. EPS is crucial in developing the structure of biofilms; it protects the bacteria from host immune rejection and antimicrobial agents.^[^ ^61^ ^]^ The biofilm grows through a maturation phase, where a 3D, heterogeneous community of microorganisms develops with channels for the exchange of nutrients and waste. Moreover, the diabetic wound microenvironment with elevated glucose, reduced pH, and altered oxygen availability promotes both EPS expression and biofilm maturation. However, the increased availability of glucose can result in EPS production and a thicker biofilm, making it more resistant to antimicrobial agents. The polysaccharide composition of MRSA EPS is a major component and may include poly‐N‐acetylglucosamine.^[^ ^62^ ^]^ These elements play a role in their structural stability and resistance against antimicrobials. The EPS production inhibition and thus, reduction of the biofilm production phenomenon by MRSA and SA due to the treatments by various nanoflower formulations‐CeZn NFs, CeZn@PPY NFs, and CeZn@PPY NFs compared to control and free MB, with and without NIR laser irradiation, was evaluated using bright, far‐red fluorescent Alexa fluor 647 staining of the formed biofilm biomasses. 3D photographs of the EPS stained biofilm masses produced by both bacterial strains, MRSA (Figure 7C) and SA (Figure S6C, Supporting Information), after the treatment with different formulations, with or without NIR irradiation, were captured using confocal microscopy. The % EPS inhibition after the CeZn@PPY@MB NFs treatment, accompanied by NIR irradiation for 5 min, significantly reduced to a value of 94.23 ± 1.87%, compared to the control, with or without laser, when validated through the decrease in absorbance in the phenol sulfuric acid method of EPS quantification (Figure 7D). The % EPS inhibition in SA biofilm due to similar formulations has been shown in Figure S6D, Supporting Information. The combined PTT‐PDT effect due to CeZn@PPY@MB NFs on MRSA or SA inhibits EPS production through various mechanisms to promote diabetic wound healing, in which the PPY component creates localized hyperthermia to disrupt the structural integrity of the biofilm matrix, including the EPS. The heat denatures proteins and other macromolecules in the EPS, destabilizing the biofilm and allowing it to become more susceptible to the treatment. An overall boosted‐up ROS production because of PTT‐associated PDT via MB damages the bacterial DNA, proteins, and lipids, combined with the prevention of metabolic processes necessary for EPS biosynthesis.^[^ ^63^ ^]^ The PTT‐PDT method employed together can even interrupt the quorum‐sensing system in bacteria, which keeps track of bacterial proliferation and governs the behavior of the free will of bacterial beings, including their EPS production.^[^ ^64^ ^]^ In addition to this, the incorporation of zinc in the nanoflowers contributed to an overall increase in antibacterial activity. Zinc disrupts the membranes of bacteria and interrupts critical metabolic processes.

Crystal violet staining is a widely used method to quantify biofilms, including those formed by MRSA. Crystal violet is a cationic dye that binds negatively charged components of the biofilm matrix. These include negatively charged polysaccharides, proteins, and eDNA, as mentioned above. The negative charge of bacterial cell walls.^[^ ^65^ ^]^ We gave an overview of the underlying mechanism, which is based on electrostatic interactions. The biofilm matrix was stained with crystal violet, allowing for visibility. The severity of the staining is proportional to the quantity of biofilm.^[^ ^66^ ^]^ Photographs of the stained biofilm masses of the test organisms (SA and MRSA) at the end of 20 min of staining with 0.1% crystal violet (50 μL) were obtained and presented in Figure 7E, which illustrates the groups of the treatments made up of CeZn, CeZn@PPY, and CeZn@PPY@MB NFs, with or without NIR 808 irradiation. 95% ethyl alcohol was used to solubilize the bound crystal violet staining from the stained biomass cells. The solubilized dye was measured for absorbance using a UV–vis spectrophotometer with a wavelength of 570 nm. The absorbances registered were directly proportional to the biofilm mass amount obtained after treatment with the different nanoflower dispersions. Among all kinds of treatments, CeZn@PPY@MB NFs combined with laser treatment presented a remarkable reduction of 86.44 ± 0.16% in the % biofilm mass value of MRSA (Figure 7F) compared to the PBS‐treated control. Data on the reduction in the biofilm mass of SA by the above treatments is represented in Figure S6E,F, Supporting Information. MRSA produces mainly anionic biofilms consisting of polysaccharides such as poly‐N‐acetylglucosamines as well as proteins. The bimetallic hybrid nanoflowers, exerting a synergistic PTT‐PDT effect, coupled with enhanced ROS production as a result of mild hyperthermia, may have promoted the disruption process of the bacterial biofilm by compromising the bacterial cell membrane zeta potential and led to reduced biofilm mass production. The distortion of bacterial cells and disruption of bacterial biofilm by the action of CeZn NFs, CeZn@PPY NFs, and CeZn@PPY@MB NFs were confirmed by the visualization under SEM. The CeZn@PPY@MB NFs showed greater disintegration of both MRSA and SA biofilms when exposed to NIR, contributing to the photothermal (PTT) phenomenon‐related release of metal ions (Figure 7G,H) and Figure S6G, Supporting Information.

### 2.5 In Vitro Biological Activity and Blood Compatibility of Ceria‐Zinc Nanoflowers

In normal individuals, the wound healing process comprises a complex, time‐dependent series of events that can be broadly classified into four distinct phases: coagulation and hemostasis, inflammation, proliferation, and remodeling. Multiple interactions, including cells, growth factors, and extracellular signals, are required to start and run the repair process. In diabetic patients, an unfavorable microenvironment comprising high levels of ROS and hypoxia within the wound microenvironment reduces the survival and proliferation of skin cells, including keratinocytes, fibroblasts, and vascular endothelial cells. To combat this situation, CeZn@PPY@MB NFs comprise ceria and zinc metals infused in the form of a high surface area providing nanoflower structure, coated with PTT providing PPY, and loaded with hypoxia‐modulating PDT providing photosensitizer MB. The cell proliferation boosting activity of the CeZn, CeZn@PPY, and CeZn@PPY@MB NFs compared to untreated and free MB treated NIH‐3T3 murine fibroblast cells, with or without NIR laser irradiation (808 nm, 1 W cm^−2^). Briefly, NIH‐3T3 fibroblast cells were cultured in Dulbecco's Modified Eagle Medium (DMEM) in addition to 10% FBS and 1% penicillin‐streptomycin. The cells, at 7 × 10^3^ cells/well density, were seeded in 96‐well plates and allowed to adhere. Following that, in combination with media, MIC concentrations of CeZn, CeZn@PPY, CeZn@PPY@MB, free MB, and PBS 7.4 (control) plus NIR 808 laser treatment (1 W cm^−2^) for 5 min were given.

**Figure 8:**
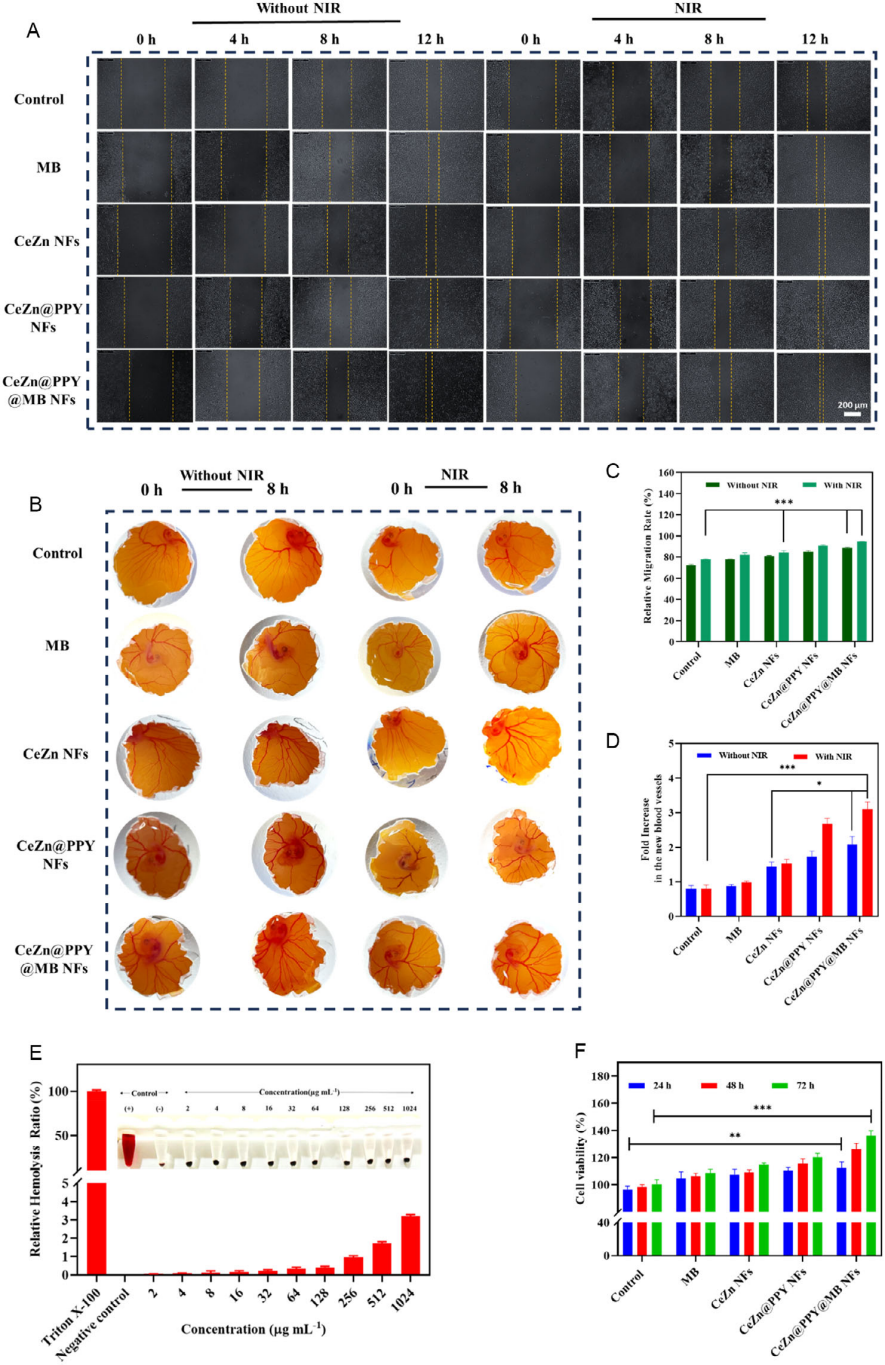
In vitro biological activity and blood compatibility of ceria‐zinc nanoflowers. A) Cell migration capability analysis of CeZn NFs, CeZn@PPY NFs, and CeZn@PPY@MB NFs compared to the PBS control and free MB, with or without NIR irradiation (*n* = 3) across 0–12 h time. Scale = 200 μm. B) Images showing the effect of free MB, CeZn NFs, CeZn@PPY NFs, and CeZn@PPY@MB NFs on angiogenesis in CAM of fertilized hen eggs compared to the PBS control, without or with NIR (808 nm, 1 W cm^−2^, 5 min). C) Quantification of migration rate of NIH‐3T3 cells across the scratch after treatment with free MB, CeZn NFs, CeZn@PPY NFs, and CeZn@PPY@MB NFs versus PBS 7.4 control, without or with NIR irradiation at the end of 12 h (*n* = 3). D) Quantification of fold increase in the new blood vessels after treatment with free MB, CeZn NFs, CeZn@PPY NFs, and CeZn@PPY@MB NFs versus PBS 7.4 control, without or with NIR irradiation at the end of 8 h (*n* = 3). E) Relative hemolysis ratio of various concentrations of CeZn@PPY@MB NFs and corresponding photograph (*n* = 3). F) MTT assay‐based in vitro cell viability assessment of NIH‐3T3 cells under various ceria‐zinc nanoflower dispersions (*n* = 3). All data represented as mean ± SD (*p < 0.05, **p < 0.01, ***p < 0.001).

The above activity was further verified, qualitatively and quantitatively, by the in vitro scratch assay onto 90% confluent, 96‐well plate‐seeded, NIH‐3T3 cell monolayers. Following the generation of midline scratches into different monolayer‐adhered wells and treatment with laser‐irradiated bimetallic hybrid ceria‐zinc nanoflowers, substituting PBS 7.4 treatment for the control group, the plate was incubated at 37 °C. Scratches were photographed at different time points (0, 4, 8, and 12 h), and scratch areas for different treatment groups were measured using ImageJ software (**Figure** **8**A). As expected, the CeZn@PPY@MB NFs showed a significantly high cell migration rate of 96.34 ± 0.69% (*p* < 0.001) over the control group (Figure 8C). Moreover, the application of NIR 808 laser irradiation provided an extra boost to cell proliferation when compared to similar treatment groups, but without laser irradiation. This enhanced cell proliferation observed in the treatment group is likely due to the synergistic benefits of the multiple components of the nanoflowers. The antioxidant and mitogenic properties of the bimetallic hybrid ceria‐zinc nanoflowers, as well as the PTT of the PPY coating (upon NIR irradiation), resulted in improved cell viability and proliferation compared to the control, free MB, and uncoated CeZn NFs.^[^ ^42^ ^]^ Meanwhile, the loaded MB in the bimetallic hybrid nanoflowers can serve as a photosensitizer upon NIR irradiation, further promoting the wound healing response.

The angiogenic and neovascularization‐promoting capability of CeZn, CeZn@PPY, CeZn@PPY NFs, and free MB was assayed by application onto the chorioallantoic membrane (CAM) of chick embryos, with and without NIR 808 laser irradiation (1 W cm^−2^, 5 min). Both scenarios treated the control group with PBS (pH 7.4). The formation of new blood vessels was monitored for various time points (0, 2, 4, and 8 h) by digital photography and quantitatively measured by ImageJ software. It was observed that all the formulations promoted the formation of new blood vessel branches over successive time points, but the 5 min NIR irradiated CeZn@PPY@MB NFs significantly amplified the neovascularization phenomenon at the end of 8 h (Figure 8B). The photographs showing angiogenic effects due to the various formulations onto the CAM, with or without NIR irradiation, for 2 and 4 h, have been provided in the Supporting section (Figure S2, Supporting Information). The CeZn@PPY@MB NFs significantly increased (*p* < 0.001) the formation of new blood vessels by 3.34 ± 2.95 compared to the control group at the end of 8 h (Figure 8D). Cerium oxide nanoparticles have been validated with the ability to quench ROS.^[^ ^67^ ^]^ In such a system, the ceria portion of the nanoflowers could keep the levels of ROS at appropriate levels to promote angiogenesis and not cause detrimental effects. Moreover, the zinc component is well known to increase cell proliferation.^[^ ^68^ ^]^ Zhao et al. have researched a variant of the same nanomaterial for wound healing, a process in which angiogenesis (formation of new blood vessels) takes place.^[^ ^69^ ^]^ The PPY coating will absorb NIR light, thus generating heat at the nanoscale level. This mild photothermal effect has been shown to upregulate the expression of pro‐angiogenic growth factors such as vascular endothelial growth factors, which promote endothelial proliferation, migration, and tube formation and lead to angiogenesis. In the case of mild hyperthermia, MB, as a photosensitizer, can be triggered to produce ROS via NIR irradiation.^[^ ^70^ ^]^ Though excessive ROS can have cytotoxic effects, the regulated generation of ROS can activate signaling cascades that promote angiogenesis. Hah et al. describe how the use of MB in PDT can be tailored to an angiogenic setting. Additionally, the simultaneous action of these elements on the nanoflower structure may lead to synergistic effects, amplifying the overall angiogenic response. Moreover, the enhanced uptake of MB by the cells through the photothermal effect may cause more efficient formation of ROS for the stimulation of angiogenesis.^[^ ^71^ ^]^

The blood compatibility of CeZn@PPY@MB NFs was evaluated with the rat erythrocytes, as shown in Figure 4. 100 μL erythrocyte suspension was added to various concentrations of CeZn@PPY@MB nanoflowers (2, 4, 8, 16, 32, 64, 128, 256, 512, 1024 μg mL^−1^). As shown in Figure 8E, hemolysis of erythrocytes was negligible in CeZn@PPY@MB NFs even at concentrations up to 1024 μg mL^−1^. The absorbance of the supernatant, as measured at 576 nm, was comparable to that of the normal saline group, which demonstrated that the hemolysis rate caused by the CeZn@PPY@MB NFs was less than 5%, confirming that the nanoflowers have favorable biocompatibility.^[^ ^72^ ^]^ The methyl thiazolyl diphenyl‐tetrazolium bromide (MTT) test was used to evaluate cell proliferation at various time points (24–72 h). It was observed that various ceria‐zinc nanoflower formulations treated at their MIC concentrations, followed by laser irradiation, showed minimum toxicity to the fibroblast cells. Moreover, CeZn@PPY@MB NFs significantly boosted fibroblast cell proliferation over 72 h of treatment (*p* < 0.001) compared to control and free MB groups (Figure 8F), showing the nanoflowers to be nontoxic to normal fibroblast cells even after laser irradiation.

### 2.6 The Ceria‐Zinc Nanoflowers Improved Diabetic Wound Healing In Vivo

**Figure 9:**
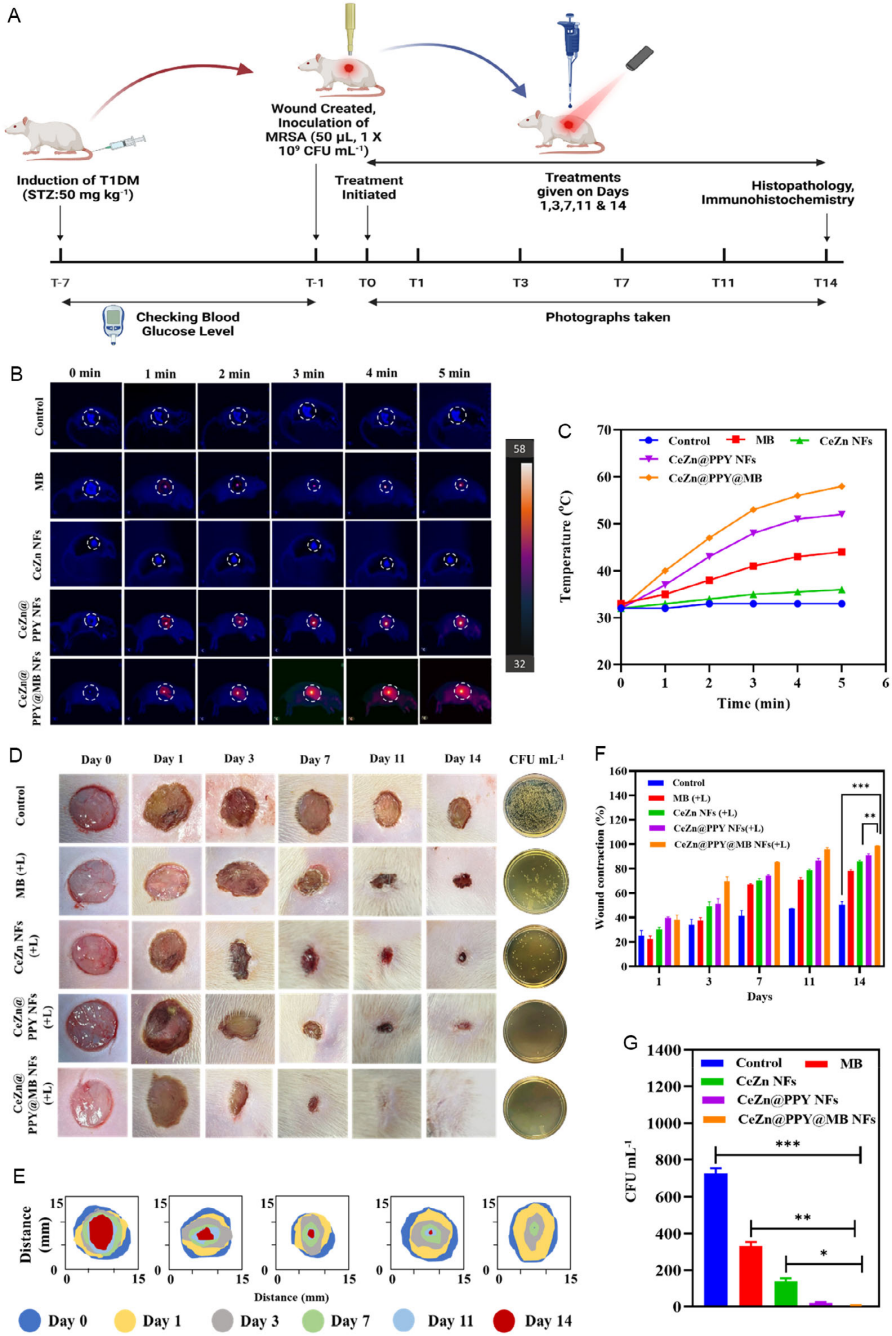
In vivo wound healing efficacy of CeZn NFs, CeZn@PPY NFs, and CeZn@PPY@MB NFs in diabetic rats compared to PBS (pH 7.4) treated control and free MB, followed by NIR (808 nm, 1 W cm^−2^, 5 min) irradiation. A) Schematic representation of the timeline for the entire therapeutic efficacy study. B) Thermal images of wounds treated with various nanoflower dispersions. C) Corresponding temperature increase curves around the wound area. D) Images of wounds on different days of treatment, followed by images of MRSA colonies formed after plating the 14th‐day wound tissue homogenate. E) Trace images of wound closure on days 0, 1, 3, 7, 11, and 14. F) Relative wound closure efficacy analysis of different treatments on various days of treatment. G) Analysis of MRSA colony counts formed after plating the 14th‐day wound tissue homogenate (*n *= 5). All data represented as mean ± SD (*p < 0.05, **p < 0.01, ***p < 0.001).

The therapeutic effectiveness of the NIR‐responsive Ceria‐Zinc nanoflowers was investigated on diabetic rats. A diabetic wound rat model with infection was constructed to investigate the therapeutic efficacy of the NIR‐responsive CeZn NFs, and the entire treatment regimen is represented in the form of a schematic diagram (**Figure** **9**A).

The in vivo photothermal potency of the CeZn@PPY@MB nanoflowers was explored after topically applying them to the wound. After its application, the wound surfaces were exposed to an NIR laser of 808 nm wavelength at an intensity of 1 W cm^−2^. Meanwhile, the temperature of the exposed region was recorded at 1 min intervals for 5 min, and infrared thermal images of the wound area were captured subsequently (Figure 9B). An approximate temperature rise of 55 °C was recorded after 5 min of irradiation to the CeZn@PPY@MB nanoflower suspension‐dosed wound region compared to the free MB application, which exhibited a small increase in temperature of 44 °C (Figure 9C). Images of wounds were taken on days 0, 1, 3, 7, 11, and 14. It was observed that the three formulations, namely CeZn NFs, CeZn@PPY NFs, and CeZn@PPY@MB NFs, showed similar wound area closure compared to MB and PBS (pH 7.4), without laser irradiation (Figure S7A,B, Supporting Information).

However, on laser irradiation, the CeZn@PPY@MB NFs showed relatively greater wound area closure compared to the other formulations, CeZn NFs and CeZn@PPY NFs (Figure 9D,E). On the 14th day of treatment, the wound area treated by CeZn@PPY NFs or CeZn@PPY@MB NFs and laser irradiation showed noticeable healing compared to the diabetic control and MB treatment. On the 14th day of therapy, the CeZn@PPY@MB NFs‐treated group had a substantial rise in the percentage wound closure value of 98.86 ± 0.089% compared to only 50.40 ± 2.58% for the diabetic control group with NIR laser irradiation, as revealed in Figure 9F (*p* < 0.001). The trace images of wounds on various days are represented in Figure 9E.

On the 14th day of the study, treatment and control group animals were anesthetized using 5% isoflurane and oxygen inhalation. Eppendorf tubes with PBS were used to retain the wound tissues after they were dissected and aseptically removed (pH 7.4). Subsequently, the wound tissues underwent homogenization for 2 min at 3500 rpm by the usage of a high‐speed homogenizer. The diluted samples were plated on nutrient agar plates and incubated at 37 °C for 12–18 h. To ascertain the quantity of viable bacteria, the colonies that developed on plates were counted. The bacterial load for diabetic control, MB, CeZn, CeZn@PPY, and CeZn@PPY@MB NFs without laser irradiation was found to be 604.33 ± 39.71, 397 ± 20.66, 193.66 ± 16.56, 28.33 ± 6.50, and 18 ± 3.60 CFU mL^−1^, respectively (Figure S7C, Supporting Information). After laser irradiation, the wound tissues with CeZn@PPY@MB nanoflower treatment showed the minimum bacterial load on the wound compared to diabetic control wound tissue, which received PBS (pH 7.4) treatment only, as shown in Figure 9G, and a CFU of 5.33 ± 2.08 CFU mL^−1^ respectively (*p* < 0.001), whereas the free MB showed a CFU value of 331.66 ± 21.12 CFU mL^−1^. The CeZn NFs and CeZn@PPY NFs‐treated tissue homogenates showed CFU values of 138.33 ± 16.01 and 19.66 ± 5.68 CFU mL^−1^, respectively. The therapeutic efficacy of CeZn@PPY@MB NFs was also evaluated in a nondiabetic wound model, where it showed faster wound healing with ≈99% wound closure at the end of only 11 days, with visible healing of the wound scar by the end of the 14th day (Figure S10, Supporting Information).

The mechanism underlying the enhanced antimicrobial activity can be articulated as follows: the bimetallic hybrid ceria‐zinc nanoflowers function as a substrate for the incorporation of the photosensitizer MB. Under 808 nm NIR laser irradiation, MB molecules produce ROS, including singlet oxygen (^1^O_2_) and superoxide radicals ($O_{2^{-}}$), which can effectively eliminate bacterial cells.^[^ ^73^ ^]^ The application of PPY coating on the nanoflowers improves the stability and photosensitizing characteristics of the system, thereby enhancing its antimicrobial efficacy.^[^ ^74^ ^]^ The wound tissues of ≈100 mg were excised from animals (*n* = 3) after 5 min of application of CeZn@PPY@MB NFs combined with NIR irradiation. The tissue Ce and Zn metal ions were analyzed using inductively coupled plasma‐mass spectrometry (ICP‐MS). The analysis revealed that after 5 min of treatment, 3.73 ± 0.21 ppm Ce and 3.08 ± 0.13 ppm of Zn were present, revealing optimal wound tissue saturation (Table S1, Supporting Information).

**Figure 10:**
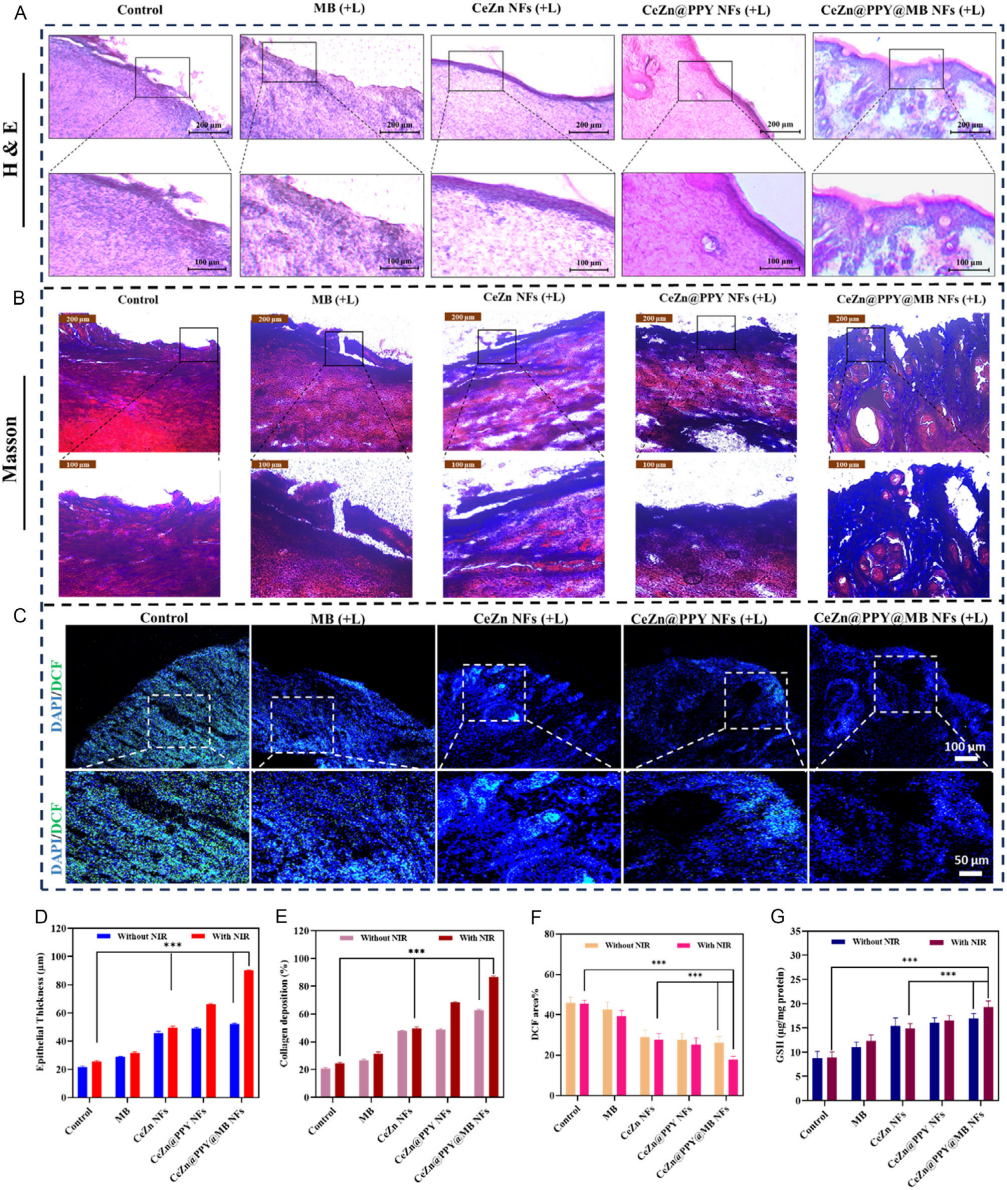
The in vivo efficiency of wound healing of CeZn NFs, CeZn@PPY NFs, CeZn@PPY@MB NFs, and free MB, followed by NIR (808 nm, 1 W cm^−2^, 5 min) irradiation, was evaluated in diabetic rats compared with PBS (pH 7.4) through histological evaluation of the wound tissues at day 14. A) H&E staining and B) Masson's staining of wound tissue on day 14. C) ROS content of wound tissue at day 14. D–F) Quantitative analysis of epithelial thickness, collagen deposition, and ROS, respectively. G) Wound tissue GSH content on day 14. Scale:100 μm; Data are represented as mean ± SD. Sample size, *n* = 3. All data represented as mean ± SD (*p < 0.05, **p < 0.01, ***p < 0.001).

Excision of the wound tissues was conducted on the 14th day of treatment, and histological investigations were performed to assess the quality of the reconstituted skin. Analysis mediated through H & E staining demonstrated that the CeZn@PPY@MB treatment group, being irradiated by laser, demonstrated enhanced neovascularization, expedited re‐epithelialization, and diminished inflammatory cell infiltration on day 14 in comparison to the other groups (**Figure** **10**A). The following can be attributed to the increased photothermal (PTT) and photocatalytic effects leading to their antibacterial and angiogenic capabilities, the suppression of inflammation, and in situ oxygen generation promoted by CeZn@PPY@MB NFs. On the 14th day, the control groups showed possession of large areas with underdeveloped granulation tissue encircling the skin incision. Compared to the laser‐untreated groups, the laser‐treated CeZn@PPY@MB NFs treatment group showed an exceptionally regular epithelium with nearly complete dermal regeneration (Figure 10D). Furthermore, Masson's trichrome staining demonstrated markedly elevated collagen deposition of 86.97% in wound tissues treated with CeZn@PPY@MB NFs under laser irradiation, as revealed through the intensity of blue color, relative to diabetic control groups (Figure 10B,E). The elevated collagen levels and tissue regeneration result from the augmented photothermal effect, which promotes the upregulation of essential growth factors and signaling pathways associated with wound healing.^[^ ^75^ ^]^ The CeZn@PPY@MB NFs treatment followed by laser irradiation significantly increased the tissue glutathione level (Figure 10G), which can be attributed to its excellent anti‐inflammatory activity, following efficient mitigation of oxidative stress and restoration of antioxidant homeostasis. The histological findings against treatment with free MB, CeZn NFs, CeZn@PPY NFs, and CeZn@PPY@MB NFs without the application of NIR irradiation are shown in Figure S8, Supporting Information.

To assess the ROS content and hypoxia levels in diabetic wounds following ceria‐zinc nanoflower treatment, immunofluorescence staining was performed using the ROS probe 2′7′‐dichlorodihydrofluorescein diacetate (DCFH‐DA) that oxidized to form 2′7′‐dichlorodifluorescein (DCF). Following treatment with CeZn@PPY@MB NFs and later laser irradiation, a notable decrease in DCF fluorescence was seen in the wound tissues collected on the 14th day postsurgery (Figure 10C,F), therefore proving its efficacy in scavenging ROS. The marked decrease in DCF levels displayed by the laser‐irradiated CeZn@PPY@MB NFs group indicated the cooperative synthesis of oxygen by CeZn@PPY@MB NFs. ROS produced by the continuous oxygenation in diabetic wounds may be efficiently captured by NIR irradiated CeZn@PPY@MB NFs, so for minutes, a beneficial cycle that supports a suitable microenvironment for diabetic wound healing.^[^ ^76^ ^]^

**Figure 11:**
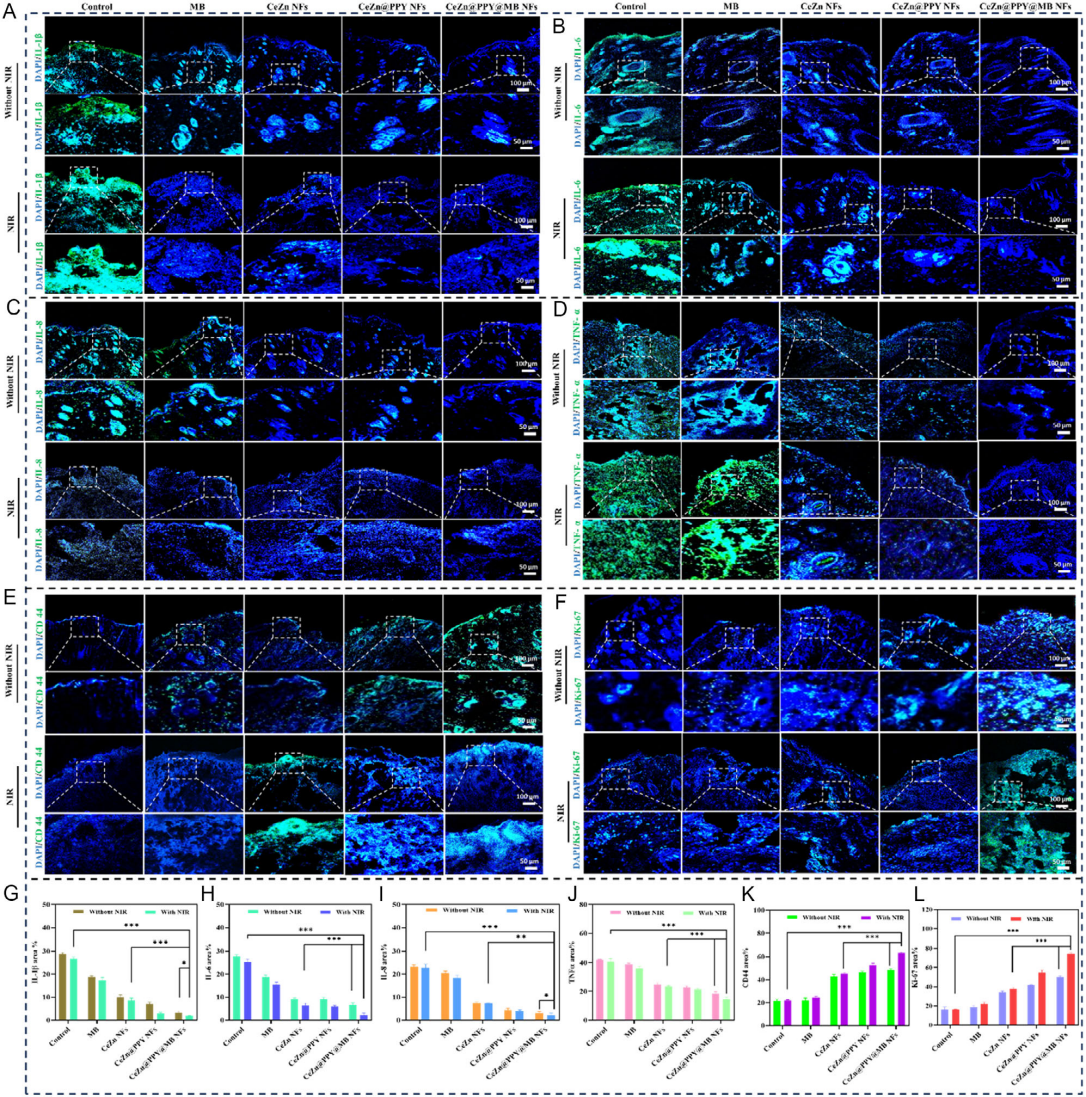
Immunofluorescence staining of various markers involved in diabetic wound tissue healing after treatment with CeZn NFs, CeZn@PPY NFs, CeZn@PPY@MB NFs, and free MB, without and with NIR irradiation (808 nm, 1 W cm^−2^, 5 min), compared to PBS (pH 7.4) treated control, on day 14. A) IL‐1β expression. B) IL‐6 expression. C) IL‐8 expression. D) TNF‐α expression. E) CD‐44 expression. F) Ki‐67 expression. G) Relative quantitative IL‐1β expression. H) Relative quantitative IL‐6 expression. I) Relative quantitative IL‐8 expression. J) Relative quantitative TNF‐α expression. K) Relative quantitative CD‐44 expression. L) Relative quantitative Ki‐67 expression. Scale bars are 100 μm. Data are represented as the mean ± SD, (*n *= 3) (*p < 0.05, **p < 0.01, ***p < 0.001).

Diabetes is a chronic condition that may result in numerous complications, including compromised wound healing.^[^ ^77^ ^]^ A major impediment to diabetic wound healing is heightened inflammation, which can further prolong the healing process. The levels of inflammatory markers were evaluated at the tissue level to analyze the anti‐inflammatory potential of CeZn, CeZn@PPY, and CeZn@PPY@MB NFs compared to PBS‐treated diabetic control or free MB, with and without NIR. It was observed (**Figure** **11**A–D) that the CeZn NFs themselves showed minimal anti‐inflammatory effects by reducing IL‐1β, IL‐6, IL‐8, and TNF‐α levels in comparison to diabetic control (*p* < 0.01). These can be attributed to its inherent catalytic properties (e.g., SOD, CAT, POD), neutralizing ROS that drive inflammation. Previous studies have revealed that the reduction of in situ ROS levels can indirectly lower inflammatory cytokine levels.^[^ ^78^ ^]^ Interestingly, CeZn@PPY, or, CeZn@PPY@MB NFs, showed a synergistic increase in anti‐inflammatory effects plus NIR laser irradiation through a significant decrease (*p* < 0.001) in levels of pro‐inflammatory components like IL‐1β, IL‐6, IL‐8, and TNF‐α (Figure 11G–J), compared to diabetic control, free MB, or uncoated CeZn NFs (with, or without NIR).

Moreover, CeZn@PPY NFs, or CeZn@PPY@MB NFs, along with laser irradiation, considerably increased the level of CD44 (Figure 11E), a cell surface glycoprotein, and Ki 67 expression (Figure 11F), a key driver for cell proliferation, compared to diabetic control, free MB, or uncoated CeZn NFs, and quantified (Figure 11K,L). CeZn@PPY@MB NFs possess the following properties that can explain the above phenomenon. Ceria nanoparticles have inherent natural antioxidant enzyme‐mimicking properties that can scavenge ROS. Along with zinc, which plays a crucial role in cell proliferation activity, generated in the form of nanoflower, which provides a high surface area for enhanced catalytic activity, coated with the PTT‐inducing photothermal agent PPY, and loaded with the PDT‐producing photosensitizer MB, it generates ^1^O_2_ and other ROS, that can kill bacteria at the wound site and disrupt the biofilm formation when irradiated by NIR, causing mild hyperthermia. Moreover, the bimetallic hybrid nanoflowers have shown ROS scavenging action, mitigating oxidative stress and inflammation. Decreased ROS levels can cause the induction of several growth factors related to tissue regeneration, thus enhancing cell proliferation, angiogenesis, and extracellular matrix (ECM) deposition, which ultimately accelerates the wound healing procedure. CeZn@PPY@MB NFs have been shown to possess good photothermal conversion efficiency and produce mild hyperthermia. Heat improves blood flow to the site of the wound, therefore supplying more oxygen and nutrients needed for tissue healing. Enhanced neovascularization can help to upregulate both CD44 and Ki‐67 expressions.^[^ ^76^ ^]^ Controlled hyperthermia can even alter the inflammatory response, which is important as diabetic wounds are often marked by continuous inflammation. This modification can provide a suitable milieu for processes driven by CD44 and tissue regeneration. Enhanced by the characteristics of CeZn@PPY@MB NFs, the combined photothermal and photodynamic activities produce a synergistic impact that might heighten the individual advantage, leading to tissue regeneration at the wound sites. The CeZn@PPY@MB NFs and the MOF‐818/Gel system reported in the work of Chao et al., both target oxidative stress regulation and inflammation control in chronic wound healing, but they differ in mechanism breadth and functional scope. MOF–818/Gel focuses primarily on potent, sustained ROS scavenging via high SOD‐ and CAT‐like activities, effectively decomposing superoxide radicals and H_2_O_2_ to relieve oxidative damage, lower ROS‐positive cell density, and shift macrophage polarization from pro‐inflammatory M1 to anti‐inflammatory M2 phenotypes, thereby suppressing IL‐6, IL‐1β, and TNF‐α levels and promoting collagen deposition, angiogenesis, and epithelial regeneration, all without an external trigger, achieving long‐term antioxidative therapy in diabetic wounds. In contrast, CeZn@PPY@MB NFs exhibit dual‐phase redox modulation: at acidic/infected stages, strong POD‐like activity (amplified by NIR‐driven photothermal and MB‐mediated photodynamic effects) generates ·OH for rapid pathogen clearance and biofilm disruption, while at neutral/healing pH, they leverage high CAT‐ and SOD‐like activities for ROS scavenging, antioxidant homeostasis restoration, GSH preservation, and anti‐inflammatory effects, evidenced by reduced cytokine expression and improved angiogenesis and tissue repair. Thus, while MOF–818/Gel offers sustained antioxidant and anti‐inflammatory benefits for chronic wounds, the CeZn@PPY@MB platform combines stimuli‐responsive antimicrobial oxidative bursts with subsequent ROS scavenging and inflammation suppression, making it more adaptable to the dynamic redox demands of infected diabetic wound healing.^[^ ^79^ ^]^

The in vivo biochemical parameter evaluation of CeZn NFs, CeZn@PPY NFs, and CeZn@PPY@MB revealed no significant deviations in key biochemical parameters compared to the control group, showing no visible histological aberrations in any of the major organ sections (Figure S11 and S12, Supporting Information), indicating that all nanoflower formulations exhibited excellent biocompatibility and did not induce detectable local toxicity at the wound site in treated rats. These findings are consistent with recent evidence from high‐impact studies, which demonstrate that the rational design of multifunctional nanozymes and zinc‐based nanomaterials can significantly enhance wound healing without adversely affecting systemic or local biochemical indices, thanks to their controlled reactivity and biocompatible surface engineering. Furthermore, comprehensive safety assessments in similar in vivo wound healing models have shown that nanomaterials with optimized release properties and low cytotoxicity profiles, promoting effective tissue regeneration while minimizing risks of organ toxicity and local adverse effects, show parity with the trends that are corroborated in the current work. Thus, the outcomes of the biochemical assessments reinforce that these engineered nanoflowers provide a safe platform for advanced wound healing applications, in line with state‐of‐the‐art nanozyme safety standards and therapeutic performance benchmarks.^[^ ^80^ ^]^

## 3 Conclusion

The synthesis and analysis of a novel ceria‐zinc nanoflower composite formation, followed by polypyrrole coating and MB loading (CeZn@PPY@MB NFs), is reported, which serves as an attractive candidate for diabetic wound therapy, including infection caused by resistant strains like MRSA. The CeZn@PPY@MB NFs synthesized here possess a unique water‐lily‐resembling morphology, promoting a high surface area for amplified PTT and loading of therapeutic agents. The polypyrrole (PPY) coating exhibits excellent photothermal (PTT) activity under NIR irradiation and incorporates MB, which acts as a photosensitizer to promote the photodynamic (PDT) effect. The minimal hemolysis seen even at high concentrations indicates that the composite is biocompatible, an essential factor for any potential in vivo applications. The PPY coating allows the NIR light to be easily absorbed, producing localized heat that can destroy bacteria and biofilms. Moreover, this photothermal activity is synergistically increased by the incorporated MB. In the presence of laser irradiation, CeZn@PPY@MB NFs exerted multienzyme activities, exhibited as mimicking POD, CAT, OXD, and SOD like activities, critical for modulating the wound environment and promoting healing. Improved catalytic activity not only helps with ROS elimination to protect the wound from oxidative stress but also alleviates the oxidative stress factor, which has been recognized as a significant factor negatively influencing the healing process of diabetic wounds. The composite exhibits dual photothermal and catalytic properties, demonstrating significant antibacterial cum antibiofilm efficacy in opposition to MRSA, solving a critical issue in diabetic wound treatment. In vivo experimentation utilizing a diabetic rat model demonstrated that the CeZn@PPY@MB NFs group under NIR irradiation exhibited faster wound closure, improved collagen deposition, enhanced re‐epithelialization, and reduced inflammation. The combined effects of catalytic activity, photothermal treatment, and ROS regulation are responsible for these improvements. The significant upsurge in the tissue glutathione level also strengthens the antioxidant and anti‐inflammatory activity of the NFs. In summary, the combined action of these improved properties renders CeZn@PPY@MB NFs with potential value as a new antibiotic‐depleting therapeutic platform for resistant bacterial eradication for effectively treating diabetic wounds.

## 4 Experimental Section

4.1

4.1.1

##### Materials

Zinc acetate dihydrate (Zn (CH_3_CO_2_)_2_·2H_2_O), cerium nitrate hexahydrate (Ce (NO_3_)_3_·6H_2_O), MB, crystal violet dye, hydrogen peroxide (H_2_O_2_, 30 wt%), (6aS,11bR)‐7,11b‐dihydrobenz[b]indeno[1,2‐d]pyran‐3,4,6a,9,10(6 H)‐Pentol (hematoxylin), disodium 2‐(2,4,5,7‐tetrabromo‐3‐oxido‐6‐oxoxanthen‐9‐yl)benzoate (eosin), 4′,6‐diamidino‐2‐phenylindole dihydrochloride (DAPI), pyrrole, riboflavin, methionine, titanium tetrachloride (TiCl_4_), and 5,5′‐dithiobis 2‐(nitro benzoic acid) (DTNB) (Ellman's reagent) were purchased from Sigma‐Aldrich (Bangalore, India). The MTT, nitro blue tetrazolium (NBT), 3,3′,5,5′‐tetramethyl‐benzidine (TMB), fluoromount‐G, Luria Bertini broth (LB), nutrient agar, fetal bovine serum (FBS), DMEM, trypsin, penicillin‐streptomycin, and phosphate‐buffered saline (PBS) were procured from HIMEDIA (India). The Syto 9, propidium iodide (PI), ROS assay kit DCFH‐DA, Glutathione Colorimetric Detection kit, and Alexa Fluor 647 were procured from Thermo Scientific (USA). The trichrome stain (Masson) kit was obtained from Abcam (USA). The sodium hydroxide (NaOH), sodium citrate, and Coomassie brilliant blue G‐250 dye (Bradford reagent) were procured from Sisco Research Laboratories Pvt. Ltd. (SRL, India). The embryonic mouse fibroblast cells (NIH‐3T3) were obtained from the National Centre for Cell Science (NCCS, Pune, India). Interleukin‐6 (IL‐6) antibody (D5W4V), IL‐8 antibody (E5F5Q), IL‐1β antibody (D3H1Z), TNF α antibody (D2D4), Ki‐67 antibody (D2H10), and CD44 antibody (E7K2Y) were procured from Cell Signaling Technology (CST, USA).

##### Synthesis of Uncoated Ceria‐Zinc (CeZn) Nanoflowers

CeZn nanoflowers were synthesized using the facile hydrothermal technique. The preparation of the equimolar ceria‐zinc nanoflowers involved dissolving Zn (CH_3_COO)_2_·2H_2_O (4.6 mM), cerium nitrate [(Ce (NO_3_)_3_·6 H_2_O)] (4.6 mM), and sodium citrate (2.5 mM) into a mixture of deionized (DI) water (13 mL) and absolute ethyl alcohol (13 mL). Following that, a magnetic stirrer was used to agitate the mixture continuously for a period of 1 h. Subsequent to the dissolution procedure, 4 mL of 4 M sodium hydroxide (NaOH) was introduced dropwise into the aforementioned mixture while maintaining vigorous stirring until the pH of the resultant mixture attained a value of 13. The resulting product was subsequently put into autoclaves and maintained at a temperature of 150 °C for 15 h, forming a white powder consisting of CeZn NFs. After centrifuging for 15 min at 8000 rpm, the white product was harvested. Unwanted ions were then washed away three times with ethanol and DI water, followed by vacuum drying of the product at 60 °C for 12 h.

##### Synthesis of Polypyrrole (PPY)‐Coated CeZn@PPY Nanoflowers

Black‐colored ternary CeZn@PPY nanoflowers were synthesized through in‐situ chemical oxidative polymerization of pyrrole, utilizing binary nanoflower CeZn NFs, per the methodology presented by Cheng et al.^[^ ^81^ ^]^ This approach generally involved the dispersion of 10 g of CeZn NFs in 150 mL of double‐distilled water. After the cooling process in an ice bath maintained at temperatures between 0 and 4 °C, an addition of 1.8 wt% of pyrrole was executed. Subsequently, employing a buret, 20 mL of a 2.5 m aqueous solution of FeCl_3_·6H_2_O was meticulously introduced into the mixture, all the while maintaining a consistent swirling motion. Afterward, the pyrrole polymerization reaction was permitted to proceed for an additional 8 h. Finally, the solid product was filtered and washed thrice in 100% ethyl alcohol before being dried at 60 °C overnight. Likewise, various PPY concentration‐loaded CeZn nanoflowers, such as CeZn@0.6PPYNFs, CeZn@1.2PPYNFs, and CeZn@2.4PPYNFs, were prepared.

##### Synthesis of Methylene Blue (MB)‐Loaded CeZn@PPY@MB Nanoflowers

The CeZn@PPY NFs were stirred with a 1 mg mL^−1^ concentration of MB aqueous solution for 24 h. After 24 h, the samples were washed thrice with deionized water to eliminate unbound MB. The MB adsorbed nanoflowers (CeZn@PPY@MB NFs) were then vacuum‐dried at a temperature of 60 °C overnight.

##### Characterization of NFs: Particle Size and Zeta Potential

A dynamic light scattering investigation was performed to assess the zeta potential (ζ), size distribution, and particle size at a controlled temperature of 25 °C. The Nano ZS 90 equipment, manufactured by Malvern Instruments Ltd. in the UK, was employed for this purpose. The nanoflowers, which had been dried using a vacuum, were then mixed with water for injection to get the desired concentration. The resulting dispersion was then subsequently employed for the assessment of zeta potential and particle size measurement. The inspection was conducted in triplicate, and the polydispersity index was determined using the size distribution width (PDI). PDI, or Polydispersity Index, is a quantitative assessment of the uniformity or heterogeneity of a particle solution.

##### Fourier Transform Infrared Spectroscopy (FTIR)

The analysis of functional groups and the assessment of interactions among these groups on the surfaces of CeZn NFs, CeZn@0.6PPY NFs, CeZn@1.2PPY NFs, CeZn@1.8PPY NFs, CeZn@0.6PPY@MB NFs, CeZn@1.2PPY@MB NFs, and CeZn@1.8PPY@MB NFs, in conjunction with free MB, was conducted utilizing FTIR employing the potassium bromide (KBr) pellet method. The analysis was conducted via an FTIR spectrometer (Jasco‐4200, USA). The vacuum‐dried formulations were blended with potassium bromide (FTIR grade). The spectrum was scanned over the frequency range of 4000–400 cm^−1^ at a resolution of 4 cm^−1^, with a total of 64 scans, to acquire the spectrum. The examination was performed using an FTIR spectrometer (Jasco 4200, USA). The formulations subjected to vacuum drying were meticulously combined with KBr of FTIR grade. The spectrum was critically examined across the frequency range of 4000–400 cm^−1^, employing a resolution of 4 cm^−1^ and encompassing 64 scans to obtain the spectrum.

##### Surface Morphology Analysis Using Scanning Electron Microscopy (FE‐SEM)

A structural examination of vacuum‐dried powders of CeZn@PPY@MB NFs was performed after forming an even distribution on a holder and securing it with carbon tape. After uniformly dispersing the powder, it was covered with a layer of gold and 400 mesh copper TEM grids, respectively, for 30 min. Images were obtained utilizing a field emission scanning electron microscope (FE‐SEM) and STEM (Apreo Lo Vac, FEI, USA).

##### XRD Analysis

The CeZn NFs, CeZn@PPY NFs, and CeZn@PPY@MB NFs were recorded by XRD using Cu Kα radiation. The analysis was performed using the ULTIMA‐IV Rigaku apparatus (Japan) with 40 kV voltage and 200 mA current. The fine powdered nanoflowers were characterized by analyzing the 2*θ* angles in the range of 10°–80°.

##### X‐Ray Photoelectron Spectroscopy (XPS) Analysis

The chemical properties of CeZn NFs, CeZn@PPY NFs, and CeZn@PPY@MB NFs were evaluated using XPS equipment from Thermo Fisher (USA). The XPS data acquired were analyzed using Avantage software. The C1s peak at 284.8 eV of adventitious carbon was used as a reference to correct the binding energy values of XPS lines.

##### The Photothermal Ability of Ceria‐Zinc Nanoflowers

The prepared CeZn, CeZn@PPY, and CeZn@PPY@MB NFs were evenly distributed in a 2 mL tube and exposed to a NIR laser at a wavelength of 808 nm for a duration of 5 min. The temperature was recorded every 30 s during the irradiation, and infrared thermal images were taken every minute. The average temperature increase curves of different concentrations of CeZn@PPY@MB nanoflowers were analyzed. To evaluate the photothermal stability of the as‐prepared CeZn@PPY@MB nanoflowers, a cycle of five heating/cooling processes was carried out. In short, the dispersion underwent 5 min of laser irradiation, causing it to heat up, followed by 5 min of no laser irradiation to cool. This process was conducted five times, recording the respective temperature changes. The calculation of the photothermal conversion efficiency for CeZn@PPY@MB NFs was eventually conducted using the following Equation (1)

(1) $$\eta =\frac{hs\left(T_{\text{Max}}-T_{\text{Surr}}\right)-Q_{\text{Dis}}}{I\left(1-10^{-A808}\right)}$$

*T*_Max_ denotes the peak stable temperature of the nanoflower solutions, while *T*_Surr_ indicates the ambient temperature surrounding the solution sample at the point of achieving maximum stability. It signifies the intensity of the laser light. A808 represents the absorbance at a particular wavelength of the laser light at 808 nm. *Q*_Dis_ denotes the thermal energy released from the light absorbed by the solvent and the container, while *h* signifies the heat transfer coefficient, and *S* indicates the surface area of the container.

##### Multienzyme Effects of the Nanoflowers

The nanoflowers have been shown to exhibit inherent enzyme‐like functions. The POD‐like activity of the nanoflowers was verified using 3,3″,5,5″‐tetramethylbenzidine (TMB) as the substrate. The nanoflowers were dissolved in a 500 μM solution of TMB in the presence of 1 m H_2_O_2_ and weakly acidic citrate buffer (pH 4.5). In the presence of intrinsic OXD and POD‐like activities, the initially colorless TMB gets oxidized to blue‐colored OxTMB. The reaction system comprised blank, CeZn NFs, CeZn@PPY NFs, and CeZn@PPY@MB NFs. The reaction mixture was maintained for 5 min. The reaction mixture was centrifuged, and the spectrum of the supernatant was scanned under the spectrophotometer. The CeZn@PPY@MB NFs showed the strongest intensity at 652 nm wavelength, which can be attributed to their intrinsic POD‐like enzymatic activity. Furthermore, to consolidate the intrinsic POD‐like photocatalytic capability of the nanoflowers, evaluation of Michaelis‐Menten parameters (*K*_m_ and *V*_max_) was carried out by using 100 μg mL^−1^ nanoflower dispersions and varying concentrations of TMB substrate in the presence of a fixed concentration of H_2_O_2_. In brief, nanozymes were incubated with different concentrations of TMB solutions (0.05–0.5 mM) in citrate buffer (pH 4.5) at a final volume of 200 μL, and the reactions were initiated by the addition of H_2_O_2_. The enhancement in absorbance at 652 nm was quantified using a Spectramax multiplate reader (Molecular Devices, US). The initial reaction rates were ascertained from the linear segment of the absorbance‐time profile at each TMB concentration. The Michaelis–Menten plots were constructed as initial reaction rates versus substrate concentration, whereas double‐reciprocal Lineweaver–Burk plots were employed to calculate *K*_m_ and *V*_max_ following the Michaelis–Menten Equation (2)

(2) $$v=\frac{V_{\text{max}}\;\left[\text{TMB}\right]}{K_{m}+\left[\text{TMB}\right]}$$

where *v* denotes the initial reaction rate, *V*_max_ denotes the maximum reaction rate with TMB as substrate, and *K*_m_ denotes the Michaelis constant.

The oxygen‐generating capability of different nanoflower formulations was evaluated by the addition of 1 M H_2_O_2_ into the nanoflower dispersions prepared in PBS 7.4. The evolved oxygen concentration was measured with a dissolved oxygen meter every 1 min for a span of 10 min. The same procedure was performed at different pH buffer dispersions of CeZn@PPY@MB NFs, namely 5 and 6. The H_2_O_2_ scavenging ability of the nanoflowers was further confirmed by mixing 10 mL of H_2_O_2_ (1 mM) with the nanoflowers at 37 °C in the presence of PBS 7.4. 50 μL of the supernatant was mixed with 100 μL of TiCl_4_ solution (1.33 mL of 24% TiCl_4_ mixed with 8.33 mL of H_2_SO_4_ in 50 mL of DI water) for 30 min. Absorbance was measured at 405 nm using a plate reader.

Superoxide ($O_{2^{\cdot -}}$) scavenging capacity was assessed through the calculation of the inhibition ratio associated with the photoreduction of nitroblue tetrazolium (NBT). In a standard experimental setup, riboflavin (20 μM), methionine (12.5 mM), NBT (75 μM), and samples were combined in PBS (pH 7.4) and subjected to a continuous NIR wavelength of 808 nm (1 W cm^−2^) for 10 min at 25 °C. 1 mL of the supernatant was collected, the full‐scan curve was measured, and absorbance values at 560 nm were obtained. The inhibition ratio was calculated using Equation (3)

(3) $$O_{2^{\cdot -}}\text{ scavenging ratio (}\%)=\frac{A_{\text{S }}-A_{N}}{A_{P}-A_{N}}\times 100$$

where *A*_S_, *A*_N_, and *A*_P_ represent the absorbance values of the sample, negative control, and positive control, respectively. The inhibition ratio was further checked at different pH buffer dispersions of CeZn@PPY@MB NFs, namely pH 5 and 6.

The hydroxyl radical (^•^OH) scavenging abilities of CeZn NFs, CeZn@PPY NFs, and CeZn@PPY@MB NFs were conducted utilizing salicylic acid as a probe. Solutions comprising FeSO_4_ (2 mM), H_2_O_2_ (5 mM), and salicylic acid (1.5 mM) were formulated in PBS (pH 7.4) and subjected to incubation for 30 min at a temperature of 37 °C. The Fe^2+^/H_2_O_2_ system generates ^•^OH, which can be identified through the use of salicylic acid. Salicylic acid SEQUESTers ^•^OH to synthesize 2‐hydroxy salicylic acid. An unequivocal absorption peak was detected at 510 nm. The efficiency of ^•^OH scavenging was determined utilizing the specified Equation (4)
(4)

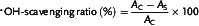

where *A*_c_ is the absorbance of the control without a sample, and *A*_s_ is the absorbance of the sample group.

##### Antibacterial Studies: Bacterial Strains

MRSA and *Staphylococcus aureus* (SA) isolates were procured from LV Prasad Eye Institute, Hyderabad, India. The culture of MRSA and SA in Luria Bertani Broth, Miller (HIMEDIA, INDIA) was conducted under aerobic conditions, and incubation was done at 37 °C temperature with 85% relative humidity. After the incubation phase, SA and MRSA (at a concentration of 10^8^ CFU mL^−1^) were introduced into a fresh culture until a bacterial concentration of 0.2 was attained at an optical density of 600 nm. All the antibacterial studies were performed in conjunction with glucose (1 mM) incubation to replicate a diabetic milieu.

##### Minimum Inhibitory Concentration (MIC)

The MIC of the formulations, namely free MB, CeZn NFs, CeZn@PPY NFs, and CeZn@PPY@MB NFs, was determined following the broth microdilution method, according to a previously established protocol. SA and MRSA were diluted in LB broth to a concentration of 10^5^ CFU mL^−1^. Thereafter, 100 μL of both bacterial suspensions and different concentrations (0.03152 to 1024 μg mL^−1^) of the formulations were incorporated into each well of 96‐well plates subjected to NIR laser irradiation for 5 min, as well as those that were not. Plates were incubated at 37 °C for 24 h. The control wells were not treated with the formulations. MIC was measured at multiple time points (0, 0.5, 1, 2, 4, 6, 8, 12, and 24 h) by assessing turbidity and optical density at 600 nm with a Spectramax microplate reader (M4, Molecular Devices, San Jose, USA).

##### Zone of Inhibition

The antibacterial efficacy of CeZn@PPY@MB NFs with and without NIR treatment against SA and MRSA was evaluated using the agar diffusion method. Bacteria were inoculated at 1 × 10^5^ CFU mL^−1^ by the method of lawn culture and distributed aseptically across the agar plate's surface utilizing sterile spreaders. Circular paper discs, measuring 6 mm in diameter, were immersed in various formulations until they reached saturation. Using sterile forceps, the discs were placed on the bacterial lawn of the agar plate and incubated for 12 h at 37 °C. Measurement of the diameter of the inhibition zone surrounding the disc was performed with the ImageJ software.

##### In Vitro *Colony Counting Assay*

To investigate the efficiency of the newly prepared nanoflowers at bacteria eradication, cultures of SA and MRSA (10^6^ CFU mL^−1^), incubated overnight, were exposed to MB, CeZn NFs, CeZn@PPY NFs, and CeZn@PPY@MB NFs with and without laser irradiation. Incubation was performed at 37 °C for 24 h. Subsequently, the plating of diluted bacterial culture onto a solid agar plate was conducted and incubated for 12 to 18 h at 37 °C. The survival rate was calculated as follows, Equation (5)

(5) $$\%\text{ Survival rate}=\frac{\text{CFU/mL of experimental group}}{\text{CFU/mL of control group}}\times 100$$

##### Intracellular ROS level evaluation

The ROS‐sensitive DCFH‐DA fluorescent probe was employed to assess the intracellular levels of ROS in bacteria after different nanoflower treatments in the presence of NIR irradiation. Bacteria in the treated groups were collected and subsequently incubated with DCFH‐DA probes (10 μM) at 37 °C for 20 min without light. The stained bacteria were then examined for their fluorescence intensity by the usage of a confocal laser scanning microscope. Ellman's reagent was used to determine the intracellular glutathione (GSH) content of the treated bacteria. After the treatments, the lysate of the treated bacteria (500 μL) was added to 5 μL of Ellman's reagent (10 mg mL^−1^), and the mixture was kept at 37 °C for 1 h, and the measurement of absorbance at 412 nm using a microplate reader allowed determining the GSH concentration.

##### Live/Dead Assay of Planktonic Bacteria: By Confocal Microscopy

SA and MRSA suspensions containing 1 × 10^6^ CFU mL^−1^ were treated with MB, CeZn NFs, CeZn@PPY NFs, and CeZn@PPY@MB NFs, with and without NIR laser irradiation. NIR laser irradiation was applied during and after treatment. The samples were incubated for 4 h at 37 °C, respectively, followed by centrifugation at 6000 revolutions/minute for 10 min at 25 °C. The supernatant was discarded, and the pellet was rinsed twice with sterile phosphate buffer saline (0.9% w/v NaCl) and resuspended in 200 μL sterile PBS. 5 μL of a 1:1 mixture of Syto 9 (33.4 μM) and PI (0.4 mM) was used to stain bacterial cells. These cells were then kept in the dark for 15 min. Confocal images were taken for stained samples (Leica, DMi8, Germany) at an excitation/emission wavelength of 485/498 nm. Syto 9 emits green fluorescence upon entering the cytoplasm of metabolically active bacterial cells, while PI emits red fluorescence only upon penetration of the bacterial cell wall in case of membrane damage.

##### Live/Dead Assay of Planktonic Bacteria: By Flow Cytometry

A flow cytometer (FACS ARIA III, BD Life Sciences, CA, USA) was used to quantify the viable and nonviable bacterial cells. An overnight culture of SA and MRSA was centrifuged at 6000 rpm for 10 min, and bacterial pellets were collected. It was washed thrice with sterile PBS (pH 7.4) and resuspended in 500 μL of PBS. After treatment with MB, CeZn NFs, CeZn@PPY NFs, and CeZn@PPY@MB NFs, with or without NIR laser irradiation, 1 × 10^6^ CFU mL^−1^ bacteria suspension was incubated at 37 °C with shaking for 1 h and 4 h, respectively. The bacterial cells were stained using the previously stated method with Syto9 and PI. Data evaluation was conducted using flow cytometry. BD FACS Diva Software was utilized to visualize data in quadrant plots. The cells located in the first (Q1) and third (Q3) quadrants denote the number of living cells, whereas those in the second (Q2) and fourth (Q4) quadrants signify the count of dying or deceased cells.

##### Protein Leakage Study by Bradford Assay

The leakage of bacterial intracellular protein by the action of the developed nanoflowers was evaluated by Bradford assay. The developed formulations, namely CeZn NFs, CeZn@PPY NFs, and CeZn@PPY@MB NFs, along with free MB and blank (PBS 7.4), were added to bacterial cultures of MRSA at their respective MIC values, following two sets of treatment regimes, without laser radiation and with NIR laser irradiation of 808 nm wavelength at an intensity of 1 W cm^−2^ for a 5 min time interval. Following the treatment, respective entities were kept inside the incubator at 37 °C for 12 h. Upon the conclusion of the incubation period, the treated bacterial cultures were centrifuged, and the supernatant was separated. 200 μL of the separated supernatant was mixed with 500 μL of Bradford reagent and incubated in the dark for 10 min. In the presence of leaked intracellular protein, a blue coloration will be formed after reacting with the Bradford reagent. The spectrum was scanned to obtain an absorption peak at 595 nm wavelength, and the peak denoting the absorption was plotted.

We then used the Bradford assay to assess the leakage of bacterial intracellular proteins induced by the action of the developed nanoflowers. The developed formulations, namely, CeZn NFs, CeZn@PPY NFs, and CeZn@PPY@MB NFs, free MB, and blank (PBS 7.4), were applied to MRSA bacterial cultures at their respective MIC concentrations. Experiments were performed using two conditions, without and under NIR laser irradiation (808 nm, 1 W cm^−2^ for 5 min). After treatment, the above‐mentioned entities were kept in an incubator at 37 °C for 12 h. After the incubation period, treated bacterial cultures were centrifuged, and the supernatant was separated. 500 μL of Bradford reagent was added to 200 μL of the separated supernatant and incubated for 10 min in the dark. If an intracellular protein is left in the solution, it will turn blue when reacting with the Bradford reagent. From spectrum scanning, an absorption peak by wavelength of 595 nm was discovered, according to which the peak was plotted.

##### Biofilm Inhibition Study: By Confocal Microscopy

A live‐dead assay of biofilm was done to check the susceptibility of bacterial biofilms to the formulation. Biofilms were developed by inoculating each well of 12‐well plates with 1 mL bacterial solution (1 × 10^5^ CFU mL^−1^) and incubating at 37 °C for 24 h. LB medium was removed after biofilm formation, and biofilms were subjected to three washes utilizing 1 mL of sterile PBS. Following that, the biofilms underwent treatments by free MB, CeZn NFs, CeZn@PPY NFs, and CeZn@PPY@MB NFs. The applications were performed with and without irradiation of the NIR laser (5 min). After the treatment, the biofilms were cultured at 37 °C for time intervals of 1 and 4 h, respectively. Bacterial cells underwent staining with Syto 9 and PI at specified final concentrations of 33.4 μM and 0.4 mM, respectively. The samples underwent staining and were incubated for 15 min in the absence of light. Bacterial biofilms treated with nanoflower dispersions were visualized in 3D using the confocal microscope (Leica, DMi8, Germany). Excitation/emission wavelengths were set to 485 nm/498 nm, respectively. Syto 9‐stained cells with intact membranes appear green when bound to DNA, allowing the identification of viable cells within the biofilm. The red fluorescence of the PI dye indicates that there are dead cells that populate the biofilm.

##### Biofilm Inhibition Study: By SEM Analysis

The biofilms were developed as mentioned above. The biofilms were incubated with nanoflowers for 4 h with and without laser exposure. Following incubation, the biofilms were gently washed with PBS and fixed in 3% glutaraldehyde at 4 °C overnight. Then, the biofilms were dehydrated with gradient ethanol concentrations (30, 50, 70, 90, and 100%), 10 min in each concentration. The samples were sputter‐coated with gold after drying and analyzed by SEM.

The biofilms were prepared as previously described. The biofilms underwent incubation with the nanoflowers for a duration of 4 h, both in the presence and absence of laser exposure. After the incubation period, the biofilms underwent a gentle washing with PBS and were fixed in a 3% glutaraldehyde solution at 4 °C overnight. Following that, the biofilms underwent dehydration through a series of ethanol concentrations (30% to 100%), devoting 10 min for each series. The samples underwent sputter‐coating with gold subsequent to the drying process and were subsequently analyzed using SEM.

##### Biofilm Inhibition Study: By the Crystal Violet Assay

Using UV spectroscopy, the antibiofilm activity of the developed nanoflowers was quantified. Cultures of SA and MRSA at a concentration of 10^5^ CFU mL^−1^, grown overnight in LB broth, were treated with the developed nanoflowers: CeZn NFs, CeZn@PPY NFs, and CeZn@PPY@MB NFs, as well as free MB and a control (PBS 7.4) at 100 μL. Treatments were performed in a sterile 96‐well microtiter plate (Therapec Solutions, India) and further incubated for 24 h at 37 °C under static conditions. After incubation, the media were removed, and the biofilm in each well underwent three washes with PBS, followed by drying of the wells for 15 min. Biofilms underwent staining with 0.1% crystal violet for 20 min. The unstained crystal violet was disposed of, and the wells were washed three times with sterile PBS and left to air dry at room temperature. The stained biofilms of each well were resuspended in 95% ethyl alcohol. Wells containing the ethanolic solution were quantitatively analyzed at OD590 with a Spectramax M4 microplate reader (Molecular Devices, San Jose, USA). The percentage of biofilm biomass was determined according to the following Equation (6)

(6) $$\%\text{ Biofilm biomass=}\frac{{\text{OD}}_{\text{Treated}}}{{\text{OD}}_{\text{Control}}}\times 100$$

##### EPS Quantification

EPS quantification was performed to evaluate the susceptibility of bacterial biofilms to formulations. The biofilms were formed by rendering each well of 12‐well plates with 1 mL of 1 × 10^5^ CFU mL^−1^ bacterial solution and placing it in an incubator at 37 °C for 24 h. LB media was starved after biofilm formation, and biofilms were washed with sterile PBS three times. Treatments corresponding to free MB, CeZn NFs, CeZn@PPY NFs, and CeZn@PPY@MB NFs, respectively, with NIR 808 irradiation (1 W cm^−2^) either present or absent, for 5 min, were applied. Confocal microscopy was utilized to evaluate the formulation's effect on EPS formation. Biofilm EPS was stained with Alexa Fluor 647 (excitation/emission of 650 nm/668 nm). Confocal microscope images were acquired using a 40 × oil immersion objective. Equation (7) was used to determine the percentage reduction of EPS as follows

(7) $$\%\text{ EPS reduction}=\frac{{\text{OD}}_{\text{Control}}-{\text{OD}}_{\text{Treated}}}{{\text{OD}}_{\text{Control}}}\times 100$$

The EPS of biofilms generated with and without different nanoflower dispersions, both in the presence and absence of NIR irradiation, was quantified using the phenol‐sulfuric acid method. Bacterial cultures were inoculated into the wells of a 12‐well plate with LB broth. The cultures received formulations and were incubated for 24 h. Following incubation, the coverslips underwent washing with 0.9% NaCl and were subsequently incubated with 500 μL of 5% phenol and 2.5 mL of concentrated sulfuric acid for 1 h at room temperature. The absorbance of the solutions was quantified at 490 nm.

##### In Vitro *Cell‐Based Studies: Cell Viability and Proliferation Assay*

The proliferation induced by CeZn NFs, CeZn@PPY NFs, and CeZn@PPY@MB NFs on NIH‐3T3 fibroblasts was evaluated by the MTT assay. The cells (4 × 10^3^) were added to 96‐well plates and incubated for 24 h in a CO_2_ incubator at 37 °C. The old media were replaced, and the cells were treated in turn with sterilized nanoflower dispersions in DMEM media for 24, 48, and 72 h. Subsequently, 20 μL of MTT solution at 5 mg mL^−1^ concentration was added and incubated for an additional 4 h. Following the solubilization of formazan crystals in 150 μL of DMSO, the absorbance of the plate was read using a microplate reader at a 570 nm wavelength. Cell viabilities were calculated with the following Equation (8)

(8) $$\%\text{ Cell viability}=\frac{{\text{OD}}_{\text{Test}}}{{\text{OD}}_{\text{Control}}}\times 100$$

##### Scratch Assay for Cell Migration and Wound Closure

An in vitro scratch assay was performed to evaluate the proliferation rate of mouse fibroblast (NIH‐3T3) upon treatment with the nanoflowers. Briefly, after growing NIH‐3T3 cells in complete DMEM media, they were seeded in a 96‐well plate at a cell density of 10^4^ cells/well while in their growth phase. Upon reaching full confluency, a vertical scratch was made at the center of each well using a 200 μL microtip, following which the cells were subjected to PBS (pH 7.4), free MB, CeZn NFs, CeZn@PPY NFs, and CeZn@PPY@MB NFs treatments. Two different sets of treatment regimens were followed: one without laser irradiation and another with laser irradiation, NIR light at an intensity of 1 W cm^−2^ for a time interval of 5 min. Images of the scratches were taken at 0, 4, 8, and 12 h time points, respectively, for each treatment regime using a fluorescence microscope (Leica, Germany). The scratch area was quantified using ImageJ software, and the % relative migration rate was evaluated using the following Equation (9)

(9) $$\%\text{ Relative migration rate=}\frac{{\text{OD}}_{\text{Test}}}{{\text{OD}}_{\text{Control}}}\times 100$$

##### Hemolysis Assay

To assess the hemocompatibility of CeZn@PPY@MB NFs, a hemolysis assay was conducted. Fresh whole blood was collected from healthy SD rats and centrifuged at 1500 rpm for 10 min to extract the red blood cells (RBCs). A 0.9% sodium chloride solution was used to wash the RBCs thrice. Positive control was achieved through a mixture of Triton‐X 100 with a 2% v/v suspension of RBCs. A negative control was achieved by mixing RBCs with normal saline. RBC suspension was mixed with different concentrations (2–1024 μg mL^−1^) of CeZn@PPY@MB NFs and was then incubated at 37 °C for 2 h. After incubation, supernatants of all groups were obtained by centrifugation at 1500 rpm for 10 min and transferred into 1.5 mL Eppendorf tubes. The absorbance of the supernatants was measured using a microplate reader at a 576 nm wavelength. RBCs were mixed with PBS or 0.9% NaCl (negative control) or DI water (positive control) to establish positive and negative control groups. Equation (10) was used to calculate the percentage of hemolysis

(10) $$\text{Hemolysis (}\%)=\frac{A_{\text{Test}}-A_{\text{Negative}}}{A_{\text{Positive}}-A_{\text{Negative}}}\times 100$$

where *A*_Test_, *A*_Negative_, and *A*_Positive_ are the absorbance values at 576 nm of experimental, negative, and positive groups, respectively.

##### HET‐CAM study

To evaluate the angiogenic potential of both laser‐treated and untreated NFs, using the 4‐day‐old fertile chicken eggs’ CAM. Eggs were disinfected with 70% ethanol on the outer shell. Drills and forceps were used to precisely excise a small section of the air sac side, which was then treated with different formulations. Control eggs were treated with sterile PBS. The dispersion of the nanoflowers in sterile PBS was plated and irradiated with a laser for 5 min, and the irradiated samples were then used to treat the CAM membrane. Parafilms were placed over the eggs and incubated at 37 °C and 60%–70% relative humidity for 8 h. Blood vessel density within the eggs of each treatment group was evaluated, and images were taken at a defined time point during the incubation process. Blood vessels in response to the different treatment groups were analyzed using ImageJ software and the percentage fold increase, respectively.

##### In Vivo *Diabetic Wound Healing Study*

Male Sprague Dawley (SD) rats (250–300 g) were procured and used for developing a diabetic rat wound model. The animal treatments were conducted per the rules set by The Committee for Control and Supervision of Experiments on Animals (CPCSEA), BITS‐Pilani Hyderabad, India. All procedures were approved by the Institutional Animal Ethics Committee, BITS‐Pilani Hyderabad campus (BITS‐HYD‐IAEC‐2024‐018). The experiments were performed under the supervision of a veterinarian. The animals were placed in cages and provided one week to adapt to their surroundings before initiating the experiment. Throughout the trial, they were supplied with free access to food and water. The optimal temperature of 20 ± 2 °C and a relative humidity varying from 45% to 60% were consistently maintained. In addition, a 12 h light and 12 h dark cycle was kept in the animal housing. The rats received a single intravenous (IV) dose of streptozotocin (50 mg kg^−1^) through their tail vein, and their blood glucose level was closely monitored till it exceeded 16.7 mM. Validating successful development of type‐1 diabetes mellitus (T1DM) in the rats, they were randomly assigned into five groups, namely Disease control, MB, uncoated CeZn NFs, CeZn@PPY NFs, and CeZn@PPY@MB NFs at *n* = 5, with and without NIR light irradiation (1.0 W cm^−2^, 5 min). A standard anesthetic protocol was followed. Circular skin incisions with a 10 mm diameter on the side of each rat were created using a sterile biopsy punch. Following this, 1000 μL of an MRSA suspension (1 × 10^7^ CFU mL^−1^) was applied. The above‐mentioned agents were administered at a dose volume of 100 μL, at the sub‐MIC dosage, topically on every alternate day for 14 days. Photographs were captured on days 0, 1, 3, 7, 11, and 14, and wound closure was measured using ImageJ software. The percentage of wound closure (WC%) was deduced using the following Equation (11)

(11) $$\text{WC}\%=\frac{W_{0}-W_{t}}{W_{0}}\times 100$$

where *W*_0_ denotes the wound area on the 0th day, while *W*_t_ denotes the wound area on the 1st, 3rd, 7th, 11th, or 14th day.

For histological analysis of wound healing, 5 μm wound sections underwent Hematoxylin–Eosin (H&E) and Masson's trichrome (MT) stainings. H&E staining involved fixation in 4% paraformaldehyde, clearing in xylene, and hydration/dehydration through a series of ethanol (100%, 90%, 70%, 50%, and 30%). Sections were stained with hematoxylin, underwent differentiation with acid alcohol, and were intensified using Scott's tap water. Eosin staining followed, with final dehydration through the ethanol series and clearing in xylene. Coverslips were mounted using a mounting medium. MT staining was also performed based on the manufacturer's protocol. Stained sections were imaged using fluorescence microscopy to assess re‐epithelialization, collagen deposition, and granulation tissue formation.

Immunofluorescence analysis was performed on cryosections fixed with 4% paraformaldehyde. After blocking with bovine serum albumin, sections were incubated with primary antibodies (IL‐1β, IL‐6, IL‐8, TNF‐α, CD‐44, Ki‐67, and DCFH‐DA) at 4 °C for 24 h. Following PBS washes, sections underwent incubation with fluorescently labeled secondary antibodies, nuclei were stained with DAPI, and coverslips were mounted with Fluoromount‐G for confocal microscopy. Glutathione levels in wound tissue were assessed using a colorimetric detection kit.

The amount of ceria and zinc present in the wound tissue was further analyzed and quantified from the wound tissue homogenates using ICP‐MS after performing metal extraction using ICP‐MS grade ultrapure nitric acid after 5 min of CeZn@PPY@MB NFs formulation addition and NIR irradiation.

##### In Vivo *Toxicity Assessment*

The evaluation of several blood biochemical parameters, such as aspartate aminotransferase (AST), alanine aminotransferase (ALT), creatinine, blood urea nitrogen (BUN), WBC count, RBC count, blood hemoglobin, blood platelet count, and percentage hematocrit, was assessed after retro‐orbital withdrawal of blood from the animals of each treatment group at the end of the 14th day and analyzed with the respective kits. Furthermore, H&E staining of all the major organs, namely the heart, lungs, liver, spleen, and kidneys, was performed and analyzed.

##### Statistical Analysis

The data were expressed as mean ± SD (*n* ≥ 3). Data analysis was performed using ImageJ (version 1.8.0) and Origin 2024. Data normalization, *n* for groups, *p* values for probability, and applied statistical tests for each dataset are described within the figure legends. Group differences were evaluated using one‐way or two‐way analysis of variance (ANOVA) along with Tukey's post‐test, accompanied by *p*‐values.

## Supporting Information

Supporting Information is available from the Wiley Online Library or from the author.

## Conflict of Interest

The authors declare no conflict of interest.

## Supporting information

Supplementary Material

## Data Availability

The data that support the findings of this study are available from the corresponding author upon reasonable request.

## References

[1] Y. Yu , R. Tian , Y. Zhao , X. Qin , L. Hu , J. J. Zou , Y. W. Yang , J. Tian , Adv. Healthc. Mater. 2023, 12, 202201651. 10.1002/adhm.202201651 36168853

[2] S. MacNeil , Nature 2007, 445, 874. 17314974 10.1038/nature05664

[3] W. Ahmed , S. Li , M. Liang , P. Peng , W. Muhammad , Q. Wang , C. Gao , Biomater. Adv. 2025, 173, 214289. 40158270 10.1016/j.bioadv.2025.214289

[4] S. De , A. Ghosh , D. Mandal , K. Sarkar , A. P. Samanta , M. Basak , A. Saha , D. Bhattacharya , S. Nandi , J. Sarkar , M. Mandal , K. Acharya , P. Ghosh , D. Chattopadhyay , ACS Appl. Bio. Mater. 2024, 7, 6414. 10.1021/acsabm.4c00551 39287553

[5] H. Wu , F. Li , W. Shao , J. Gao , D. Ling , ACS Cent. Sci. 2019, 5, 477. 30937375 10.1021/acscentsci.8b00850 PMC6439452

[6] A. Malik , Z. Mohammad , J. Ahmad , Diabetes Metab. Syndr.: Clin. Res. Rev. 2013, 7, 101. 10.1016/j.dsx.2013.02.006 23680250

[7] M. Kumar , S. Mahmood , S. Chopra , A. Bhatia , Int. J. Biol. Macromol. 2024, 267, 131335. 38604431 10.1016/j.ijbiomac.2024.131335

[8] Y. Yu , R. Tian , Y. Zhao , X. Qin , L. Hu , J. J. Zou , Y. W. Yang , J. Tian , Adv. Healthc. Mater. 2023, 12, 2201651. 10.1002/adhm.202201651 36168853

[9] Z. Y. Li , X. J. Zhang , Y. M. Gao , Y. Song , M. X. Sands , S. B. Zhou , Q. F. Li , J. Zhang , Adv. Healthc. Mater. 2023, 12, 2202770. 10.1002/adhm.202202770 36864695

[10] Q. Han , J. W. Lau , T. C. Do , Z. Zhang , B. Xing , ACS Appl. Bio. Mater. 2021, 4, 3937. 10.1021/acsabm.0c01341 35006816

[11] X. Wang , W. Shi , Y. Jin , Z. Li , T. Deng , T. Su , A. Zheng , L. Cao , J. Nanobiotechnol. 2025, 23, 40. 10.1186/s12951-025-03126-2 PMC11756032 39849558

[12] Y. Xing , J. Zhang , F. Chen , J. Liu , K. Cai , Nanoscale 2017, 9, 8781. 28621774 10.1039/c7nr01857f

[13] Q. Gao , X. Zhang , W. Yin , D. Ma , C. Xie , L. Zheng , X. Dong , L. Mei , J. Yu , C. Wang , Z. Gu , Y. Zhao , Small 2018, 14, 1802290. 10.1002/smll.201802290 30307703

[14] C. Hu , F. Zhang , L. Long , Q. Kong , R. Luo , Y. Wang , J. Controlled Release 2020, 324, 204. 10.1016/j.jconrel.2020.05.010 32389779

[15] X. Jin , J. Shan , J. Zhao , T. Wang , W. Zhang , S. Yang , H. Qian , L. Cheng , X. L. Chen , X. Wang , Acta Biomater. 2024, 173, 403. 10.1016/j.actbio.2023.10.028 39492500

[16] G. Shu , D. Xu , S. Xie , L. J. Chang , X. Liu , J. Yang , Y. Li , X. Wang , Appl. Surf. Sci. 2023, 611, 155727.

[17] W. F. Chai , K. S. Tang , J. Trace Elem. Med. Biol. 2021, 66, 126742. 33773280 10.1016/j.jtemb.2021.126742

[18] Z. Wang , Y. Zhang , S. Chen , Y. Qu , M. Tang , W. Wang , W. Li , L. Gu , Chem. Eng. J. 2024, 485, 149842.

[19] A. R. Siddiqui , J. M. Bernstein , Clin. Dermatol. 2010, 28, 519. 20797512 10.1016/j.clindermatol.2010.03.009

[20] X. Geng , K. Liu , J. Wang , X. Su , Y. Shi , L. Zhao , Int. J. Nanomed. 2023, 18, 3339. 10.2147/IJN.S399933 PMC10289105 37361387

[21] S. Patel , S. Srivastava , M. R. Singh , D. Singh , Biomed. Pharmacother. 2019, 112, 108615. 30784919 10.1016/j.biopha.2019.108615

[22] X. Xiao , F. Zhao , D. B. DuBois , Q. Liu , Y. L. Zhang , Q. Yao , G. J. Zhang , S. Chen , ACS Biomater. Sci. Eng. 2024, 10, 4195. 38752382 10.1021/acsbiomaterials.4c00470

[23] M. Li , H. Jafari , O. V. Okoro , L. Nie , A. Shavandi , Cell Biomater. 2025, 1, 100049.

[24] S. Khattak , I. Ullah , M. Sohail , M. U. Akbar , M. A. Rauf , S. Ullah , J. Shen , H. T. Xu , Aggregate 2024, 6, e688.

[25] F. Dai , J. Zhang , F. Chen , X. Chen , C. J. Lee , H. Liang , L. Zhao , H. Tan , Adv. Sci. 2024, 11, 2408783. 10.1002/advs.202408783 PMC11633493 39435670

[26] X. Li , X. Jing , Z. Yu , Y. Huang , Adv. Healthc. Mater. 2023, 12, 2300375. 10.1002/adhm.202300375 37141030

[27] N. Guo , F. Cang , Z. Wang , T. T. Zhao , X. R. Song , S. Farris , Y. Y. Li , Y. J. Fu , Mater. Sci. Eng. C 2021, 126, 112143. 10.1016/j.msec.2021.112143 34082954

[28] X. He , X. Wu , K. Wang , B. Shi , L. Hai , Biomaterials 2009, 30, 5601. 19595455 10.1016/j.biomaterials.2009.06.030

[29] N. A. Chopan , H. T. N. Chishti , Mater. Today Chem. 2023, 32, 101643.

[30] P. Wang , L. Peng , J. Lin , Y. Li , Q. Luo , S. Jiang , H. Tian , Y. Zhang , X. Liu , J. Liu , Chem. Eng. J. 2021, 415, 128901.

[31] Y. Shen , C. Nie , T. Pan , W. Zhang , H. Yang , Y. Ye , X. Wang , Acta Biomater. 2023, 168, 580. 37451659 10.1016/j.actbio.2023.07.006

[32] W. Guo , M. Zhang , Z. Lou , M. Zhou , P. Wang , H. Wei , ChemCatChem 2019, 11, 737.

[33] P. Wang , F. Meng , C. Gao , W. Xie , J. Wang , A. Li , J. Mater. Sci.: Mater. Electron. 2018, 29, 11482.

[34] J. Chauhan Rajiv Gandhi , B. Jyotsna Chauhan , HOD Nanotechnology department RGPV, Synthesis and Characterization Zinc, Nickel AND silver with different doping Synthesis and Characterization Zinc, Nickel AND silver with different doping Synthesis and characterization of Mn/Fe/Co/Ni/Cu doped ZnO 2019, https://www.researchgate.net/publication/352992689.

[35] A. Jana , N. R. Bandyopadhyay , P. Sujatha Devi , Solid State Sci. 2011, 13, 1633.

[36] U. Das , T. Dey , P. Chatterjee , A. K. Mukherjee , Powder Diffr. 2017, 32, 86.

[37] Z. Chu , X. Liu , T. Zhao , D. Jiang , J. Zhao , X. Dong , K. W. K. Yeung , X. Liu , Y. Liao , L. Ouyang , Biomaterials 2025, 315, 122964. 39550986 10.1016/j.biomaterials.2024.122964

[38] Y. Deng , X. Ouyang , J. Sun , X. Shi , Y. Li , Y. K. Chan , W. Yang , S. Peng , Bioact. Mater. 2023, 25, 748. 37056260 10.1016/j.bioactmat.2022.07.003 PMC10087611

[39] T. T. Vy Phan , S. Bharathiraja , V. T. Nguyen , M. S. Moorthy , P. Manivasagan , K. D. Lee , J. Oh , RSC Adv. 2017, 7, 35027.

[40] M. Yin , Z. Li , E. Ju , Z. Wang , K. Dong , J. Ren , X. Qu , Chem. Commun. 2014, 50, 10488. 10.1039/c4cc04584j 25068798

[41] J. Yu , C. H. Hsu , C. C. Huang , P. Y. Chang , ACS Appl. Mater. Interfaces 2015, 7, 432. 25494339 10.1021/am5064298

[42] M. Li , X. Liu , L. Tan , Z. Cui , X. Yang , Z. Li , Y. Zheng , K. W. K. Yeung , P. K. Chu , S. Wu , Biomater. Sci. 2018, 6, 2110. 29882566 10.1039/c8bm00499d

[43] Y. Ju , X. Liu , X. Ye , M. Dai , B. Fang , X. Shen , L. Liu , ACS Appl. Nano Mater. 2023, 6, 13792.

[44] Y. J. Wei , H. Chen , Z. W. Zhou , C. X. Liu , C. X. Cai , J. Li , X. Q. Yu , J. Zhang , Y. H. Liu , N. Wang , Small 2024, 20, 2403679. 10.1002/smll.202403679 39240068

[45] L. He , Z. Li , M. Gu , Y. Li , C. Yi , M. Jiang , X. Yu , L. Xu , Adv. Sci. 2024, 11, 2406681. 10.1002/advs.202406681 PMC11516101 39225540

[46] J. Yang , Z. Chu , Y. Jiang , W. Zheng , J. Sun , L. Xu , Y. Ma , W. Wang , M. Shao , H. Qian , Adv. Healthc. Mater. 2023, 12, 2300725. 10.1002/adhm.202300725 37086396

[47] S. Li , E. Jiaoting , X. Zhao , R. Xie , J. Wu , L. Feng , H. Ding , F. He , P. Yang , Adv. Mater. 2025, 37, 2417198. 10.1002/adma.202417198 40123217

[48] C. Liu , X. Xu , Y. Chen , M. Yin , E. Mäkilä , W. Zhou , W. Su , H. Zhang , Small 2024, 20, 2307794. 10.1002/smll.202307794 38168483

[49] A. C. Afonso , D. Oliveira , M. J. Saavedra , A. Borges , M. Simões , Int. J. Mol. Sci. 2021, 22, 8278. 34361044 10.3390/ijms22158278 PMC8347492

[50] W. Wentao , Z. Tao , S. Bulei , Z. Tongchang , Z. Qicheng , W. Fan , Z. Ninglin , S. Jian , Z. Ming , S. Yi , Appl. Mater. Today 2019, 17, 36.

[51] B. Zhao , H. Wang , W. Dong , S. Cheng , H. Li , J. Tan , J. Zhou , W. He , L. Li , J. Zhang , G. Luo , W. Qian , J. Nanobiotechnol. 2020, 18, 59. 10.1186/s12951-020-00614-5 PMC7158002 32293461

[52] X. Qi , E. Grafskaia , Z. Yu , N. Shen , E. Fedina , A. Masyutin , M. Erokhina , M. Lepoitevin , V. Lazarev , N. Zigangirova , C. Serre , M. Durymanov , ACS Infect. Dis. 2023, 9, 1558. 37477515 10.1021/acsinfecdis.3c00131

[53] L. T. Yang , W. J. Wang , W. T. Huang , L. C. Wang , M. C. Hsu , C. D. Kan , C. Y. Huang , T. W. Wong , W. P. Li , ACS Appl. Mater. Interfaces 2024, 16, 12018. 38394675 10.1021/acsami.3c17424 PMC10921379

[54] J. Sun , Y. Zhang , J. Su , T. Dai , J. Chen , L. Zhang , H. Wang , W. Liu , M. Huang , Z. Chen , Dyes Pigm. 2020, 179, 108392.

[55] Z. Li , X. Zhu , J. Xiao , W. Lu , D. Gan , J. Shen , X. Jiang , M. Wang , Chem. Eng. J. 2025, 513, 163016.

[56] R. Boltes Cecatto , L. Siqueira de Magalhães , M. Fernanda Setúbal Destro Rodrigues , C. Pavani , A. Lino‐dos‐Santos‐Franco , M. Teixeira Gomes , D. Fátima Teixeira Silva , Photodiagn. Photodyn. Ther. 2020, 31, 101828. 10.1016/j.pdpdt.2020.101828 32473398

[57] D. Liu , L. Liu , L. Yao , X. Peng , Y. Li , T. Jiang , H. Kuang , J. Drug Deliv. Sci. Technol. 2020, 55, 101364.

[58] S. Brouwer , M. G. Jespersen , C. L. Y. Ong , D. M. P. De Oliveira , B. Keller , A. J. Cork , K. Y. Djoko , M. R. Davies , M. J. Walker , MBio 2022, 13, e00676‐22. 35467425 10.1128/mbio.00676-22 PMC9239160

[59] X. Dai , Y. Zhao , Y. Yu , X. Chen , X. Wei , X. Zhang , C. Li , ACS Appl. Mater. Interfaces 2017, 9, 30470. 28832120 10.1021/acsami.7b09638

[60] T. Hirsch , M. Spielmann , B. Zuhaili , T. Koehler , M. Fossum , H. U. Steinau , F. Yao , L. Steinstraesser , A. B. Onderdonk , E. Eriksson , BMC Surg. 2008, 8, 5. 18312623 10.1186/1471-2482-8-5 PMC2276479

[61] X. Hu , Y. Y. Huang , Y. Wang , X. Wang , M. R. Hamblin , Front. Microbiol. 2018, 9, 1299. 29997579 10.3389/fmicb.2018.01299 PMC6030385

[62] Z. Sedarat , A. W. Taylor‐Robinson , Pathogens 2022, 11, 388. 35456063 10.3390/pathogens11040388 PMC9027693

[63] Y. Sun , H. Qin , Z. Yan , C. Zhao , J. Ren , X. Qu , Adv. Funct. Mater. 2020, 30, 1909740.

[64] N. Kathiresan , D. Prabu , H. Kasilingam , P. Sangavi , V. R. Arumugam , L. Kulanthaivel , Biocatal. Agric. Biotechnol. 2024, 62, 103442.

[65] M. Rohde , Microbiol. Spectr. 2019, 7, 10. 10.1128/microbiolspec.gpp3-0044-2018 PMC11086966 31124431

[66] Z. Xu , Y. Liang , S. Lin , D. Chen , B. Li , L. Li , Y. Deng , Curr. Microbiol. 2016, 73, 474. 27324342 10.1007/s00284-016-1081-1

[67] R. Augustine , A. A. Zahid , A. Hasan , Y. B. Dalvi , J. Jacob , ACS Biomater. Sci. Eng. 2021, 7, 279. 33320529 10.1021/acsbiomaterials.0c01138

[68] A. Hassan , D. Elebeedy , E. R. Matar , A. Fahmy Mohamed Elsayed , A. I. Abd El Maksoud , Front. Pharmacol. 2021, 12, 661217. 34721007 10.3389/fphar.2021.661217 PMC8552110

[69] X. Zhao , L. Chang , Y. Hu , S. Xu , Z. Liang , X. Ren , X. Mei , Z. Chen , ACS Appl. Mater. Interfaces 2022, 14, 18194. 35412791 10.1021/acsami.2c03001

[70] X. Xu , H. Mao , Y. Wu , S. Liu , J. Liu , Q. Li , M. Yang , J. Zhu , S. Zou , F. Du , J. Nanobiotechnol. 2022, 20, 297. 10.1186/s12951-022-01507-5 PMC9214988 35733214

[71] Y. Feng , L. Su , Z. Zhang , Y. Chen , M. R. Younis , D. Chen , J. Xu , C. Dong , Y. Que , C. Fan , Y. Jiao , H. Zhu , J. Chang , Z. Dong , C. Yang , ACS Appl. Mater. Interfaces 2024, 16, 95. 38157482 10.1021/acsami.3c12997

[72] S. Kamalipooya , S. Fahimirad , H. Abtahi , M. Golmohammadi , M. Satari , M. Dadashpour , D. Nasrabadi , Int. J. Pharm. 2024, 653, 123880. 38350498 10.1016/j.ijpharm.2024.123880

[73] D. Bagchi , V. S. S. Rathnam , P. Lemmens , I. Banerjee , S. K. Pal , ACS Omega 2018, 3, 10877. 30320255 10.1021/acsomega.8b00716 PMC6173506

[74] Z. Yuan , B. Tao , Y. He , C. Mu , G. Liu , J. Zhang , Q. Liao , P. Liu , K. Cai , Biomaterials 2019, 223, 119479. 31520885 10.1016/j.biomaterials.2019.119479

[75] S. Khattak , I. Ullah , M. Sohail , M. U. Akbar , M. A. Rauf , S. Ullah , J. Shen , H. T. Xu , Aggregate 2024, 8, e688.

[76] Z. Li , Y. Zhao , H. Huang , C. Zhang , H. Liu , Z. Wang , M. Yi , N. Xie , Y. Shen , X. Ren , J. Wang , J. Wang , Adv. Healthc. Mater. 2022, 11, 2201524. 10.1002/adhm.202201524 36100580

[77] Y. Xue , F. Yang , Y. He , F. Wang , D. Xia , Y. Liu , Adv. Healthc. Mater. 2025, 14, 2402236. 10.1002/adhm.202402236 39780538

[78] Y. Li , R. Fu , Z. Duan , C. Zhu , D. Fan , Small 2022, 18, 2200165. 10.1002/smll.202200165 35373522

[79] D. Chao , Q. Dong , Z. Yu , D. Qi , M. Li , L. Xu , L. Liu , Y. Fang , S. Dong , J. Am. Chem. Soc. 2022, 144, 23438. 36512736 10.1021/jacs.2c09663

[80] J. R. do Carmo Neto , P. I. R. Franco , Y. L. L. Braga , J. F. de Oliveira , H. F. Perini , L. F. D. Albuquerque , D. B. Martins , F. R. Helmo , A. A. Andrade , M. P. Miguel , M. R. N. Celes , T. L. Rocha , A. C. Almeida Silva , J. R. Machado , M. V. da Silva , J. Funct. Biomater. 2024, 15, 51. 38535244 10.3390/jfb15030051 PMC10971593

[81] Z. Chen , W. Liao , X. Ni , Chem. Eng. J. 2017, 327, 1198.

