# Supplementary material for: Bimetallic Nanozymes/Polypyrrole/Methylene Blue Platform for Photothermal and Catalytic Biofilm Disruption and Angiogenesis Enhancement in Diabetic Wound Healing
Source: Small Sci. 2026 Feb 3;6(2):e202500445. doi: 10.1002/smsc.202500445 (PMC12928003; doi:10.1002/smsc.202500445)
Supplement: Supplementary file 1 — Supplementary Material [file SMSC-6-e202500445-s001.pdf]

Supplementary Information

Bimetallic Nanozymes/Polypyrrole/Methylene Blue Platform for Photothermal and Catalytic Biofilm Disruption and Angiogenesis Enhancement in Diabetic Wound Healing

Prafful P. Kothari[1], Tonmoy Banerjee[1], Balaram Ghosh[1], Swati Biswas[1*]

[1]Nanomedicine Research Laboratory, Department of Pharmacy, Birla Institute of Technology & Science-Pilani, Hyderabad Campus, Medchal, Hyderabad-500078, Telangana, India.

*Corresponding authors

Swati Biswas, Ph.D.

Professor

Department of Pharmacy

BITS-Pilani, Hyderabad Campus

Medchal, Hyderabad-500078

Telangana, India

Email: swati.biswas@hyderabad.bits-pilani.ac.in

Phone: (+91)-406-6303630

Fax: 040-6630-399

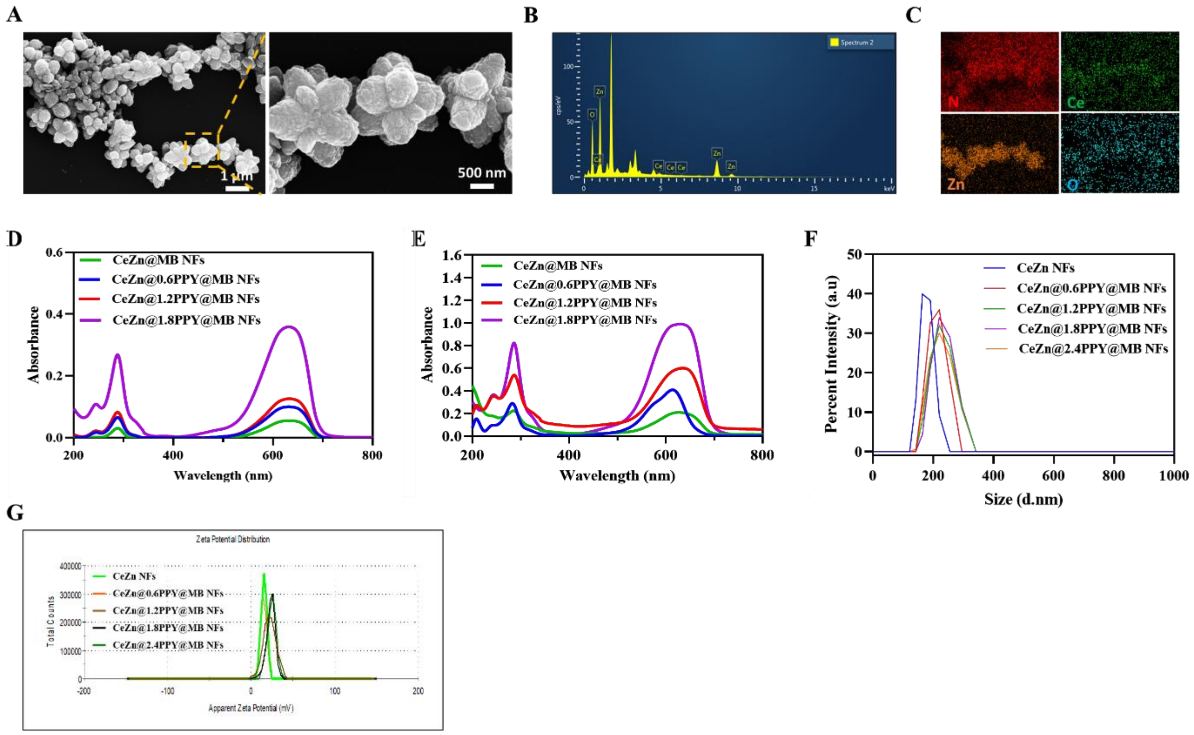

Figure S1. A-C) SEM images, EDX, and elemental mapping of CeZn@PPY NFs. D, E) UV-Visible spectrum of dissociated MB from CeZn@MB NFs, CeZn@0.6PPY@MB NFs, CeZn@1.2PPY@MB NFs, and CeZn@1.8PPY@MB NFs, without and with NIR (808 nm) irradiation in methanol. F) Particle size graph of CeZn@MB NFs, CeZn@0.6PPY@MB NFs, CeZn@1.2PPY@MB NFs, and CeZn@1.8PPY@MB NFs. G) Zeta potential graph of CeZn@MB NFs, CeZn@0.6PPY@MB NFs, CeZn@1.2PPY@MB NFs, and CeZn@1.8PPY@MB NFs.

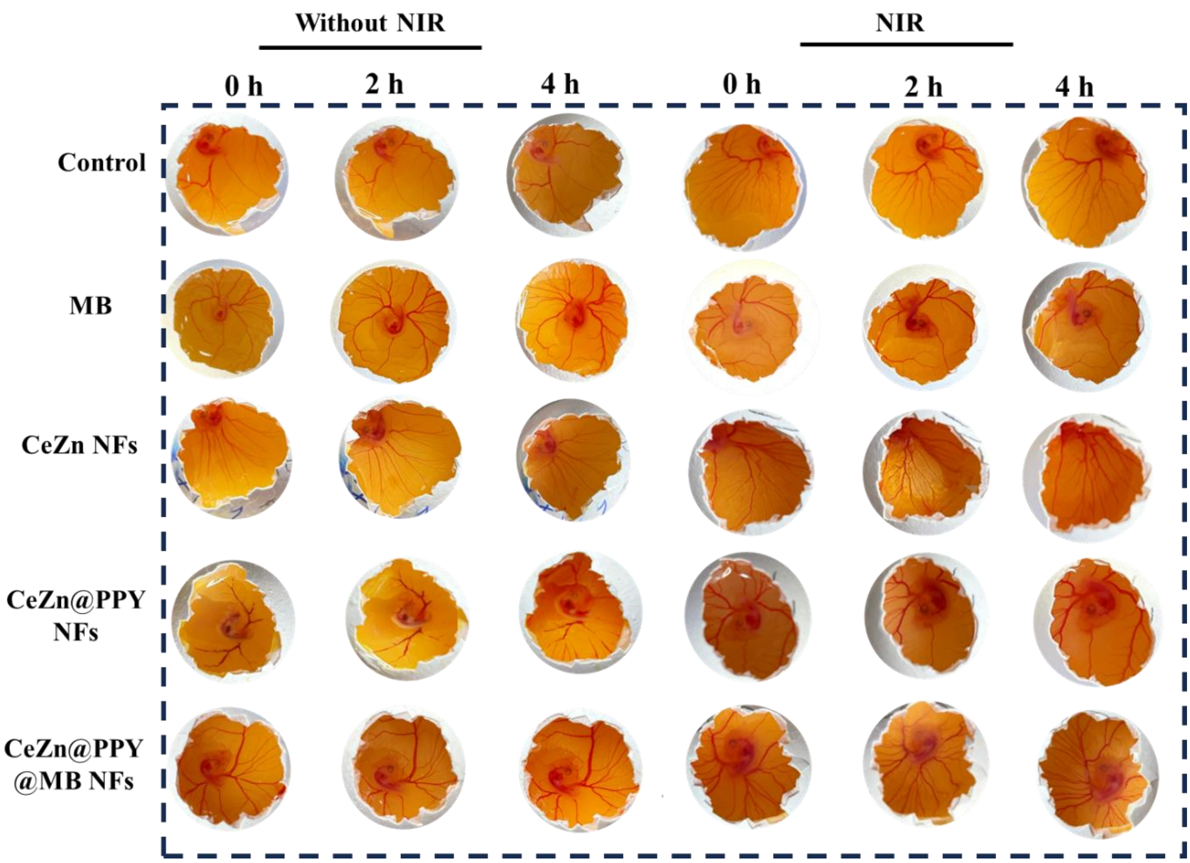

Figure S2. Images showing the effect of free MB, CeZn NFs, CeZn@PPY NFs, and CeZn@PPY@MB NFs on angiogenesis in CAM of fertilized hen eggs compared to the PBS control, without or with NIR (808 nm, 1 W cm^{-2}, 5 minutes) for 2 and 4 h time period.

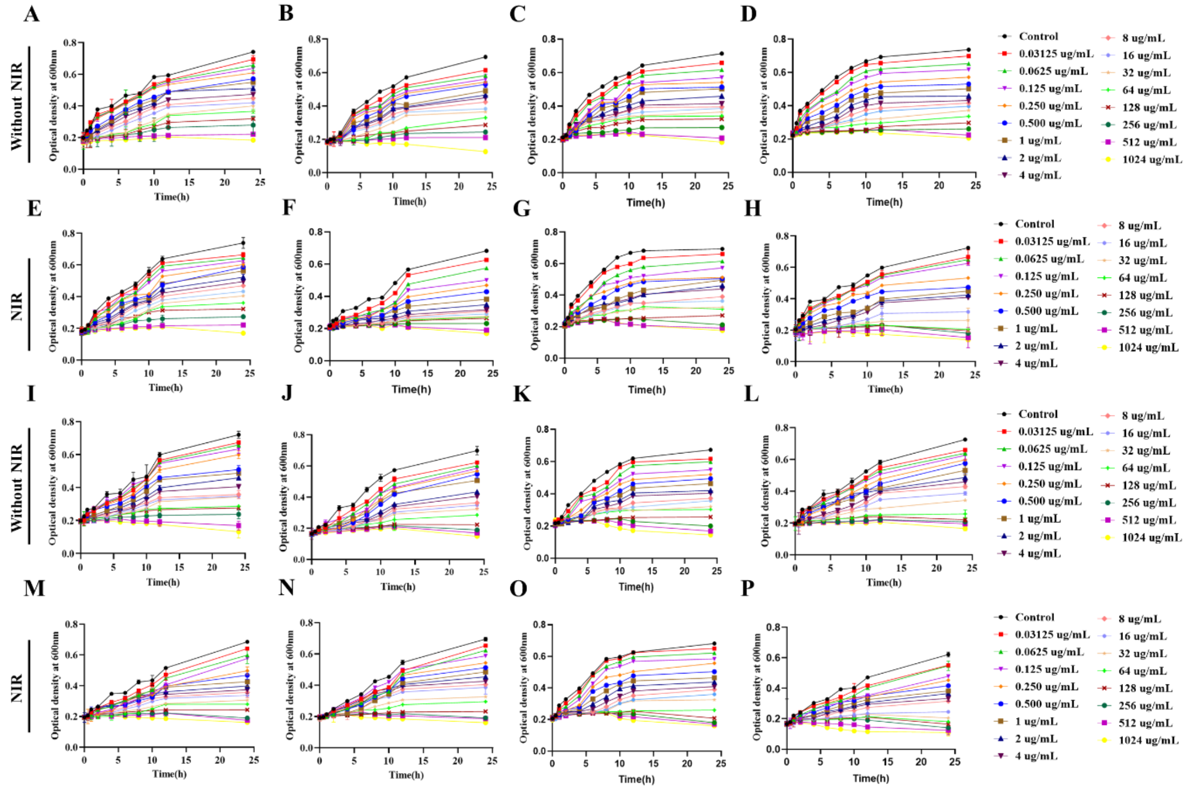

Figure S3. A-H) Minimum inhibitory concentration of free MB, CeZn NFs, CeZn@PPY NFs, and CeZn@PPY@MB NFs against methicillin-resistant *Staphylococcus aureus* (MRSA) without or with NIR irradiation (808 nm, 1 W cm^{-2}, 5 minutes), respectively (n=3). I-P) Minimum inhibitory concentration of free MB, CeZn NFs, CeZn@PPY NFs, and CeZn@PPY@MB NFs against sensitive *Staphylococcus aureus* (SA) without or with NIR irradiation (808 nm, 1 W cm^{-2}, 5 minutes), respectively (n=3).

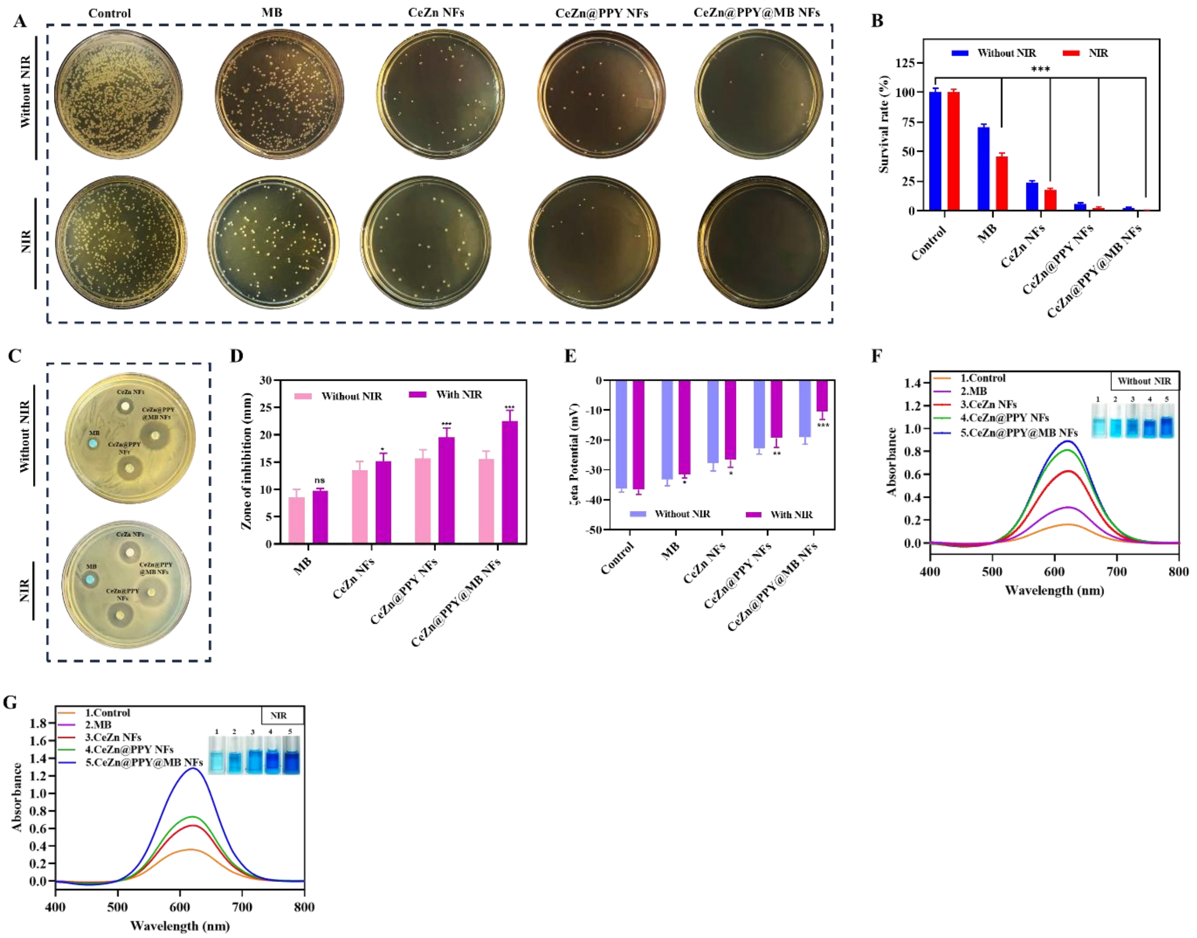

Figure S4. In vitro antibacterial effects of free MB, CeZn NFs, CeZn@PPY NFs, and CeZn@PPY@MB NFs treatments against sensitive *Staphylococcus aureus* (SA) without and with NIR (808nm, 1 W cm^{-2}, 5 minutes) irradiation, compared to PBS (pH-7.4) treated control. A) Photographs of bacterial survival. B) Quantitative analysis of bacterial survival. C) Photographs representing the diameter of the zone of inhibition around 6 mm discs infused with various nanoflower treatments. D) Quantitative analysis of the diameter of the zone of inhibition with various nanoflower treatments. E) Zeta potential of bacterial cultures treated with various nanoflower treatments. F, G) Protein leakage assay of bacterial cultures treated with various nanoflower treatments using Bradford reagent, without and with NIR irradiation, respectively, (n=3). All data represented as mean ± SD (*p<0.05, **p<0.01, ***p<0.001).

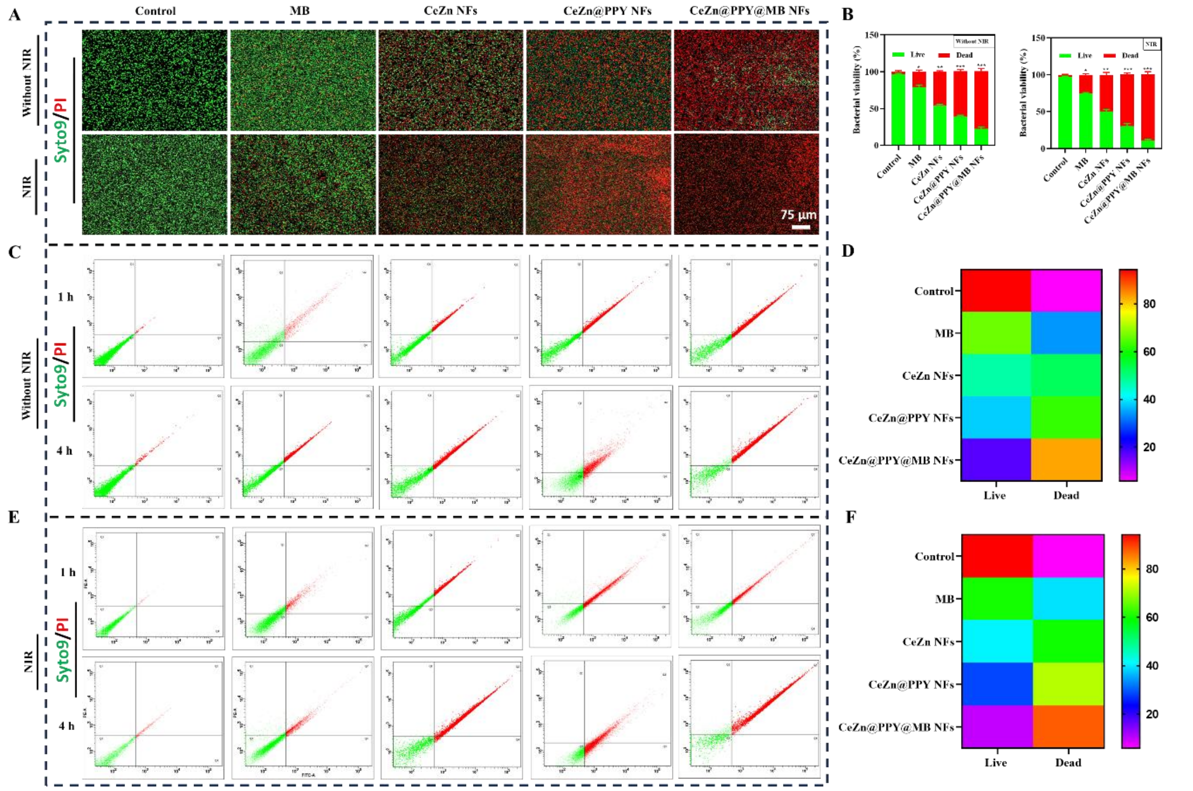

Figure S5. In vitro Syto9 and PI stained live/dead assay (green-live, red-dead) of sensitive *Staphylococcus aureus* (SA) incurring treatments due to free MB, CeZn NFs, CeZn@PPY NFs, and CeZn@PPY@MB NFs without and with NIR (808nm, 1 W cm^{-2}, 5 minutes) irradiation, compared to PBS (pH-7.4) treated control. A) Merged images showing relative live/dead bacterial populations. B) Quantitative analysis of relative live/dead bacterial cell populations. C, E) The graphical representation of live and dead cell populations using flow cytometric (FACS) dot plots, without and with NIR irradiation, respectively. D, F) Heat maps representing relative live and dead cell populations in Q1, Q3, Q2, and Q4 quadrants of FACS-generated dot plots, without and with NIR treatments, respectively, (n=3). All data represented as mean ± SD (*p<0.05, **p<0.01, ***p<0.001).

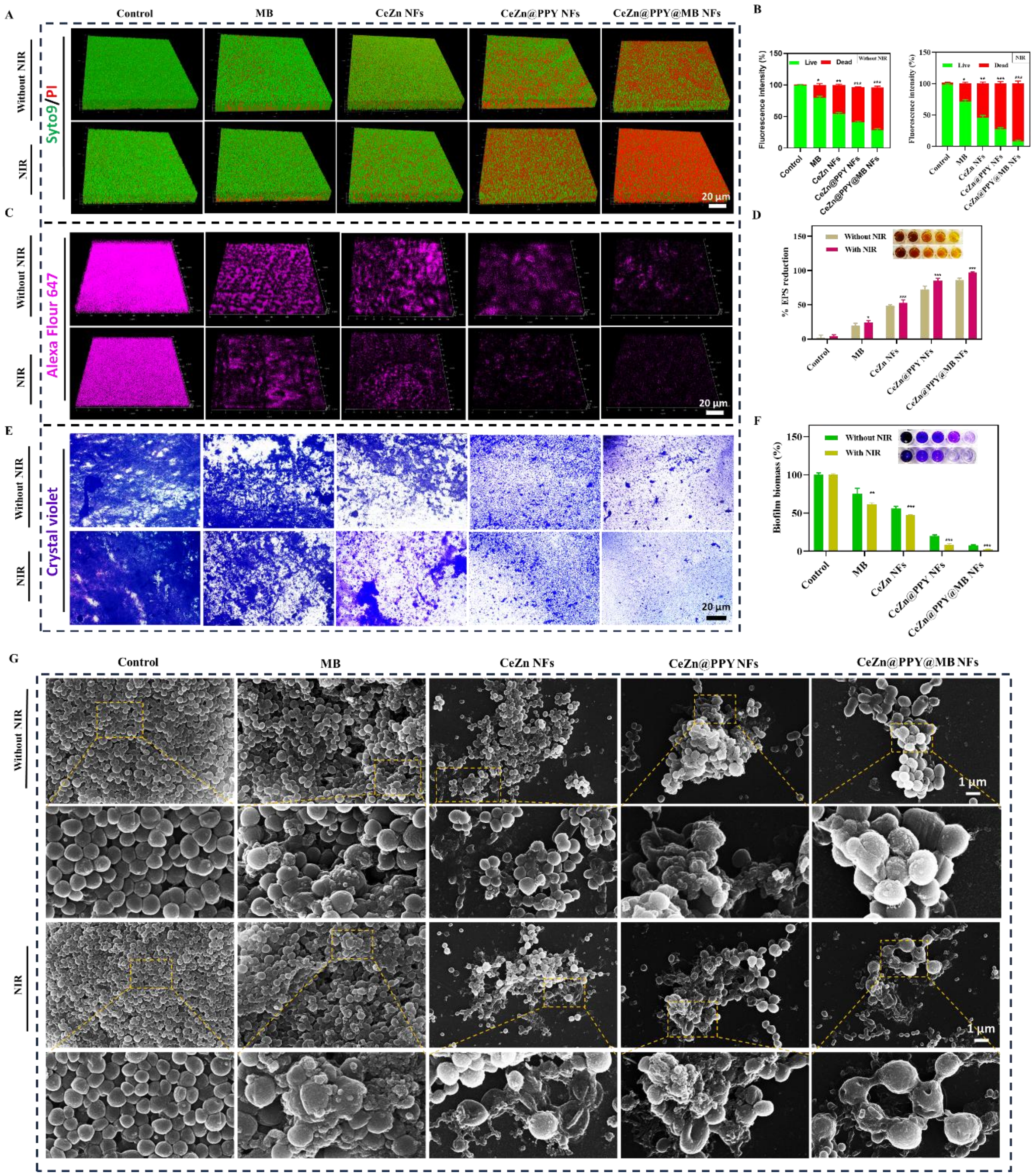

Figure S6. Anti-biofilm activity effects of free MB, CeZn NFs, CeZn@PPY NFs, and CeZn@PPY@MB NFs treatments against sensitive *Staphylococcus aureus* (MRSA) without and with NIR (808nm, 1 W cm^{-2}, 5 minutes) irradiation, compared to PBS (pH-7.4) treated control. A) 3D images of Syto 9/PI treated biofilms, visualized through confocal microscope. Scale = 20 µm. B) Quantitative analysis of relative live/dead bacterial cell populations. C) 3D images of Alexa 647 treated EPS expressing biofilms, visualized through confocal microscope. Scale = 20 µm. D) Percentage of EPS expressing biofilm analysis after various treatments by phenol sulfuric acid method. E) Bright field images of crystal violet-stained bacterial biofilms. F) Percentage of biofilm biomass analysis due to various treatments,

(n=3). G) Biofilm disruption images due to various nanoflower treatments, free MB, and PBS treated control, by SEM (n=3). All data represented as mean ± SD (*p<0.05, **p<0.01, ***p<0.001).

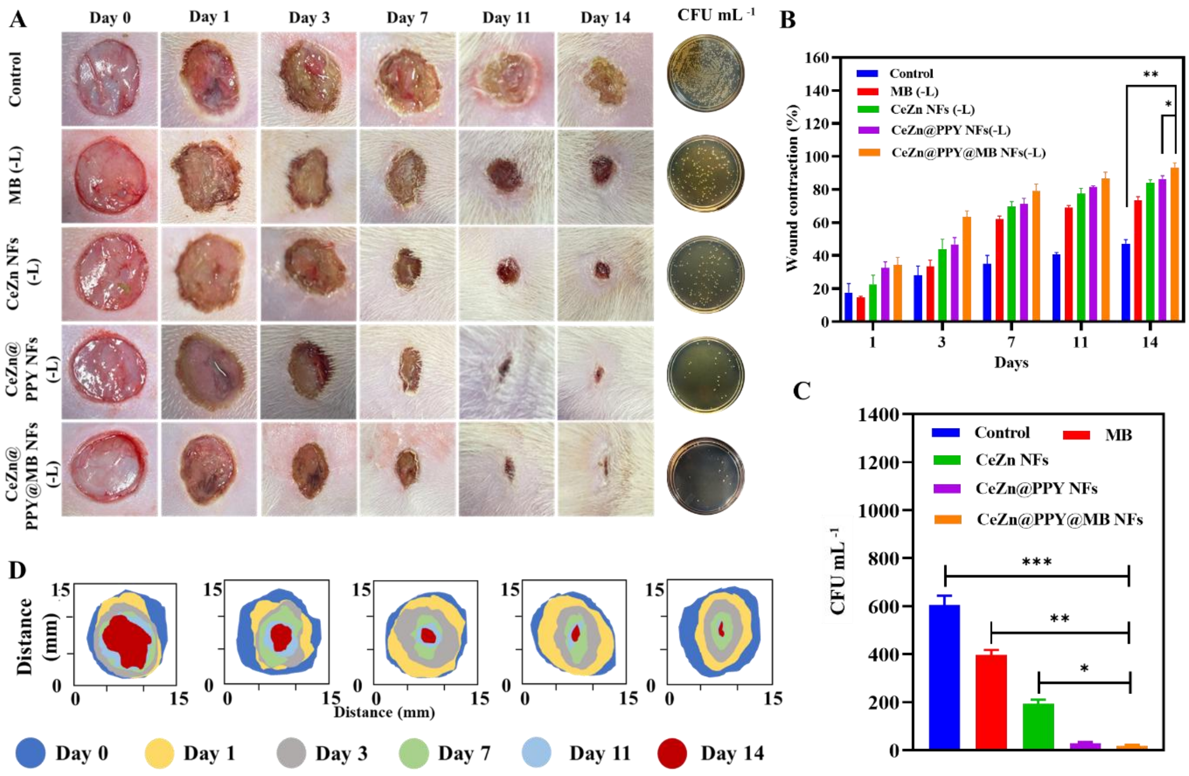

Figure S7. In vivo wound healing efficacy of CeZn NFs, CeZn@PPY NFs, and CeZn@PPY@MB NFs in diabetic rats compared to PBS (pH-7.4) treated control and free MB, without NIR irradiation. A) Images of wounds on different days of treatment, followed by images of MRSA colonies formed after plating the 14th-day wound tissue homogenate. B) Relative wound closure efficacy analysis of different treatments on various days of treatment. C) Analysis of MRSA colony counts formed after plating the 14th-day wound tissue homogenate. D) Trace images of wound closure on days 0, 1, 3, 7, 11, and 14, (n=5). All data represented as mean ± SD (*p<0.05, **p<0.01, ***p<0.001).

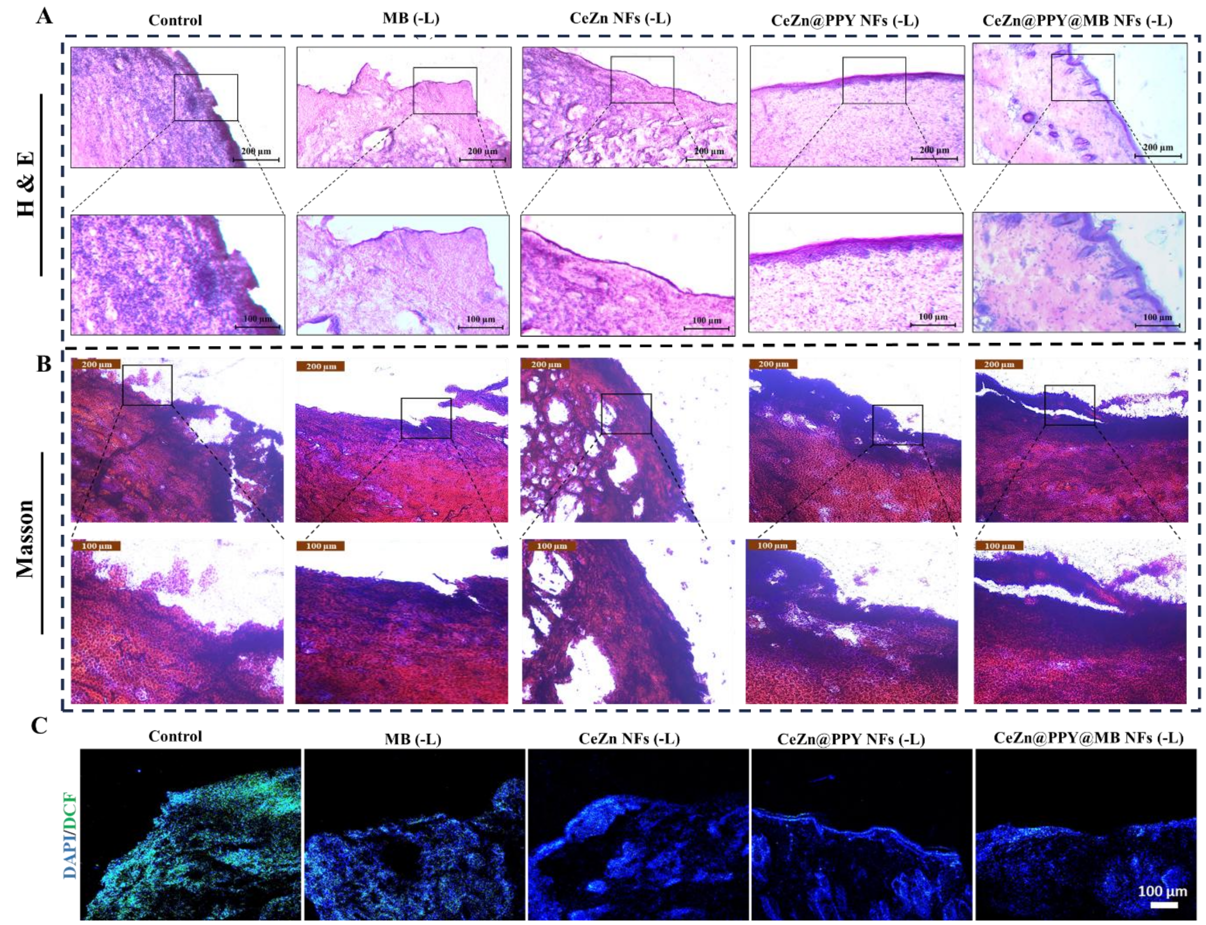

Figure S8. The in vivo efficiency of wound healing of CeZn NFs, CeZn@PPY NFs, and CeZn@PPY@MB NFs, without NIR irradiation, was evaluated in diabetic rats compared with PBS (pH 7.4) through histological evaluation of the wound tissues at day 14. A) H&E staining, and B) Masson's staining of wound tissue on day 14. C) ROS content of wound tissue at day 14, (n=3). All data represented as mean ± SD (*p<0.05, **p<0.01, ***p<0.001).

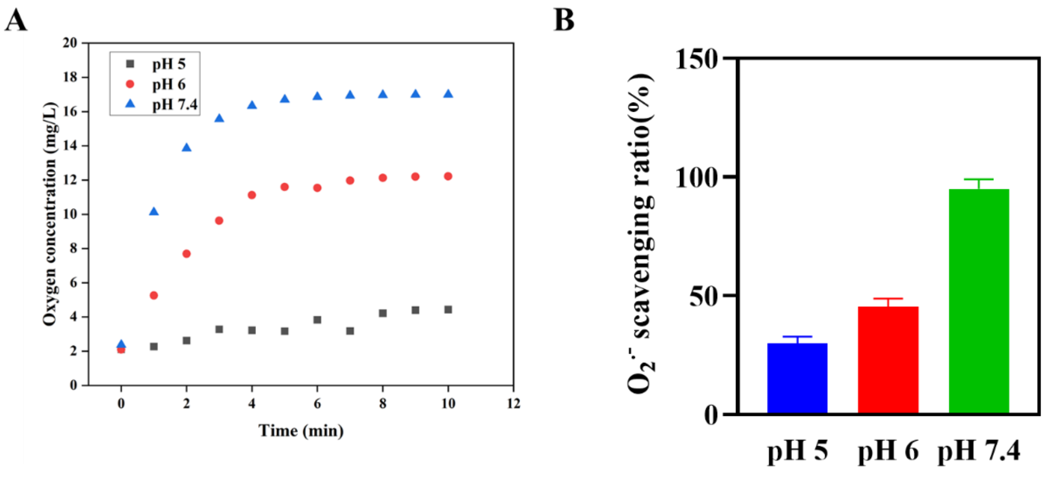

Figure S9. A-B) pH dependent CAT- and SOD-like activities of CeZn@PPY@MB NFs.

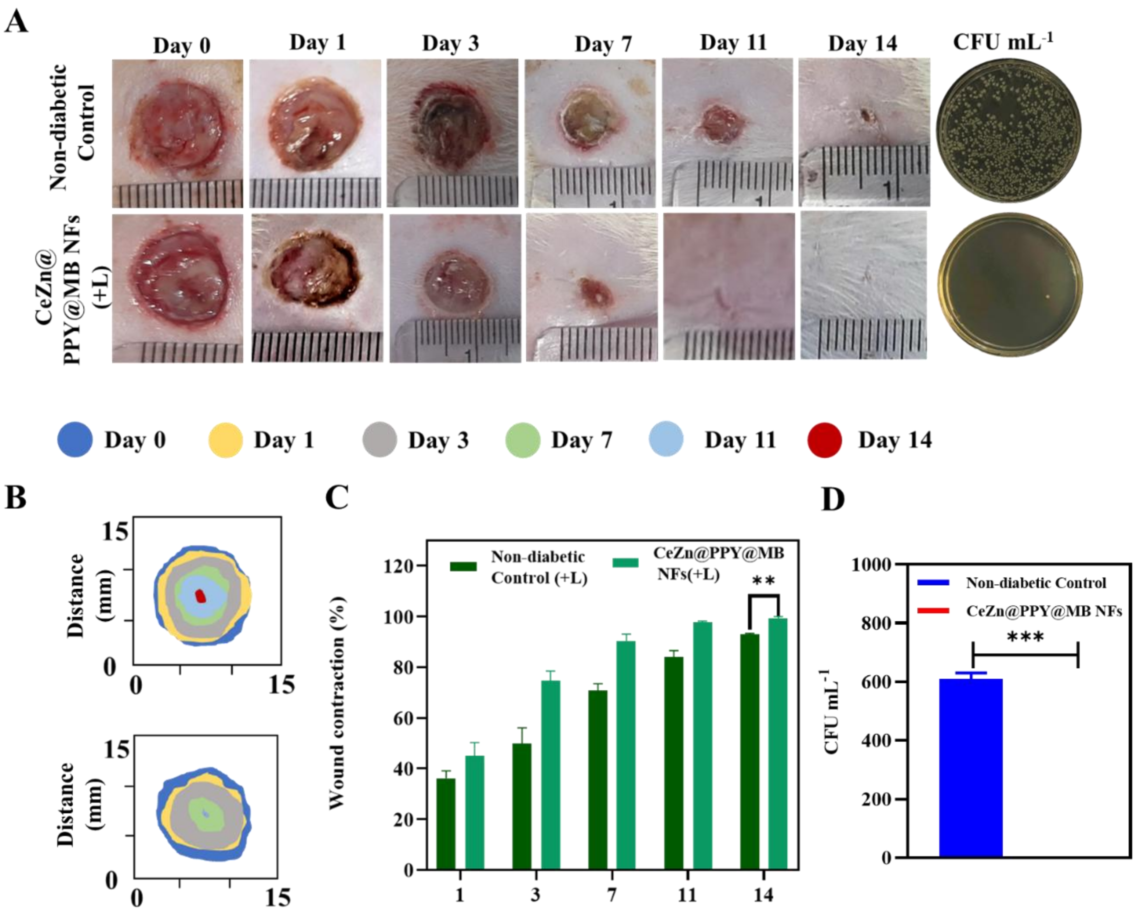

Figure S10. In vivo wound healing efficacy of Ce@PPY@MB NFs in non-diabetic rat wounds, control treated with PBS 7.4; A) images of wounds on different days of treatment, followed by images of MRSA colonies formed after plating the 14th-day wound tissue homogenate, B) trace images of wound closure on days 0, 1, 3, 7, 11, and 14, of non-diabetic control Vs treatment, C) relative wound closure efficacy analysis of different treatments on various days of treatment, D) graph representing the CFU.mL^{-1} value of the plates after plating of wound tissue homogenates after excision, (n=5). All data represented as mean ± SD for sample size n=5 (*p<0.05, **p<0.01, ***p<0.001).

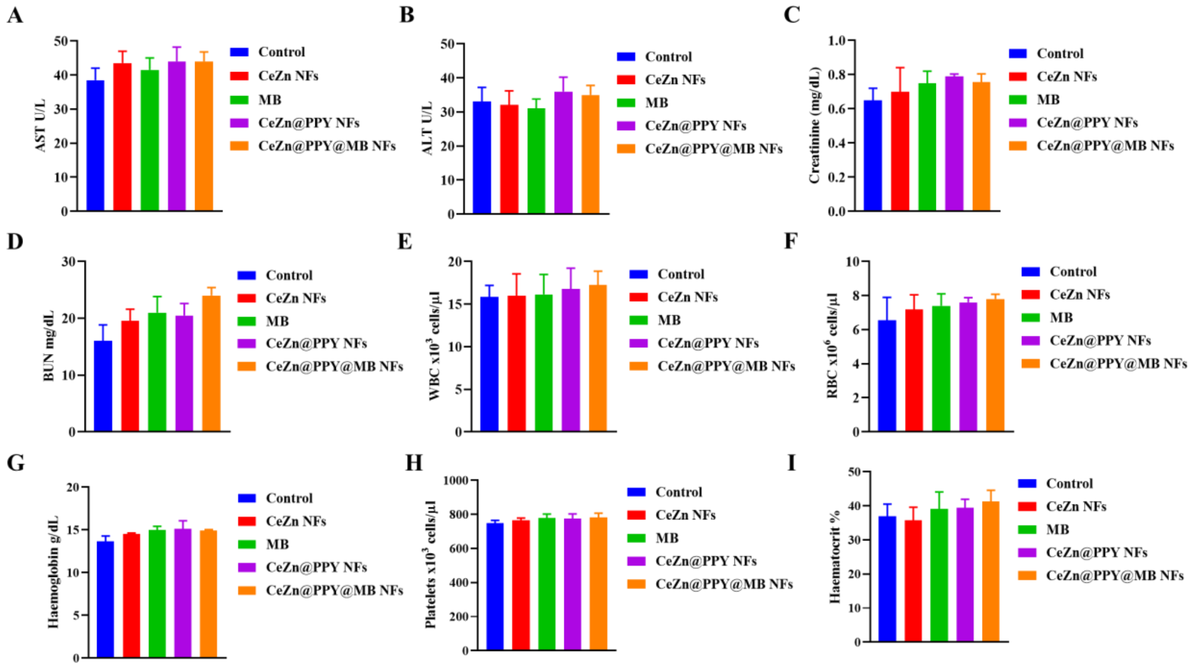

Figure S11. Blood biochemical parameters of the rats from different treatment groups. All data represented as mean ± SD for sample size n=5 (*p<0.05, **p<0.01, ***p<0.001).

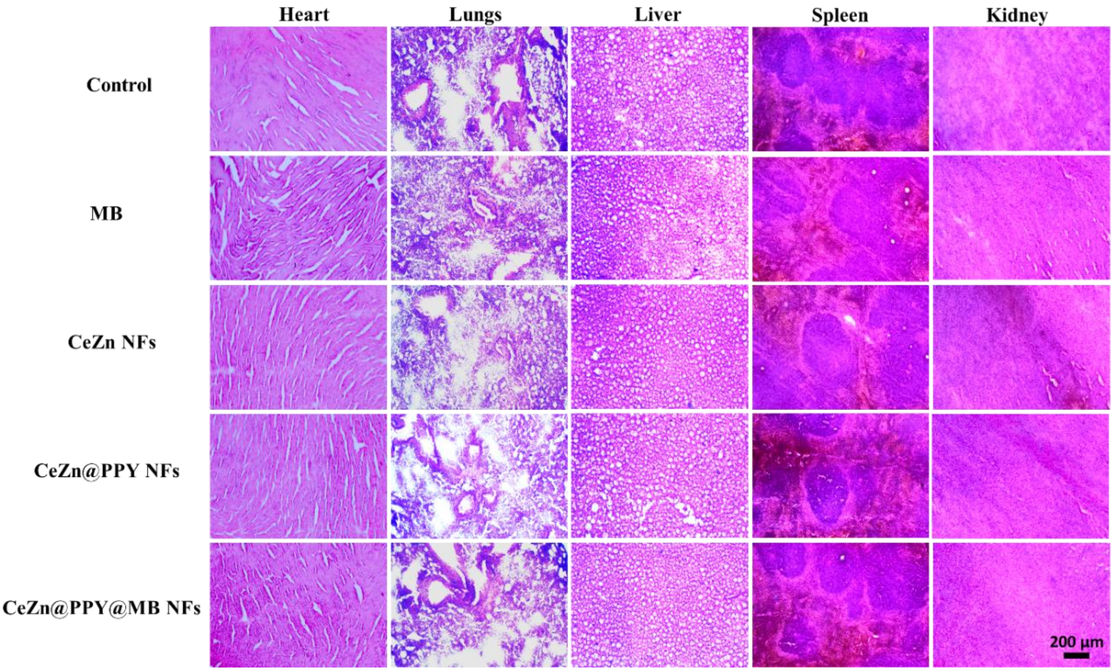

Figure S12. H&E staining images of all major organs of rats of different treatment groups, including heart, lungs, liver, spleen and kidney.

Table S1. The tissue Ce and Zn metal ion concentration

**Elements**	**Released at 5 min (µg)**	**Concentration in 100 mg tissue (ppm)**
Ce	0.373	3.73 ppm

Zn	0.308	3.08 ppm
